# Water‐Soluble Metal Phthalocyanines Enable Molecularly Dispersed Heterogeneous Electrochemical CO_2_ Conversion

**DOI:** 10.1002/advs.202521145

**Published:** 2026-01-21

**Authors:** Xiangyu Zhang, Shoulong Pan, Yu Li, Pan Li, Zizi Ruan, Qing Bai, Qinglin Jiang, Cheng Zhou, Bing Yang, Liang Yao, Yuguang Ma

**Affiliations:** ^1^ State Key Laboratory of Luminescent Materials and Devices Institute of Polymer Optoelectronic Materials and Devices Guangdong Basic Research Center of Excellence for Energy and Information Polymer Materials Guangdong Provincial Key Laboratory of Luminescence from Molecular Aggregates South China University of Technology Guangzhou P. R. China; ^2^ College of New Materials and New Energies Shenzhen Technology University Shenzhen P. R. China; ^3^ State Key Laboratory of Supramolecular Structure and Materials College of Chemistry Jilin University Changchun P. R. China

**Keywords:** CO_2_ reduction, electrocatalysis, heterogeneous molecular catalyst, water‐soluble phthalocyanine

## Abstract

Molecular electrocatalysts offer the prospect of atomically defined active sites and tunable reactivity, yet their integration into heterogeneous systems is frequently hindered by limited site accessibility and reduced catalytic performance. Overcoming molecular aggregation and improving electronic coupling with conductive supports are essential for translating molecular precision into practical electrocatalytic devices. Here we show that water‐soluble, tetrasulfonate‐substituted metallophthalocyanines (MPcTs) spontaneously assemble onto carbon black (CB) through π–π interactions, forming highly dispersed and electronically coupled catalytic sites. The resulting MPcTs/CB catalyst enables highly selective CO_2_‐to‐CO conversion in aqueous media, affording near‐quantitative Faradaic efficiencies and sustained performance over 60 h of continuous operation. Systematic investigations confirm a heterogeneous electrocatalytic pathway, while optimized flow‐cell configurations deliver current densities up to 500 mA cm^−^^2^ with high CO selectivity. This study demonstrates a simple, generalizable strategy to bridge molecular precision and heterogeneous practicality, advancing the design of scalable, high‐performance molecular electrocatalysts for CO_2_ electroreduction in aqueous media.

## Graphical abstract

Water‐soluble metallophthalocyanines self‐assemble onto the surface of carbon black via *π–π* interactions, creating highly dispersed and electronically coupled MPcTs/CB, which enables selective CO_2_‐to‐CO conversion in aqueous media with near‐quantitative Faradaic efficiency and remarkable stability. Optimized flow‐cell operation delivers up to 500 mA cm^−2^ with high CO selectivity, offering a scalable strategy for advanced CO_2_ electrocatalysis.

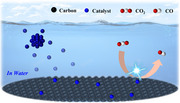

## 1 Introduction

The development of efficient electrocatalysts for electrochemical CO_2_ conversion has attracted considerable attention over the past decades, driven by the growing demand for sustainable technologies that mitigate carbon emissions and enable renewable energy storage [1, 2]. Among the diverse classes of CO_2_ reduction electrocatalysts, molecular catalysts represent a particularly important category due to their well‐defined active sites and precisely tunable structures [3, 4, 5, 6, 7]. These characteristics facilitate fundamental mechanistic studies and enable rational catalyst design and performance optimization based on structure–function relationships [8, 9, 10, 11]. However, when molecular catalysts are incorporated into heterogeneous systems—typically through immobilization on conductive supports or by forming solid‐state composites—the site uniformity that defines their performance in homogeneous environments is often compromised [12, 13, 14]. A primary contributing factor is the strong tendency of highly active molecular catalysts—such as metallated porphyrins and phthalocyanines—to aggregate in the solid state, driven by intermolecular interactions including *π–π* stacking or metal–ligand coordination [15, 16, 17]. This aggregation can severely limit the accessibility of active sites, thereby reducing catalytic efficiency [13, 18, 19, 20, 21, 22, 23]. Furthermore, the preparation of electrodes modified with immobilized molecular catalysts can result in partial encapsulation of the catalytic centers within the support matrix or binding agent, impeding their effective exposure to the reactant medium [24, 25]. These factors not only give rise to a broad distribution of active sites—with varying energetics, reactivity, and selectivity—but also result in inefficient utilization of the molecular catalysts' intrinsic activity [7, 12, 13]. To overcome these challenges and advance the application of molecular electrocatalysts in heterogeneous systems, it is essential to develop novel functional molecular catalysts and immobilization strategies that ensure uniform accessibility of active sites and maintain consistent catalytic behavior at the catalyst–electrolyte interface, thereby maximizing the utilization of active sites in heterogeneous molecular catalysts [26, 27, 28].

**FIGURE 1:**
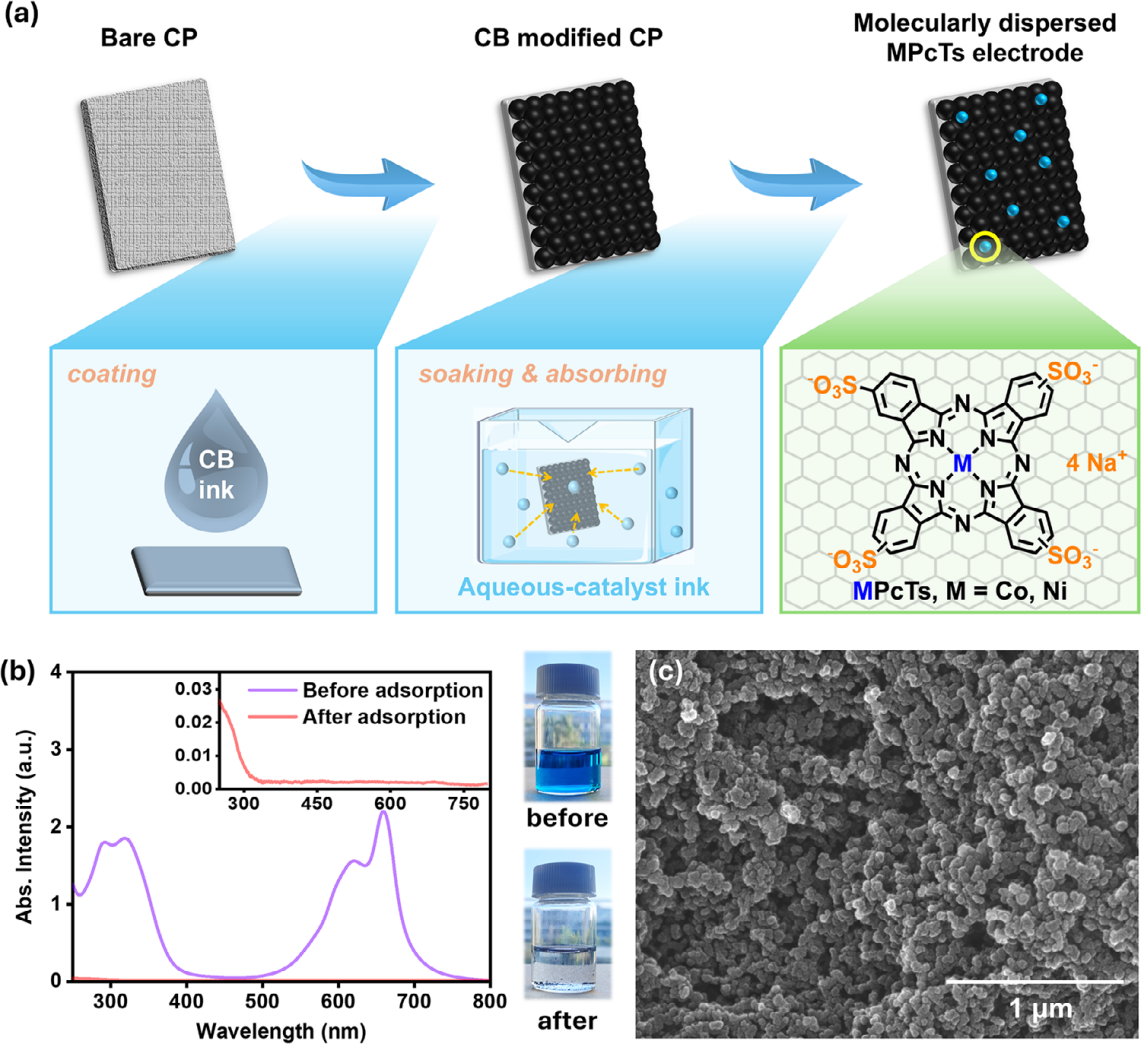
(a) Schematic illustration of the electrode preparation and catalyst coating process. Insert: the molecular structure of MPcTs. (CP = carbon paper, CB = carbon black). (b) UV–vis spectra and corresponding photographs of CoPcTs solution (50 µM L^−1^) before and after adsorption by CB. (c) SEM image of CoPcTs/CB electrode.

In this work, we present a strategy that employs water‐soluble molecular catalysts to construct molecularly dispersed heterogeneous electrocatalysts for electrochemical CO_2_ reduction in an aqueous electrolyte. In this approach, conductive supports are first deposited onto the electrode surface to provide a high‐surface‐area platform for catalyst immobilization. The water‐soluble molecular catalysts, dissolved in aqueous solution, are subsequently introduced and anchored onto the supports via non‐covalent *π–π* interactions at room temperature (Figure 1a). This approach offers several distinct advantages. First, it enables facile and controllable deposition of molecular catalysts onto the conductive support surfaces without the need for high‐temperature treatment or toxic organic solvents, thereby holding significant promise for scalable and practical electrode fabrication. Second, the good solubility of catalysts mitigates catalyst aggregation and minimizes the risk of active sites being buried beneath the support or shielded from the reaction medium, enabling a precise control over catalyst loading and exposure. Moreover, operating the CO_2_ reduction reaction (CO_2_RR) in an aqueous electrolyte facilitates integration with a water oxidation anode via an ion‐exchange membrane, which is critical for constructing fully aqueous electrochemical cells for practical applications [29, 30]. As far as we know, the design and application of water‐soluble molecular catalysts in heterogeneous CO_2_ reduction catalysis remain largely underdeveloped, and reports in the literature are still scarce [31, 32, 33]. Among the limited examples, trimethylammonium‐functionalized metalloporphyrins [31] and metallophthalocyanines [32, 33] have been developed as water‐soluble electrocatalysts for CO_2_ reduction. Nevertheless, these water‐soluble molecular catalysts have been primarily employed in homogeneous catalytic systems [31, 32]. Hailiang Wang and co‐workers synthesized cobalt phthalocyanine derivatives bearing carboxylic acid and trimethylammonium functionalities to impart water solubility, and investigated their electrocatalytic behavior in CO_2_ electroreduction. Although the observed catalytic activity was predominantly heterogeneous in nature, electrochemical adsorption of these molecular catalysts on carbon nanotubes under applied bias was demonstrated to be the driving force to obtain heterogeneous electrocatalysis.

Herein, we designed and synthesized tetrasulfonate‐substituted metallated phthalocyanines as water‐soluble molecular catalysts for electrochemical CO_2_ reduction in aqueous media. Upon immobilization onto the electrodes pre‐modified with conductive support (such as CB), the resulting hybrid electrodes exhibited molecularly dispersed catalytic sites with high activity and selectivity for the electrochemical conversion of CO_2_‐to‐CO. The optimized electrodes achieved a high turnover frequency for CO production (TOF_CO_) of 42.3 s^−1^ at a partial current density of CO production (*j*_CO_) of 160 mA cm^−2^ in the flow cell, highlighting the promising potential of immobilizing water‐soluble molecular catalysts as an effective platform for constructing molecularly dispersed catalytic sites for aqueous‐phase electrocatalysis.

## 2 Results and Discussion

Given the established electrocatalytic activity of nickel(II) and cobalt(II) phthalocyanines for heterogeneous CO_2_ reduction [5, 18], we synthesized tetrasulfonate‐substituted cobalt(II) and nickel(II) phthalocyanines (CoPcTs and NiPcTs), along with the metal‐free analogue (H_2_PcTs), via the cyclotetramerization of sodium 3,4‐dicyanobenzenesulfonate (see Supporting Information for details) [34, 35]. In the following discussion, CoPcTs and NiPcTs are collectively referred to as MPcTs. The successful cyclotetramerization of sodium 3,4‐dicyanobenzenesulfonate under optimized reaction conditions was confirmed by the ^1^H NMR spectrum of H_2_PcTs (Figure S1), which displayed characteristic signals consistent with the expected macrocyclic structure. The molecular weights of MPcTs and H_2_PcTs, as determined by mass spectrometry, were in good agreement with the theoretical values, further verifying the formation of the target compounds. In addition, Fourier‐transform infrared (FT‐IR) spectroscopy confirmed the successful incorporation of sulfonate groups, as evidenced by the characteristic vibrational bands associated with the ─SO_3_^−^ functionalities (Figure S2). The incorporation of tetrasulfonate groups imparts excellent solubility to phthalocyanines in pure water (> 1 mM mL^−^^1^ in pure water and > 0.5 mM mL^−^^1^ in 0.5 m KHCO_3_), thereby enabling the direct immobilization of the tetrasulfonated metallophthalocyanines onto CB supports from their aqueous solutions. As shown in the UV–vis absorption spectra of MPcTs (Figure S3), the Q bands of H_2_PcTs, CoPcTs, and NiPcTs are observed at the range of 550–700 nm, while the corresponding B bands appear at the range of 300–400 nm [36]. Upon the addition of a trace amount of CB to aqueous solutions of MPcTs (50 µM L^−^^1^), the characteristic absorption features of the MPcTs rapidly vanish after gentle shaking (Figure 1b; Figure S4), indicating their effective adsorption of the phthalocyanines onto the CB surface. This strong adsorption behavior is attributed to the pronounced π–π interactions between the conjugated phthalocyanine macrocycles and the aromatic domains of carbon‐based supports such as carbon nanotubes and CB. It is noted that the adsorption amount of MPcTs on CB‐modified electrodes is dependent on the soaking time. Prolonged soaking of CB‐modified electrodes in MPcTs solution leads to a continuous decrease in the UV–vis absorption intensity associated with MPcTs, suggesting progressively higher MPcTs loading on CB (Figure S5). Importantly, scanning electron microscopy (SEM) images and energy‐dispersive X‐ray spectroscopy (EDS) mappings reveal no discernible MPcTs aggregates on the electrode surface for 80 min and even for 720 min of soaking (Figure 1c; Figures S6–S11), indicating a uniform and well‐dispersed deposition of MPcTs onto the CB support.

**FIGURE 2:**
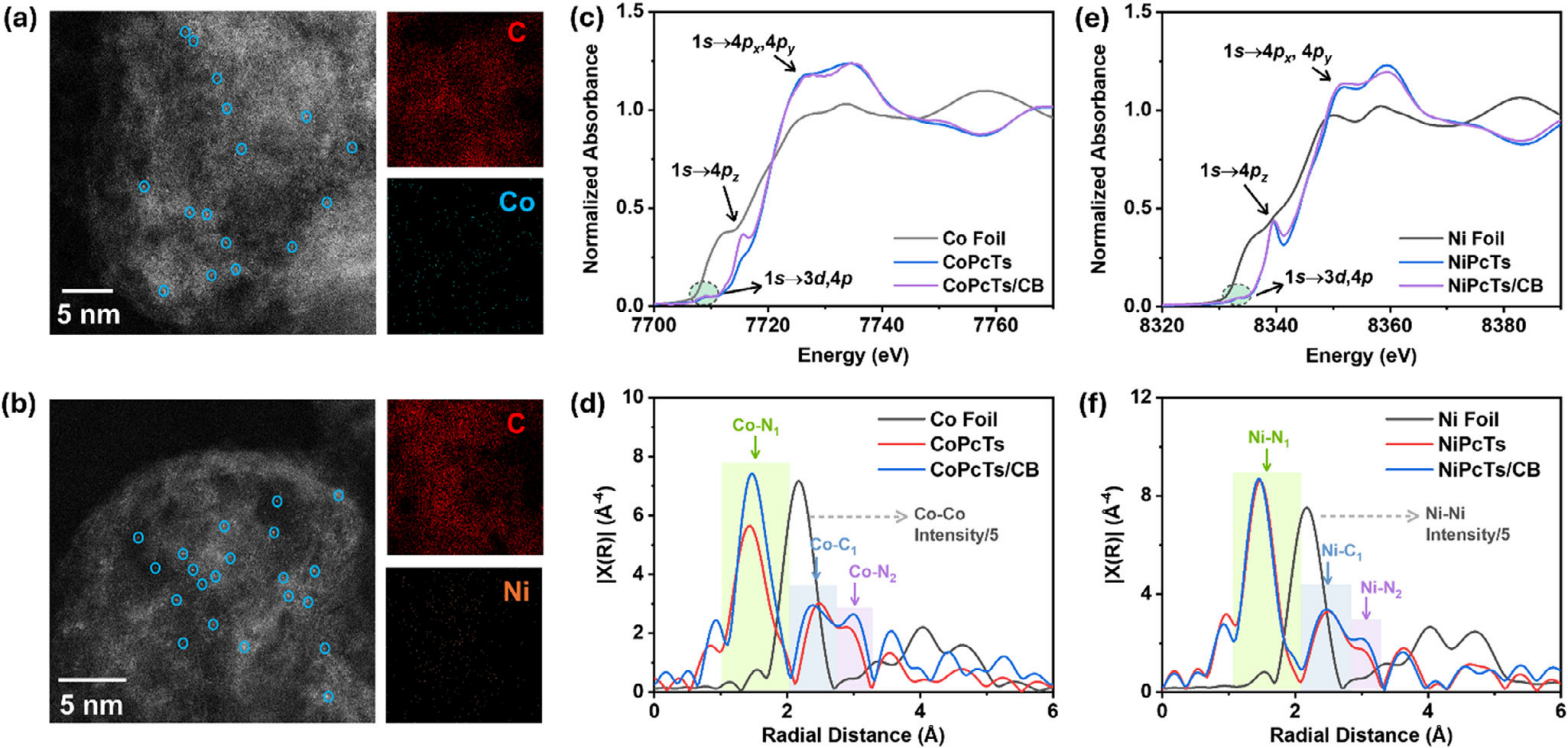
AC‐TEM image of (a) CoPcTs/CB and the corresponding EDS‐mapping of C and Co elements, and (b) NiPcTs/CB and the corresponding EDS‐mapping of C and Ni elements. The circled bright spots highlight the dispersed Co atoms or Ni atoms. (c) Co K‐edge XANES spectra and (d) FT‐EXAFS spectra in R‐space of Co foil, CoPcTs, and CoPcTs/CB. (e) Ni K‐edge XANES spectra and (f) FT‐EXAFS spectra in R‐space of Ni foil, NiPcTs, and NiPcTs/CB.

The molecular‐level dispersion and the local coordination environment of MPcTs on CB were further investigated by transmission electron microscopy (TEM) and X‐ray absorption spectroscopy, respectively. In agreement with the SEM‐EDS results, aberration‐corrected (AC) TEM combined with EDS mapping also demonstrates a uniform distribution of Co and Ni elements across the CB supports (Figures S12 and S13). Moreover, high‐angle annular dark‐field (HAADF) AC‐TEM images display discrete bright spots on the CB surface, indicative of atomically dispersed Co and Ni species (Figure 2a,b). These results collectively corroborate the molecular‐level dispersion of MPcTs on the CB‐modified electrodes. Figure 2c presents the Co K‐edge X‐ray absorption near‐edge structure (XANES) spectra of CoPcTs and CoPcTs/CB, with Co foil included as a reference. Two distinct pre‐edge features are observed for CoPcTs at approximately 7709 and 7715 eV, which can be assigned to predominantly *1s→3d–4p* and *1s→4p_z_* transitions, respectively. The pre‐edge feature of *1s→4p_z_* transition exhibits positive energy shifts relative to Co foil, consistent with the higher oxidation state of cobalt in CoPcTs compared to metallic cobalt. The presence of pre‐edge features is characteristic of a *D_4h_* symmetric Co–N_4_ square‐planar coordination environment, as typically observed in cobalt phthalocyanines (Figure S14) [24, 37, 38]. Furthermore, immobilizing CoPcTs on CB does not cause a significant change in the Co K‐edge XANES spectrum, suggesting that the electronic structure and local coordination environment of the cobalt center are largely preserved upon integration with CB. Similarly, the Ni K‐edge XANES spectra of NiPcTs and NiPcTs/CB display two distinct pre‐edge features at 8333 and 8339 eV, corresponding to the *1s*→*3d–4p* and *1s*→*4p_z_* transition (Figure 2e) [11, 18]. The absence of a significant energy shift upon immobilization on CB suggests that, analogous to the cobalt case, the local coordination environment and oxidation state of the Ni centers remain largely unaffected. The Fourier‐transformed k^3^‐weighted extended X‐ray absorption fine structure (FT‐EXAFS) spectra of CoPcTs/CB and NiPcTs/CB exhibit prominent peaks at approximately 1.5 and 2.5 Å, which can be assigned to Co–N and Ni–N bonds in the first coordination shell, and Co–C and Ni–C bonds in the second coordination shell, respectively (Figure 2d,f; Figure S15 and Table S1) [38, 39, 40, 41, 42]. These well‐defined metal–nitrogen/metal–carbon distances are characteristic of isolated metal centers in a square‐planar M–N_4_ coordination environment, as found in phthalocyanine complexes. Notably, no discernible peaks corresponding to Co–Co or Ni–Ni coordination are observed, indicating the absence of metal aggregation upon immobilization onto CB. These results confirm the successful dispersion of CoPcTs and NiPcTs on CB, with the metal centers retained in well‐isolated coordination environments.

**FIGURE 3:**
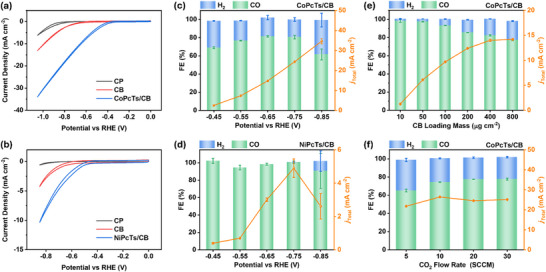
CV curves of bare CP, CB, and (a) CoPcTs/CB (0 ~ –1.05 V vs. RHE) or (b) NiPcTs/CB (0 ~ –0.85 V vs. RHE) respectively as working electrodes in H‐cell. FE_CO_, FE_H2,_ and current density as a function of the applied potentials for (c) CoPcTs/CB and (d) NiPcTs/CB catalysts operated in CO_2_‐saturated 0.5 m KHCO_3_ aqueous solution in H‐cell. FE_CO_, FE_H2,_ and current density as a function of (e) CB loading mass on electrode and (f) CO_2_ flow rate for CoPcTs/CB electrode operated in CO_2_‐saturated 0.5 M KHCO_3_ aqueous solution at −0.75 V vs. RHE in an H‐cell.

The electrochemical CO_2_ reduction performance of MPcTs‐modified electrodes was initially evaluated in a gas‐tight H‐type cell, separated by an anion exchange membrane, using carbon paper (CP) as the working electrode, Ag/AgCl as the reference electrode, and a platinum mesh as the counter electrode (Figure S16). Prior to measurements, the electrolyte (0.5 m KHCO_3_) was saturated with CO_2_ by purging for 15 min, and a continuous CO_2_ flow of 20 SCCM was maintained during electrolysis. MPcTs/CB electrodes were prepared by drop‐casting CB onto CP (400 µg cm^−^^2^), followed by immersion in an aqueous MPcTs solution (50 µmol L^−^^1^) for 80 min. Cyclic voltammetry (CV) was performed to evaluate and compare the electrocatalytic performance of CP, CB‐modified CP (referred to as CB for clarity latter), and MPcTs/CB toward CO_2_RR. As shown in Figure 3a,b, MPcTs/CB exhibited markedly higher catalytic current densities than both bare CP and CB. The onset potentials (defined as the potential at which the current density reaches 1 mA cm^−^^2^) for CoPcTs/CB and NiPcTs/CB were –0.40 and –0.53 V vs. RHE, respectively, both displaying a shift toward positive potentials relative to CB (–0.69 V vs. RHE) and CP (–0.90 V vs. RHE). Faradaic efficiencies (FEs) for CO_2_ conversion were determined by constant‐potential electrolysis over the potential range of −0.45 to −0.85 V vs. RHE. Gas‐phase products (CO and H_2_) were quantitatively analyzed using the external standard method (Figures S17 and S18). NiPcTs/CB achieved FE_CO_ values of 95%–99%, outperforming CoPcTs/CB, which showed FE_CO_ values of 65%–82% (Figure 3c,d; Figures S19–S21). However, CoPcTs/CB delivered higher total current densities, reaching a maximum partial current density for CO (*j*_CO_) of 19.7 mA cm^−^^2^ at –0.75 V vs. RHE—nearly four times greater than that of NiPcTs/CB (4.9 mA cm^−^^2^ at –0.75 V vs. RHE). No liquid products were detected by ^1^H NMR in the potential range of –0.45 to –0.85 V vs. RHE. At more negative potentials (–0.95 to –1.15 V vs. RHE), CoPcTs/CB produced trace amounts of methanol, with a FE_MeOH_ below 1% (Figure S22).

To elucidate the underlying factors responsible for the higher *j*_CO_ yet lower FE_CO_ observed for CoPcTs/CB, we systematically examined the effect of electrode preparation parameters on electrochemical performance. First, the influence of immersion time of the CB‐modified electrode in MPcTs solutions was investigated. For NiPcTs/CB, *j*_total_ increased steadily from 2.5 to 6.6 mA cm^−2^ as the immersion time was extended from 20 to 720 min, while FE_CO_ remained nearly constant (88%−96%) (Figure S23). In contrast, CoPcTs/CB did not exhibit a comparable continuous increase in *j*_total_; instead, FE_CO_ decreased gradually from 89% to 71% over the same immersion‐time range (Figure S24). Given that prolonged soaking increases the CoPcTs loading on CB (Figure S5), these results suggest that the lower FE_CO_ for CoPcTs/CB is not attributable to insufficient catalyst loading. Interestingly, FE_CO_ for CoPcTs/CB exhibited a pronounced dependence on the CB loading on the CP substrate. At a CB loading of 10 µg cm^−2^, the FE_CO_ reached 98% at –0.75 V vs. RHE, whereas increasing the CB loading to 400 µg cm^−2^ reduced FE_CO_ to 82% (Figure 3e; Figure S25). As a lower CB loading inherently decreases the amount of CoPcTs deposited, the total CO_2_ consumption under identical operating conditions is correspondingly reduced, which is also reflected by the CV profiles. These observations suggest that the FE_CO_ of CoPcTs/CB is strongly influenced by CO_2_ mass‐transport limitation in the electrolyte [43]. Such an interpretation is further supported by the observed increase in FE_CO_ with elevated CO_2_ flow rates, as well as the unchanged *j*_CO_ with the use of more negative applied potential (Figure 3f; Figure S26).

**FIGURE 4:**
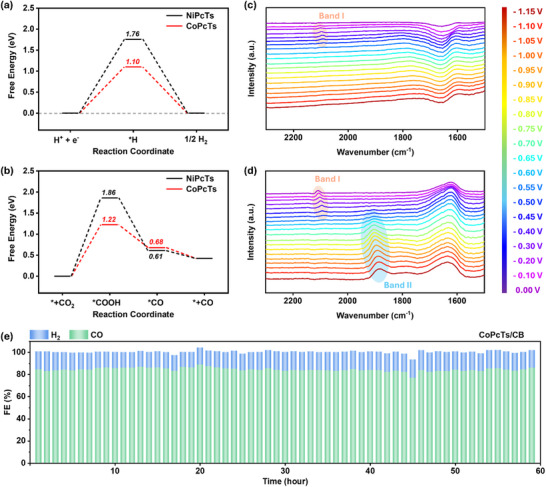
Gibbs free energy diagram of (a) HER and (b) CO_2_RR to CO at 0 V vs. RHE for the NiPcTs/CB and CoPcTs/CB catalysts. ATR‐SEIRAS spectra of (c) CB electrode and (d) CoPcTs/CB measured at 0 V ~ –1.15 V vs RHE in CO_2_‐saturated 0.5 m KHCO_3_ electrolyte. (e) Long‐term stability of FE_CO_ as a function of operating time at −0.75 V vs. RHE.

Density functional theory (DFT) calculations were conducted to gain deeper insight into the electrocatalytic CO_2_RR mechanisms of CoPcTs and NiPcTs. As illustrated in Figure 4a,b, the formation of ^*^COOH intermediate represents the most energetically demanding step for both CoPcTs and NiPcTs, indicating that CO_2_ activation is the rate‐determining step. Notably, CoPcTs exhibits a smaller free energy difference between ^*^+CO_2_ and ^*^COOH compared to NiPcTs, indicating a thermodynamically more favorable formation of the ^*^COOH intermediate. Furthermore, along the hydrogen evolution reaction (HER) pathway, CoPcTs shows a reduced free‐energy difference between H^+^ + e^−^ and ^*^H, suggesting an intrinsically more favorable proton–electron coupling for ^*^H formation. These computational findings are in good agreement with the experimental results, wherein CoPcTs/CB delivers a substantially higher total current density than NiPcTs/CB. The thermodynamic advantages of CoPcTs in facilitating both HER and CO_2_RR pathways likely contribute to the superior overall catalytic activity of CoPcTs/CB.

In situ Fourier‐transform infrared spectroscopy with attenuated total reflectance surface‐enhanced infrared absorption spectroscopy (ATR‐SEIRAS) was employed to monitor key CO_2_RR intermediates and to elucidate the catalytic roles of NiPcTs and CoPcTs, using a bare CB‐modified electrode as a reference. As shown in Figure 4d, CoPcTs/CB exhibits two distinct ^*^CO bands: a band at 2108–2079 cm^−1^ (^*^CO^L^) observed at less negative applied potentials, corresponding to linearly adsorbed CO on quasi‐flat Co–N_4_ centers, and a band at 1903–1882 cm^−1^ (^*^CO^B^) that emerges at more negative potentials, corresponding to bridged CO associated with active CO production [40, 44]. The disappearance of ^*^CO^L^ with increasing cathodic potential suggests a potential‐dependent change in the CO adsorption configuration at the Co–N_4_ active sites, favoring the formation of bridged ^*^CO^B^ species that are more closely associated with active CO production. Lei Wang et al. recently investigated ^*^CO adsorption configurations on CoPc‐based catalysts using a combination of operando XAFS, in situ infrared spectroscopy, and DFT calculations, demonstrating that the formation of ^*^CO^B^ is associated with an out‐of‐plane distortion of the CoPc macrocycle under sufficiently negative potentials [40]. Their results indicated that under the formation conditions of ^*^CO^B^, ^*^CO preferentially adopts a bridged configuration by interacting with two adjacent pyrrolic nitrogen atoms within the Co–N_4_ coordination environment. Similar to CoPcTs/CB, NiPcTs/CB displays the ^*^CO^B^ band at 1940–1894 cm^−1^ under CO‐producing potentials, while the ^*^CO^L^ feature is absent, suggesting weaker CO adsorption at less negative potentials (Figure S27). Notably, the ^*^CO^B^ band of NiPcTs/CB appears at a higher *ν(C–O)* frequency than that of CoPcTs/CB at comparable potentials, signifying a weaker electronic coupling between the Ni–N_4_ center and the ^*^CO intermediate. This weaker interaction results in reduced CO binding strength and more facile CO desorption. These spectroscopic observations are fully consistent with our DFT results, which show a relatively lower ^*^CO desorption free energy for NiPcTs (0.61 eV) compared to CoPcTs (0.68 eV). Collectively, the weaker CO adsorption on NiPcTs alleviates active‐site blockage by accumulated CO and enables sustained CO_2_‐to‐CO turnover, thereby favoring a higher FE_CO_ for NiPcTs. In contrast, CB‐modified electrodes without MPcTs functionalization exhibit only the ^*^CO^L^ band (2108–2079 cm^−1^), which does not significantly contribute to CO production (Figure 4c). These results highlight the critical role of MPcTs in modulating the ^*^CO adsorption configuration and enabling efficient CO_2_‐to‐CO conversion. SEM and HR‐TEM images reveal that no discernible metal particles are formed after electrocatalysis, indicating that no aggregation of metal centers into particulate species is detectable within the spatial resolution of these techniques (Figures S28–S34). Moreover, we note that CoPcTs/CB exhibits an excellent stability toward CO production, maintaining an average FE_CO_ above 83% over at least 60 h of continuous operation at −0.75 V vs. RHE (Figure 4e; Figure S35). This durability originates from the robust Co–N_4_ macrocyclic coordination and strong *π–π* interactions with the carbon support, which jointly stabilize molecularly dispersed active sites, while the high aqueous solubility of CoPcTs suppresses aggregation during catalyst immobilization. To evaluate the impact of loading strategy on catalytic performance, we compared two approaches for incorporating CoPcTs. The first approach—employed and discussed in the preceding sections—involved immersing a CB‐modified electrode in an aqueous CoPcTs solution, enabling in situ adsorption of CoPcTs onto CB (*immersion‐based adsorption*). The second approach consisted of pre‐mixing CB with CoPcTs at the same cobalt loading as in the first approach, followed by drop‐casting the resulting CoPcTs/CB composite onto a CP electrode (*pre‐mixed composite drop‐casting*). As shown in Figure S36, the *immersion‐based adsorption* strategy delivered both higher current densities and superior operational stability compared to the *pre‐mixed composite*. This enhancement can be attributed to a greater exposure of CoPcTs catalytic sites, as the adsorption process avoids the burial of active sites that may occur during the drop‐casting procedure.

**FIGURE 5:**
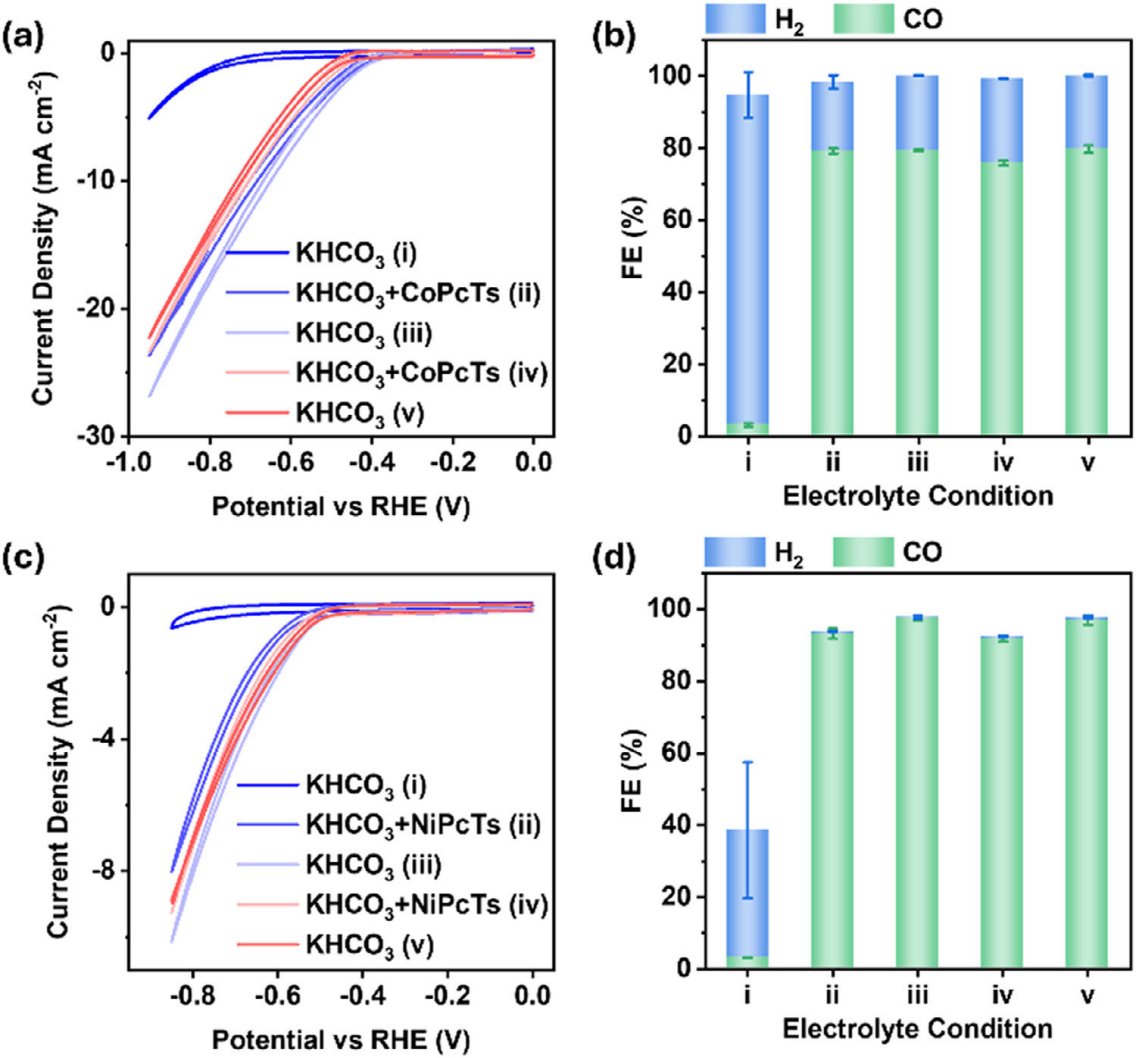
CV curves (a,c) and FE profiles (b,d) recorded on CB‐modified electrodes under alternating electrolyte conditions of 0.5 M KHCO_3_ and 50 µM MPcTs + 0.5 M KHCO_3_. Panels (a,b) correspond to CoPcTs, while (c,d) correspond to NiPcTs. The electrolyte sequence was: (i) 0.5 M KHCO_3_, (ii) 50 µm MPcTs + 0.5 m KHCO_3_, (iii) 0.5 m KHCO_3_, (iv) 50 µM MPcTs + 0.5 m KHCO_3,_ and (v) 0.5 m KHCO_3_. Faradaic efficiencies were determined at a constant potential of −0.75 V vs. RHE for CoPcTs and −0.65 V vs. RHE for NiPcTs.

Given that MPcTs are intrinsically water‐soluble and the electrolyte medium is aqueous, it is important to clarify whether the observed electrocatalysis proceeds via a homogeneous pathway in solution or through a heterogeneous process mediated by MPcTs adsorbed on the carbon support [33, 45]. To elucidate that, we measured CB‐modified electrodes in alternating electrolytes between 0.5 m KHCO_3_ and 50 µM MPcTs + 0.5 M KHCO_3_ (Figure 5; Figure S37). In 0.5 m KHCO_3_, CV of a bare CB‐modified electrode showed a negligible current density of <5 mA cm^−2^ and a FE_CO_ of 3% at −0.75 V vs. RHE, indicating minimal CO_2_ reduction and predominantly hydrogen evolution. Upon introducing CoPcTs contained in KHCO_3_ electrolyte, the current density increased significantly to 14 mA cm^−2^, with a FE_CO_ of 79% at the same potential. The electrode was then retrieved, rinsed thoroughly, and re‐immersed in fresh 0.5 m KHCO_3_ without soluble catalyst. Remarkably, both current density and FE_CO_ were maintained at levels comparable to the measurement in mixed electrolyte, and repeated switching cycles yielded consistent results. NiPcTs exhibited analogous behavior at −0.65 V vs. RHE, with the notable observation of negligible H_2_ evolution during the switching process. These results demonstrate that the observed CO_2_ reduction originates from heterogeneous catalysis by MPcTs molecules adsorbed onto the CB surface via *π–π* interactions, rather than from homogeneous catalysis in solution.

**FIGURE 6:**
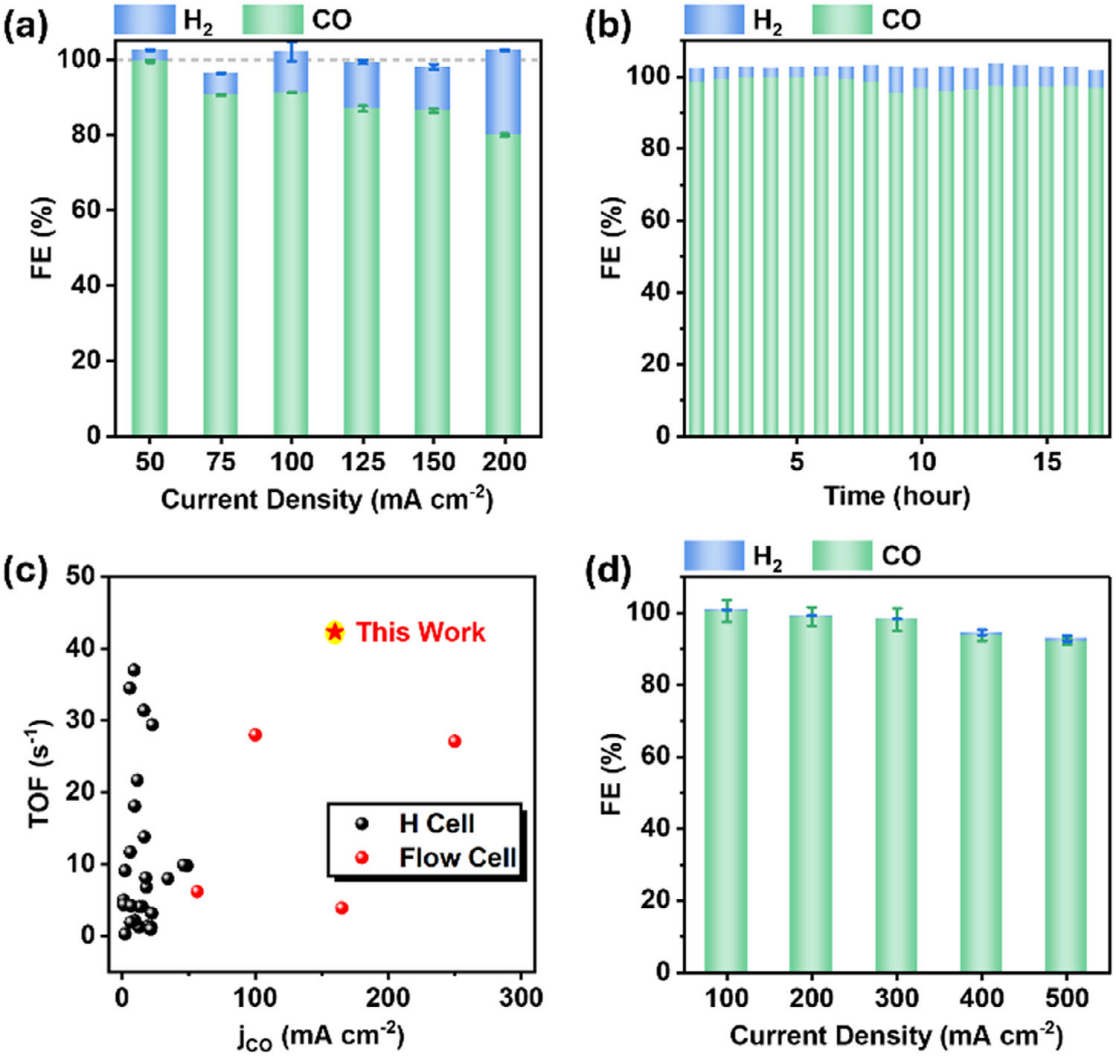
(a) FE_CO_ and FE_H2_ of CoPcTs/CB catalyst at different current densities (50−200 mA cm^−2^) and (b) long‐term stability and corresponding FE_CO_ and FE_H2_ of CoPcTs/CB catalyst at 50 mA cm^−2^ in 1 M KHCO_3_ electrolytes in a flow cell (the electrodes were prepared through a soaking procedure). (c) The comparison of TOF in this work and the reported works. (d) FE_CO_ and FE_H2_ of CoPcTs/CB/PTFE catalyst (mass ratio = 1:15:1) at different current densities (100−500 mA cm^−2^) in 1 M KHCO_3_ electrolytes in a flow cell (the electrodes were prepared through a pre‐composite procedure).

To assess the practical applicability of MPcTs catalysts in high‐current‐density CO_2_ reduction electrolyzers, we incorporated them into gas diffusion electrodes (GDEs) [27, 39]. Given that CoPcTs exhibited superior CO_2_ reduction activity and higher attainable current density compared to NiPcTs, only CoPcTs were subsequently employed for evaluation in the GDE configuration. The electrode preparation followed a similar procedure to that used for H‐cell experiments: a CB layer was spray‐coated onto CP electrodes with a microporous layer, followed by immersion of the resulting electrode in a CoPcTs solution (50 µM) for 80 min. The resulting CoPcTs‐modified CB electrode was then assembled into a GDE cell comprising a gas diffusion chamber, catholyte chamber, and analyte chamber, as illustrated in Figure S38. Using 1 M KHCO_3_ as the electrolyte, chronopotentiometric measurements were conducted at current densities ranging from 50 to 200 mA cm^−2^. The FE_CO_ reached 99% at 50 mA cm^−2^ but decreased to 80% at 200 mA cm^−2^, accompanied by an increase in hydrogen evolution reaction, which is primarily attributed to electrode flooding at high current densities (Figure 6a). Notably, a high FE_CO_ of >95% was sustained for 17 h of continuous operation at 50 mA cm^−2^, with the corresponding potential exhibiting no significant drift over the course of the test (Figure 6b; Figure S39). The cobalt loading, determined by inductively coupled plasma‐optical emission spectrometry (ICP‐OES), was measured to be 19.5 nmol cm^−2^. Assuming 100% active‐site utilization, the CoPcTs/CB electrode achieved a CO turnover frequency (TOF_CO_) of 42.3 s^−1^ at 200 mA cm^−2^ (*j*_CO_ of 160 mA cm^−2^), among the best reported values (Figure 6c; Table S2), thereby demonstrating the simultaneous attainment of high TOF_CO_ and large *j*_CO_. At this ultralow catalyst loading, the limited availability of catalytic sites and flooding effects at high current densities resulted in an FE_CO_ of ~80%, modestly lower than that of some reported systems (Table S2). To mitigate flooding and achieve higher current densities with the CoPcTs catalyst, polytetrafluoroethylene (PTFE) was incorporated into the CoPcTs/CB catalyst layer to impart hydrophobicity and facilitate CO_2_ diffusion to the active sites, while the CoPcTs loading amount was also increased [46]. This optimization effectively alleviated the flooding issue and enabled current densities up to 500 mA cm^−2^, with FE_CO_ values of 99% at 200, 98% at 300, and 92% at 500 mA cm^−2^ (Figure 6d). Although flooding was still observed at 500 mA cm^−2^, high CO selectivity and reasonable stability were obtained at 100 mA cm^−2^ (FE_CO_ of ~100% over 8 h) and at 200 mA cm^−2^ (FE_CO_ of >98% over 5 h) (Figures S41–S43). These results demonstrate that MPcTs, along with other water‐soluble molecular catalysts, hold considerable promise for enabling high‐performance CO_2_ reduction electrolyzers. Moreover, the detection of methanol over CoPcTs (Figure S22) suggests that this catalyst platform may be extended beyond CO production, and ongoing efforts are focused on tuning the conductive support to enhance multi‐electron CO_2_ reduction toward more deeply reduced products.

## 3 Conclusion

In summary, this work establishes water‐soluble metallophthalocyanines as a versatile platform for constructing heterogeneous CO_2_ reduction electrocatalysts that combine molecular‐level precision with high performance in aqueous media. By exploiting the intrinsic aqueous solubility of MPcTs, a simple immersion of carbon black‐modified electrodes enables spontaneous, non‐covalent adsorption to form molecularly dispersed and well‐exposed active sites, without the need for toxic organic solvents, high‐temperature treatments, or energy‐intensive processing. This strategy effectively suppresses catalyst aggregation and maximizes active‐site accessibility, thereby enabling highly selective CO_2_‐to‐CO conversion with Faradaic efficiencies approaching 100%. The CoPcTs/CB system, in particular, exhibited remarkable durability, maintaining an average FE_CO_ above 83% over 60 h of continuous operation. Alternating electrolyte between 0.5 M KHCO_3_ and 50 µM MPcTs + 0.5 M KHCO_3_ confirms the CO_2_RR performance was independent of the presence of MPcTs molecules in aqueous electrolyte, indicating that the reaction proceeded via a heterogeneous catalytic pathway. Importantly, CoPcTs/CB achieves a TOF(CO) of 42.3 s^−^^1^ at 200 mA cm^−^^2^ in a flow cell configuration, demonstrating the simultaneous realization of large *j*_CO_ and efficient catalytic site utilization. Further optimization enables current densities up to 500 mA cm^−^^2^ while maintaining a high CO selectivity of 92%. Collectively, these findings underscore the potential of MPcTs and other water‐soluble molecular catalysts to bridge molecular‐level tunability with practical device‐level performance, offering a promising avenue for the development of next‐generation electrocatalysts.

## Conflicts of Interest

The authors declare no conflicts of interest.

## Supporting information




**Supporting File**: advs73948‐sup‐0001‐SuppMat.docx.

## Data Availability

The data that support the findings of this study are available from the corresponding author upon reasonable request.
