# Supplementary material for: Water‐Soluble Metal Phthalocyanines Enable Molecularly Dispersed Heterogeneous Electrochemical CO2 Conversion
Source: Adv Sci (Weinh). 2026 Jan 21;13(18):e21145. doi: 10.1002/advs.202521145 (PMC13042911; doi:10.1002/advs.202521145)
Supplement: Supplementary file 1 — Supporting File: advs73948‐sup‐0001‐SuppMat.docx. [file ADVS-13-e21145-s001.docx]

**Supporting Information**

**Water-Soluble Metal Phthalocyanines Enable Molecularly Dispersed Heterogeneous Electrochemical CO_2_ Conversion**

Xiangyu Zhang^1^, Shoulong Pan^1^, Yu Li^1^, Pan Li^1^, Zizi Ruan^1^, Qing Bai^2^, Qinglin Jiang^1^, Cheng Zhou^1^, Bing Yang^3^, Liang Yao^1^*, Yuguang Ma^1^

^1^State Key Laboratory of Luminescent Materials and Devices, Institute of Polymer Optoelectronic Materials and Devices, Guangdong Basic Research Center of Excellence for Energy and Information Polymer Materials, Guangdong Provincial Key Laboratory of Luminescence from Molecular Aggregates

South China University of Technology

Guangzhou 510640, P.R. China

Email: [liangyao@scut.edu.cn](mailto:liangyao@scut.edu.cn)

^2^College of New Materials and New Energies

Shenzhen Technology University

Shenzhen 518118, P. R. China

^3^State Key Laboratory of Supramolecular Structure and Materials, College of Chemistry

Jilin University

Changchun 130012, P.R. China

Experimental Procedures

***Synthetic details***

**Synthesis of sodium** **phthalocyanine tetrasulfonate (H_2_PcTs).**

**Scheme S1.** Synthetic route of H_2_PcTs.

The sodium 3,4-dicyanobenzenesulfonate (202.30 mg, 0.88 mmol) was added to a 100 mL single-necked round-bottom flask. Subsequently, *n-*hexanol (20 mL) and 1,8-diazabicyclo[5.4.0]undec-7-ene (DBU, 0.5 mL) were introduced successively. The reaction mixture was heated under reflux at 155 °C for 36 h under a nitrogen atmosphere. After cooling to room temperature, excess petroleum ether (PE) was added to precipitate the solid product, which was collected by centrifugation. The solid was dissolved in ultrapure water, and the pH was adjusted to 10 using 1 M NaOH solution. The resulting mixture was centrifuged, and the supernatant was collected. The pH of the supernatant was then adjusted to 7 with 1 M HCl solution, followed by dialysis to remove residual DBU. The solvent was subsequently removed via rotary evaporation under reduced pressure. The crude product was purified by reversed-phase silica gel column chromatography to afford a dark green powder (96.10 mg, 47% yield). HR-MS, EI (mass m/z): 221.37 [M^4-^]，295.68 [HM^3-^]，444.25 [H_2_M^2-^]. ^1^H NMR (400 MHz, DMSO) δ = 9.69, 9.48, 9.43, 8.51 ppm. FT-IR: 418.0, 598.8, 650.4, 696.2, 750.2, 836.5, 913.6, 1030.3, 1109.4, 1146.0, 1184.6, 1336.4, 1385.1, 1483.0, 1632.4, 2850.8, 2919.2 cm^-1^. UV-vis: 336.5, 612.5, 639.0, 676.5 nm.

**Synthesis of sodium cobalt phthalocyanine tetrasulfonate (CoPcTs).**

**Scheme S2.** Synthetic route of CoPcTs.

A mixture of sodium 3,4-dicyanobenzenesulfonate (230.13 mg, 1.0 mmol) and anhydrous cobalt (II) acetate (53.11 mg, 0.3 mmol) was added to a 100 mL single-necked round-bottom flask. Subsequently, *n-*hexanol (20 mL) and 1,8-diazabicyclo[5.4.0]undec-7-ene (DBU, 0.5 mL) were introduced successively. The reaction mixture was heated under reflux at 155 °C for 36 h under a nitrogen atmosphere. After cooling to room temperature, excess petroleum ether (PE) was added to precipitate the solid product, which was collected by centrifugation. The solid was dissolved in ultrapure water, and the pH was adjusted to 10 using 1 M NaOH solution. The resulting mixture was centrifuged, and the supernatant was collected. The pH of the supernatant was then adjusted to 7 with 1 M HCl solution, followed by dialysis to remove residual DBU. The solvent was subsequently removed via rotary evaporation under reduced pressure. The crude product was purified by reversed-phase silica gel column chromatography to afford a dark green powder (83.27 mg, 34% yield). HR-MS, EI (mass m/z): 221.72 [M^4-^], 295.96 [HM^3-^], 444.45 [H_2_M^2-^]. FT-IR: 413.2, 595.4, 649.4, 699.6, 748.7, 834.1, 900.1, 933.9, 969.1, 1028.4, 1109.8, 1191.8, 1330.2, 1400.1, 1528.8, 1627.6, 1717.8 cm^-1^. UV-vis: 334.5, 612.0, 678.0 nm.

**Synthesis of sodium nickel phthalocyanine tetrasulfonate (NiPcTs).**

**Scheme S3.** Synthetic route of NiPcTs.

A mixture of sodium 3,4-dicyanobenzenesulfonate (230.65 mg, 1.0 mmol) and anhydrous nickel (II) acetate (53.26 mg, 0.3 mmol) was added to a 100 mL single-necked round-bottom flask. Subsequently, *n-*hexanol (20 mL) and 1,8-diazabicyclo[5.4.0]undec-7-ene (DBU, 0.5 mL) were introduced successively. The reaction mixture was heated under reflux at 155 °C for 36 h under a nitrogen atmosphere. After cooling to room temperature, excess petroleum ether (PE) was added to precipitate the solid product, which was collected by centrifugation. The solid was dissolved in ultrapure water, and the pH was adjusted to 10 using 1 M NaOH solution. The resulting mixture was centrifuged, and the supernatant was collected. The pH of the supernatant was then adjusted to 7 with 1 M HCl solution, followed by dialysis to remove residual DBU. The solvent was subsequently removed via rotary evaporation under reduced pressure. The crude product was purified by reversed-phase silica gel column chromatography to afford a dark green powder (79.63 mg, 33% yield). HR-MS, EI (mass m/z): 221.47 [M^4-^], 295.02 [HM^3-^], 443.95 [H_2_M^2-^]. FT-IR: 431.0, 564.1, 625.3, 707.3, 750.2, 809.0, 840.0, 926.6, 1027.9, 1061.6, 1108.4, 1172.0, 1225.5, 1325.8, 1395.2, 1479.6, 1514.8, 1612.2, 1717.3 cm^-1^. UV-vis: 334.0, 601.0, 668.5 nm.

***Chemicals***

The chemicals and solvents used for synthesis and characterization were purchased from Energy Chemical and used without further purification. The products were purified through reversed-phase silica gel column chromatography using a rapid preparative liguid chromatograph SepaBean machine. KHCO_3_ (99.99%) and Nafion (117) solution used for electrocatalysis and electrode preparation were purchased from Aladdin. Ultrapure water (18.2 MΩ cm) used for electrocatalysis was prepared by Sartorius Arium Mini device.

***Characterization***

High-resolution mass spectrometry (HR-MS, ESI) spectra were obtained using an Agilent 1290 Mass Spectrometer. The Fourier Transform Infrared Spectroscopy (FT-IR) was measured with Thermo Fisher Scientific Nicolet iS50 FT-IR. Proton Nuclear Magnetic Resonance spectra (^1^H NMR) were recorded with a Bruker AVANCE III HD 400 MHz instrument for structural characterization and Bruker AVANCE III HD 600 MHz instrument for liquid product quantification, with tetramethylsilane (TMS) as an internal standard. Ultraviolet-visible-near infrared (UV-vis-NIR) measurements were recorded using Shimadzu UV-3600 spectrometer and a 1 cm optical path length quartz cuvette at 298 K. The solvents used for all tests are HPLC grade. Scanning electron microscopy (SEM) was performed by Zeiss Merlin field emission scanning electron microscope. Transmission electron microscopy (TEM) was performed by Thermo Fisher Talos F200X G2 transmission electron microscope. Aberration correction transmission electron microscopy (AC-TEM) images were obtained from JEM-ARM200F operating at 200 kV. Electrocatalysis measurements were performed by CHI 1140D workstation and gas product quantification was performed by GC 9790 plus. X-ray absorption fine structure (XAFS) and extended x-ray absorption fine structure (EXAFS) spectra were recorded at the BL17B beamline, Shanghai Synchrotron Radiation Facility (SSRF).

***Electrode*** ***preparation***

For electrochemical experiments in H-cell, the electrode preparation process was recorded as a two-step process shown in Figure 1. Firstly, an ink of CB was prepared by dispersing 10 mg of CB in 5 mL of ethanol with 50 uL of 5 wt% Nafion solution, followed by sonication for 1 h. 200 μL of the ink was then drop-casted onto a carbon fibre paper for two sides (Toray 060) to cover a carbon paper area of 1 × 1 cm^2^ (CB mass loading is 400 μg cm^−2^). The prepared bare CB electrodes were fully dried for 12 h using a vacuum oven. Then, a catalyst solution was prepared by dissolving 5 mg of CoPcTs and 5 g of KHCO_3_ in 100 mL of ultrapure water. The bare CB electrodes were soaked in the catalyst solution for 80 min, followed by subsequent rinsing steps in ethanol (10 mL, 15−30 s) and ultrapure water (10 mL, 15−30 s) for three times each. In soaking time optimization experiments, this duration was adjusted to various values (20, 40, 60, 80, 100 and 720 min). In CB loading amount optimization experiments, this loading amount was adjusted to various values (10, 50, 100, 200, 400 and 800 μg).

For electrochemical experiments in flow cell, the carbon fiber paper of Toray 060 was replaced by SGL 28BC. The CB ink was sprayed onto an area of 1.5 × 1.5 cm^2^ carbon fiber paper through a Sinero SCP102 ultrasonic spray coating machine (the CB loading amount was controlled at 300 ± 20 mg cm^−2^). Other operations were performed as above.

***Sample preparation for XAFS and EXAFS***

Samples obtained via a single soaking process, identical to the electrode preparation method, exhibited insufficient CoPcTs/CB and NiPcTs/CB loading for reliable XANES and EXAFS data. Consequently, to achieve higher loading for these analytical techniques, CoPcTs/CB and NiPcTs/CB samples were prepared using a multi-step soaking method in aqueous solutions. The procedure involved adding 60 mg of CB to 10 mL of a 50 μM CoPcTs/NiPcTs solution and stirring for 30 minutes. The resulting solid was collected by centrifugation, a process repeated three times. It was then washed sequentially three times with water and three times with ethanol. Ultimately, 50 mg of the composite samples were pelleted with 20 mg of BN as a binder for XANES and EXAFS measurements.

***Electrochemical measurements.***

Electrochemical experiments were performed using a CHI 1140D Potentiostat and a commercial gas-tight two-compartment H-type electrochemical cell (Gauss Union C007-2 electrolytic cell). A platinum net counter electrode (1 × 1 cm^2^) and an Ag/AgCl reference electrodes (filled with saturated KCl solution), both suitable for the C007-2 electrolytic cell, were purchased from Gauss Union. The Ag/AgCl reference electrode was calibrated before use to avoid possible reference potential shifts. The cathode and anode compartments were separated by an anion-exchange membrane (Fumasep FAB-PK-130). Unless otherwise stated, the electrolyte was 0.5 M KHCO_3_ aqueous solution saturated with high purity CO_2_ (pH 7) by bubbling the gas for at least 15 min before each measurement. Gas was continuously bubbled into the electrolyte during electrolysis at a flow rate of 20 standard cubic centimetres per minute (SCCM). Current densities were calculated on the basis of the carbon fiber paper geometric area of the working electrode. All potentials (V) were converted to the RHE scale using the following formula without *iR* compensation:

*E*_RHE_ = *E*_Ag/AgCl_ + (0.199 V) + (0.0592 V) × *pH*.

To evaluate the influence of *iR* drop, the uncompensated resistance (*R*_u_) was determined by electrochemical impedance spectroscopy (EIS) using an equivalent circuit consisting of *R*_u_ in series with a parallel combination of a constant phase element and a resistor. In the H-cell configuration, fitting of the EIS data at −0.2 V and −0.6 V vs RHE showed *R*_u_ = 4.5 Ω, whereas in the flow-cell configuration, fitting of curves at −0.2 V and −0.4 V vs RHE gave *R*_u_ = 3.7 Ω.

Evaluation of turnover frequency (TOF, s^-1^). The TOF to produce CO could be calculated based on the following equation:

$$TOF_{CO}=\frac{j_{CO}}{nF\cdot N}$$

Where *j_CO_* is the partial current density of CO production, *n* is the number of electrons transferred for CO formation (*n* = 2), *F* is the Faradaic constant, *N* is the total surface concentration of adsorbed molecular catalyst on the electrode, which is determined by ICP-OES. (Noted: TOF_H2_ was calculated based on the same equation where *j_CO_* was replace as *j_H2_*.)

***Product quantification***

The gas products of electrocatalysis were analysed using a gas chromatography system (GC 9790 plus) equipped with a flame ionization detector and a thermal conductivity detector. High-purity CO_2_ was used as the carrier gas. The peak areas of the products (H_2_ and CO) were converted to gas volumes using calibration curves that were obtained using a standard gas diluted with CO_2_ to different concentrations. The liquid products were quantified after electrocatalysis using ^1^H NMR spectroscopy with solvent (H_2_O) suppression. A mix solution including 330 μL of electrolyte, 250 μL of D_2_O and 20 μl of a solution of 25 mM of dimethyl sulfoxide (DMSO) as internal standard was prepared for the ^1^H NMR analysis. The concentration of MeOH was calculated using the ratio of the area of the MeOH peak (at a chemical shift of 3.263 ppm) to that of the DMSO internal standard (see Figurer S10). The *FE*s for the gas-phase products were average values from three measurements in a single electrolysis experiment, and the *FE_MeOH_* values at all potentials were measured once.

***Theoretical calculation***

All density functional theory (DFT) calculations were carried out using the Gaussian 16 (version A.03) package on the High Performance Computing Platform of South China University of Technology.^[1]^ The PBE0 functional^[2]^ with the D3 empirical correction (Becke–Johnson damping) ^[3]^ was used for London dispersion forces, which make it widely accepted as the proper functional to study the reactions of transition metal complexes.^[4]^ The def2-SVP basis set was used for geometry optimization calculations and vibrational frequency calculations, and the def2-TZVP basis set was used for single point energy calculations.^[5,6]^ All reaction intermediates were fully optimized with no imaginary frequency. The Gibbs free energies (G) were determined based on optimized structures at 298.15 K and 1 atm, employing the harmonic potential approximation. The Shermo program, along with a specific correction factor for the PBE0-D3/def2-TZVP method, was utilized for this purpose.^[7]^ The electrocatalytic mechanisms were studied based on Nørskov’s computational hydrogen electrode model.^[8]^ In this technique, zero voltage was defined based on the potential energy (μ) of compotents involved in the reversible hydrogen electrode at all pH, T and *p*, and μ(H^+^) + μ(e^–^) = 1/2 μ(H_2_) at a potential of 0 V.

Supplementary Figures


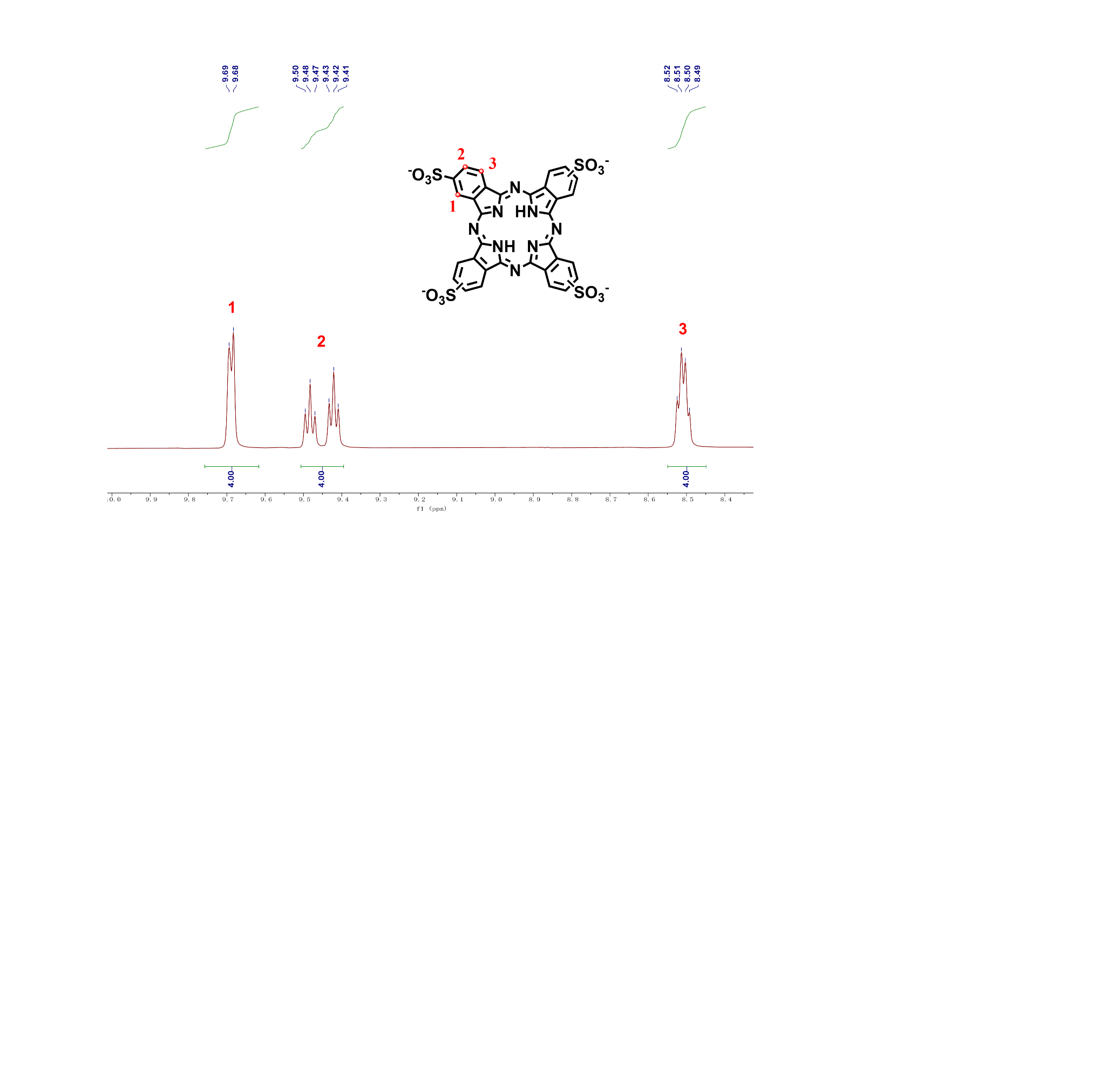


**Figure S1.**  ^1^H NMR spectrum of H_2_PcTs (in *d_6_*-DMSO).


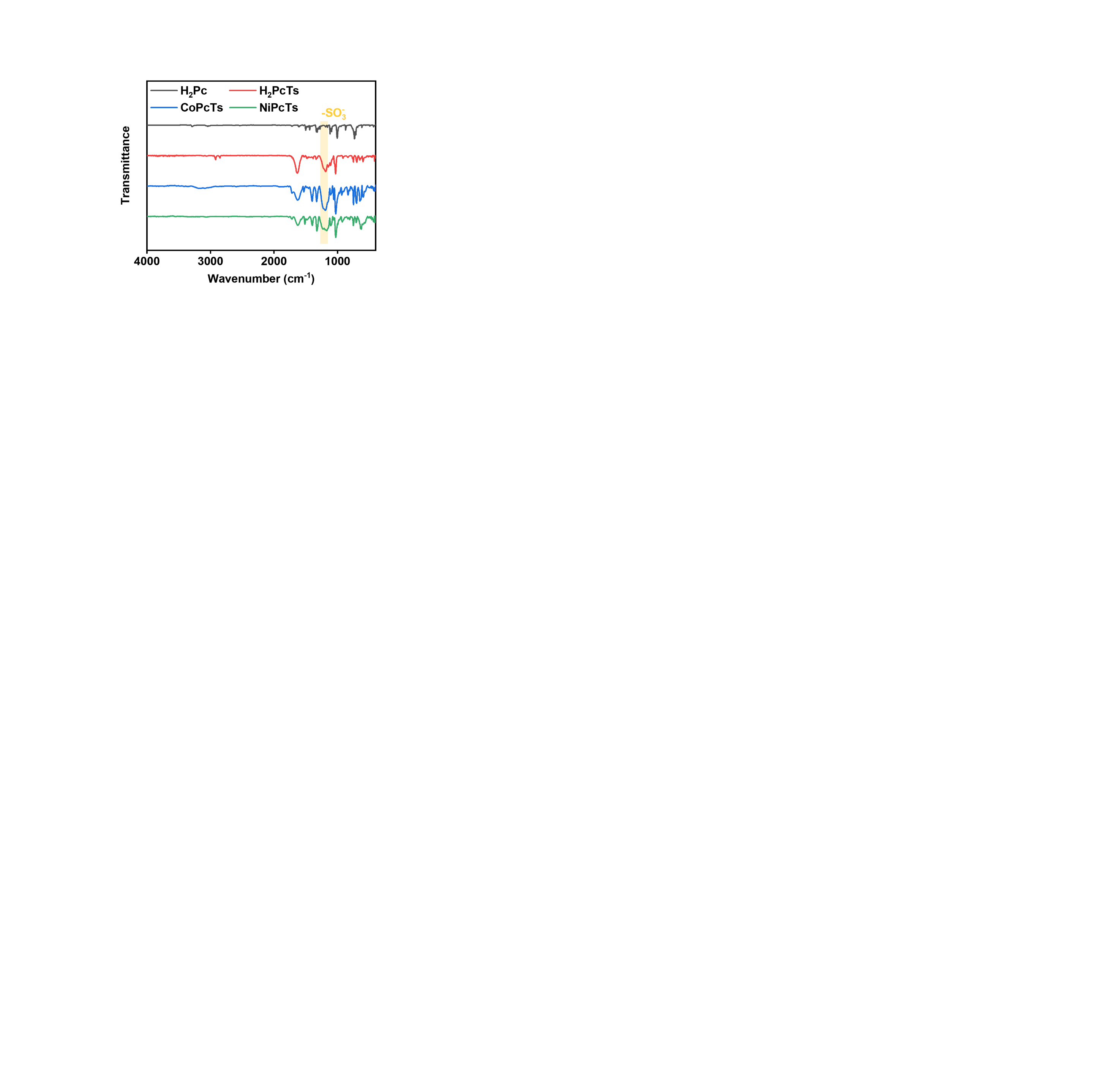


**Figure S2.**  FT-IR spectra of H_2_Pc, H_2_PcTs, CoPcTs and NiPcTs.


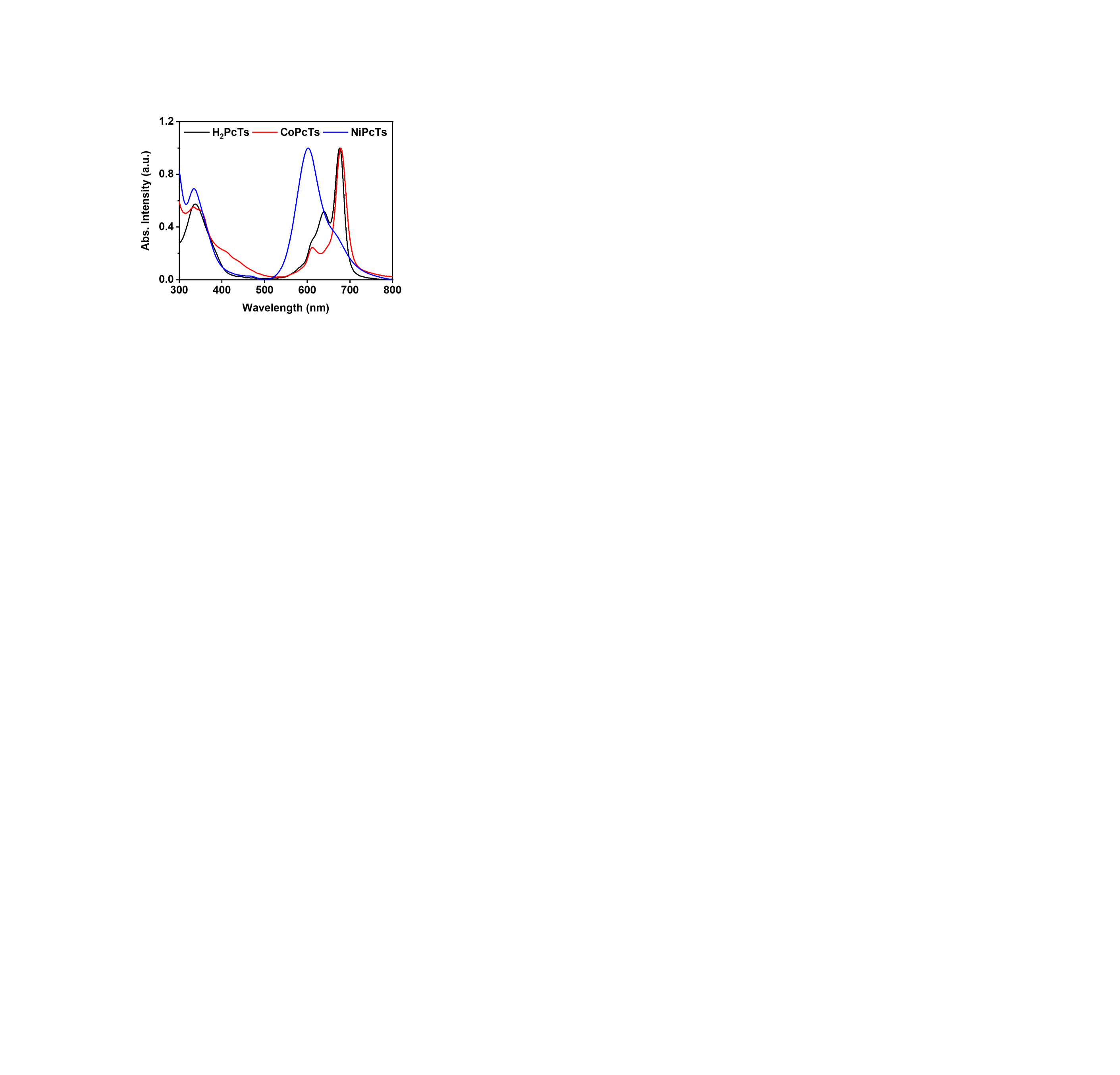


**Figure S3.**  UV-vis spectra of H_2_PcTs, CoPcTs and NiPcTs in ultrapure water (10^-5^ mol L^-1^).


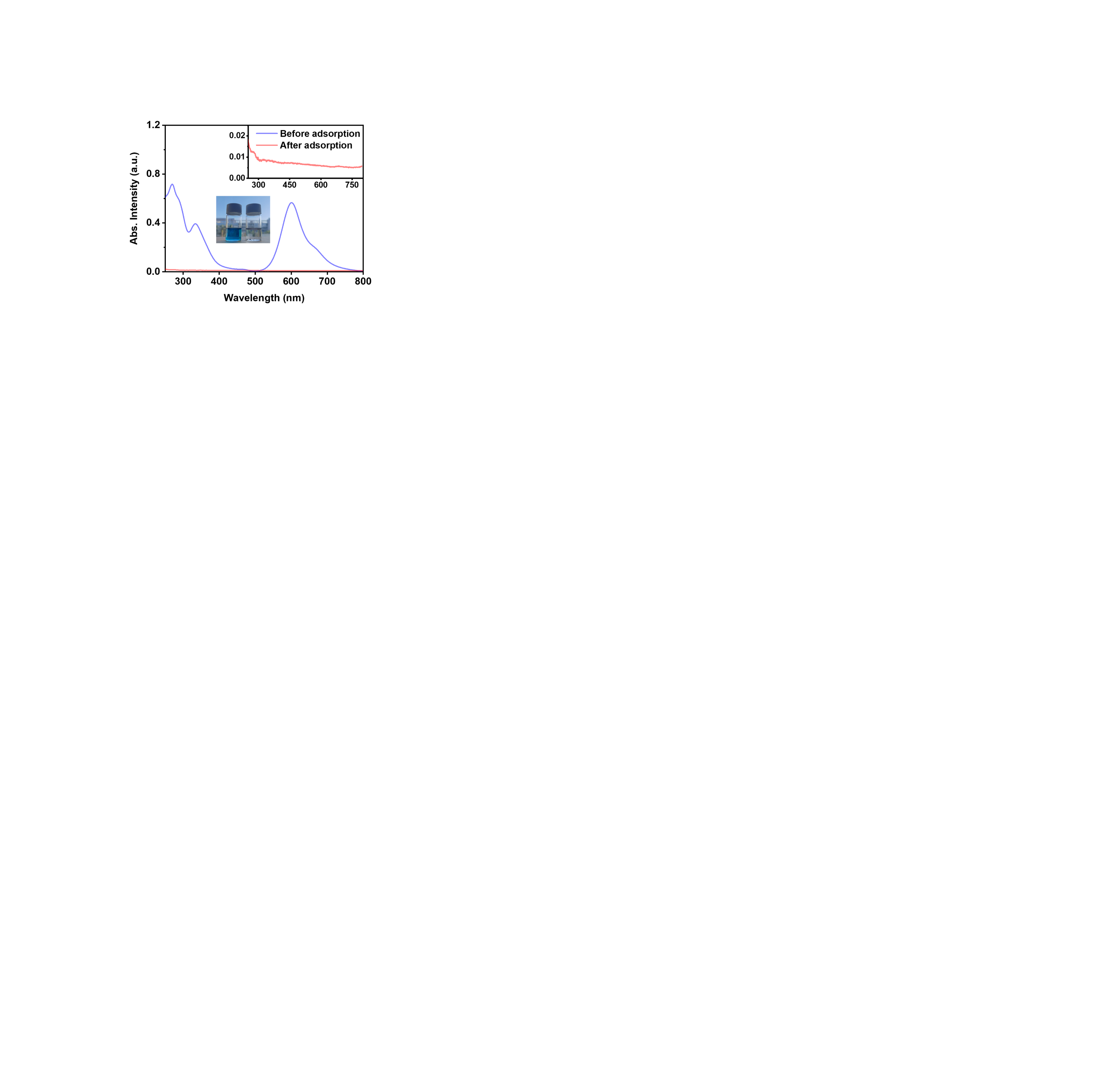


**Figure S4.** UV-vis spectra and corresponding photographs of NiPcTs solution (50 μmol L^-1^) before and after adsorption by CB.


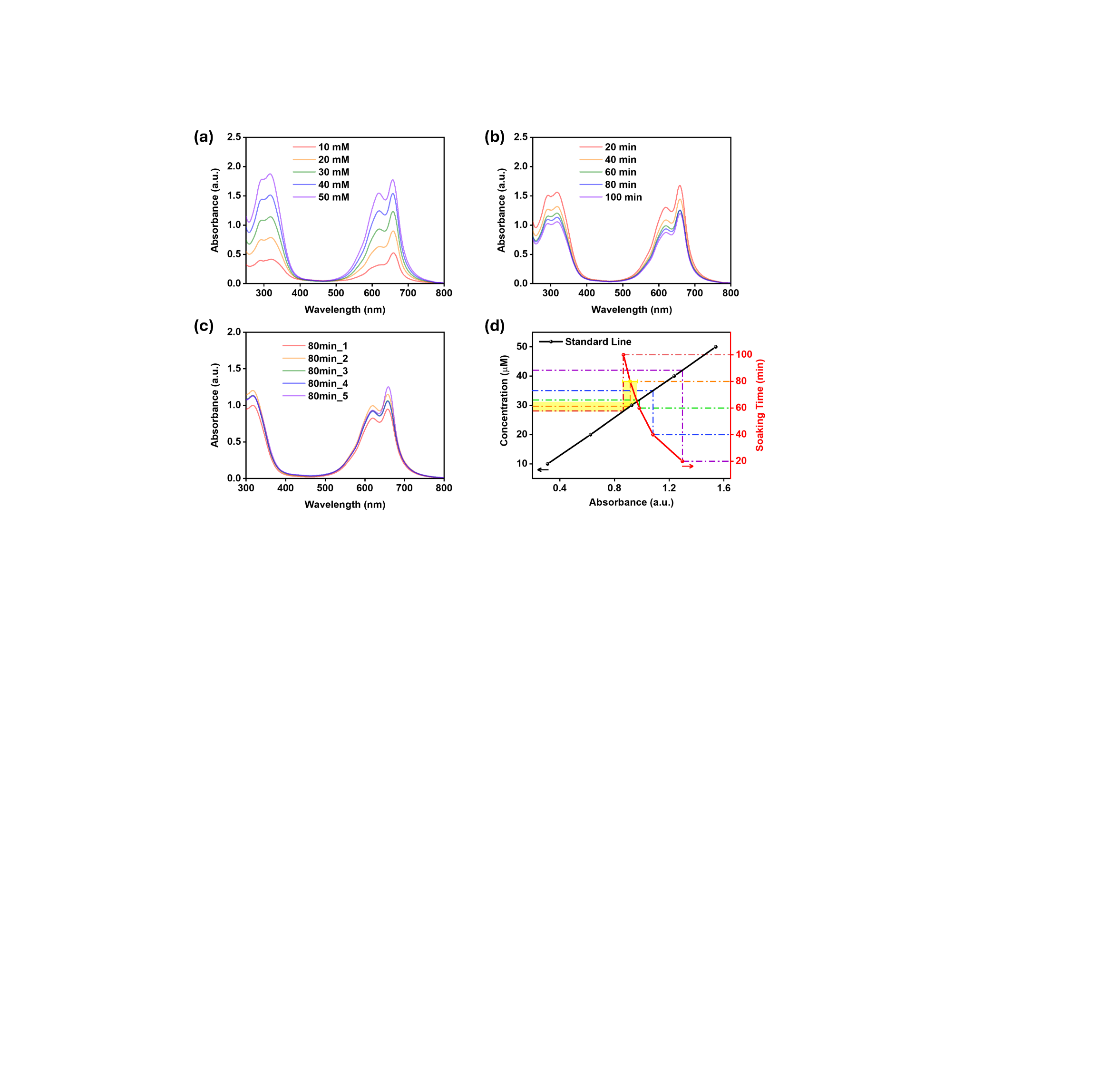


**Figure S5.** (a) UV-vis spectra of CoPcTs solution (in H_2_O) with different concentrations. (b) UV-vis spectra of CoPcTs solution (in H_2_O) after adsorption by bare CB electrodes (400 μg CB coated onto the surface of 1×1 cm^2^ carbon paper) with different soaking time. (c) UV-vis spectra of CoPcTs solution (in H_2_O) after 80-min adsorption by bare CB electrodes. The minor variations in absorption intensity are likely due to slight differences in the exposed CB surface area among individual electrodes. (d) The standard curve (black line) of CoPcTs solution with different concentrations and the relationship between CoPcTs adsorption amount and soaking time.


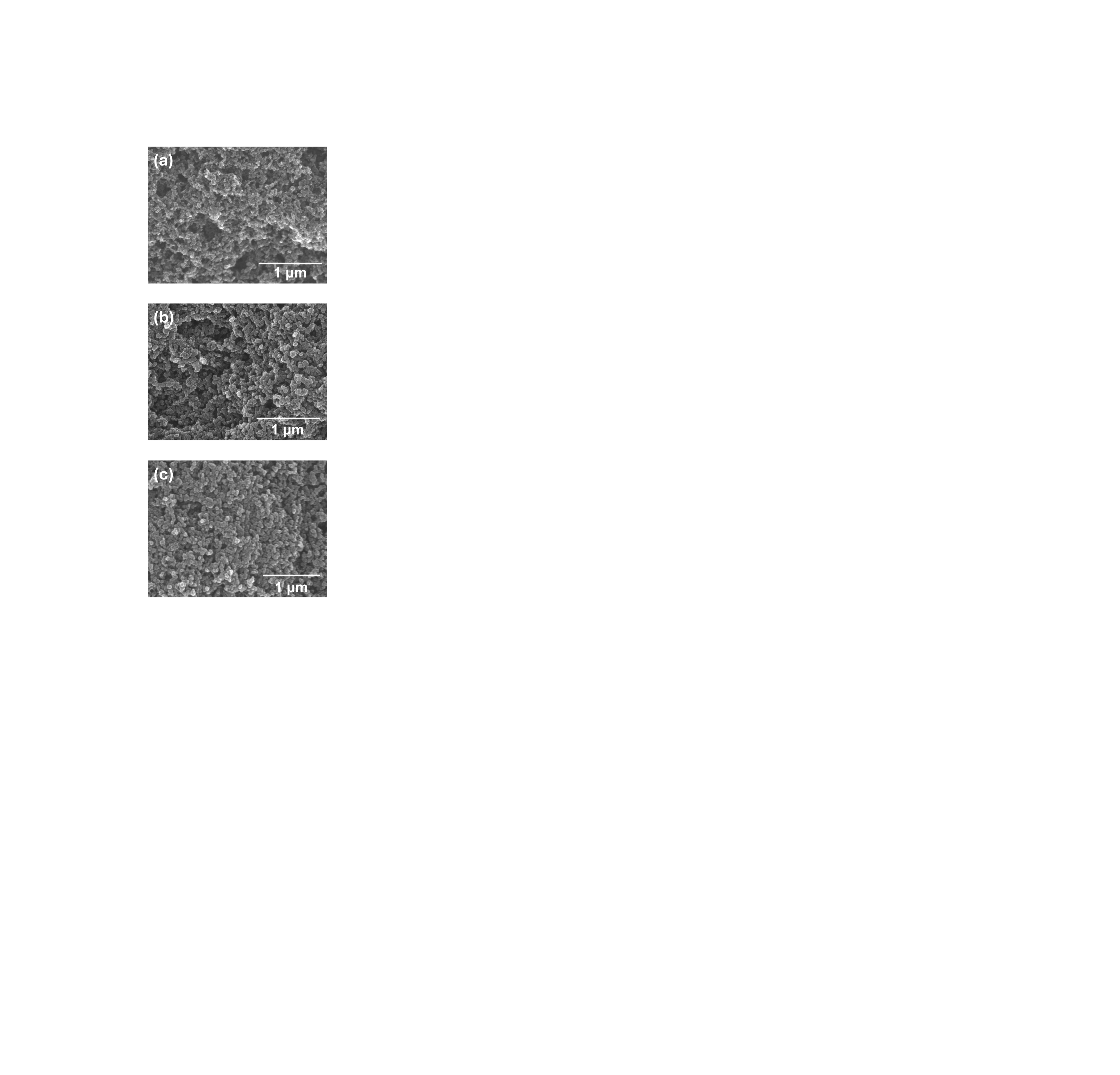


**Figure S6.** SEM images of (a) bare CB electrode, (b) CoPcTs/CB electrode and (c) NiPcTs/CB electrode (soaking time: 80 min) before electrocatalysis.


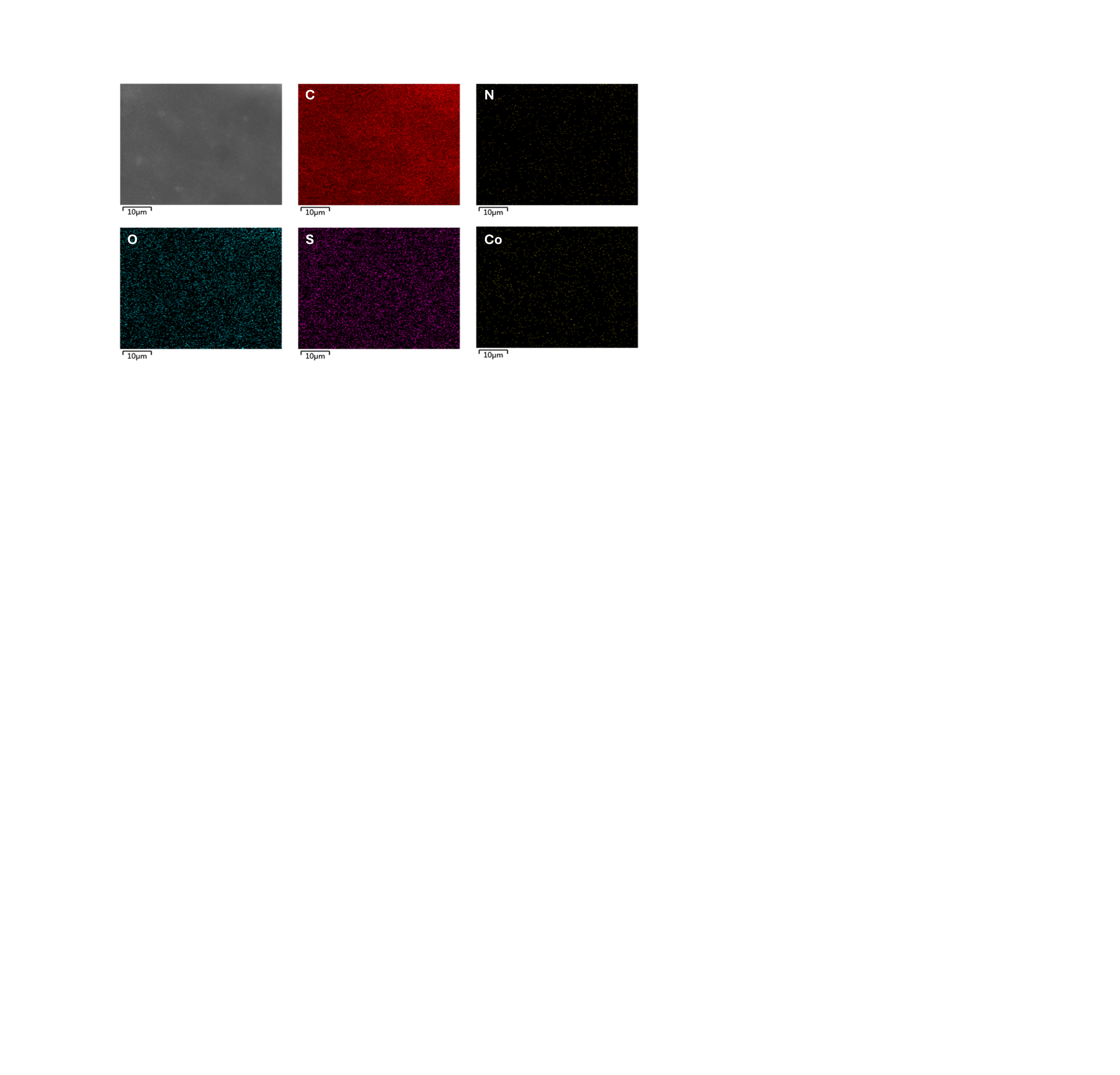


**Figure S7.** SEM image and EDS mapping (including C, N, O, S and Co elements) of CoPcTs/CB electrode (soaking time: 80 min) before electrocatalysis.


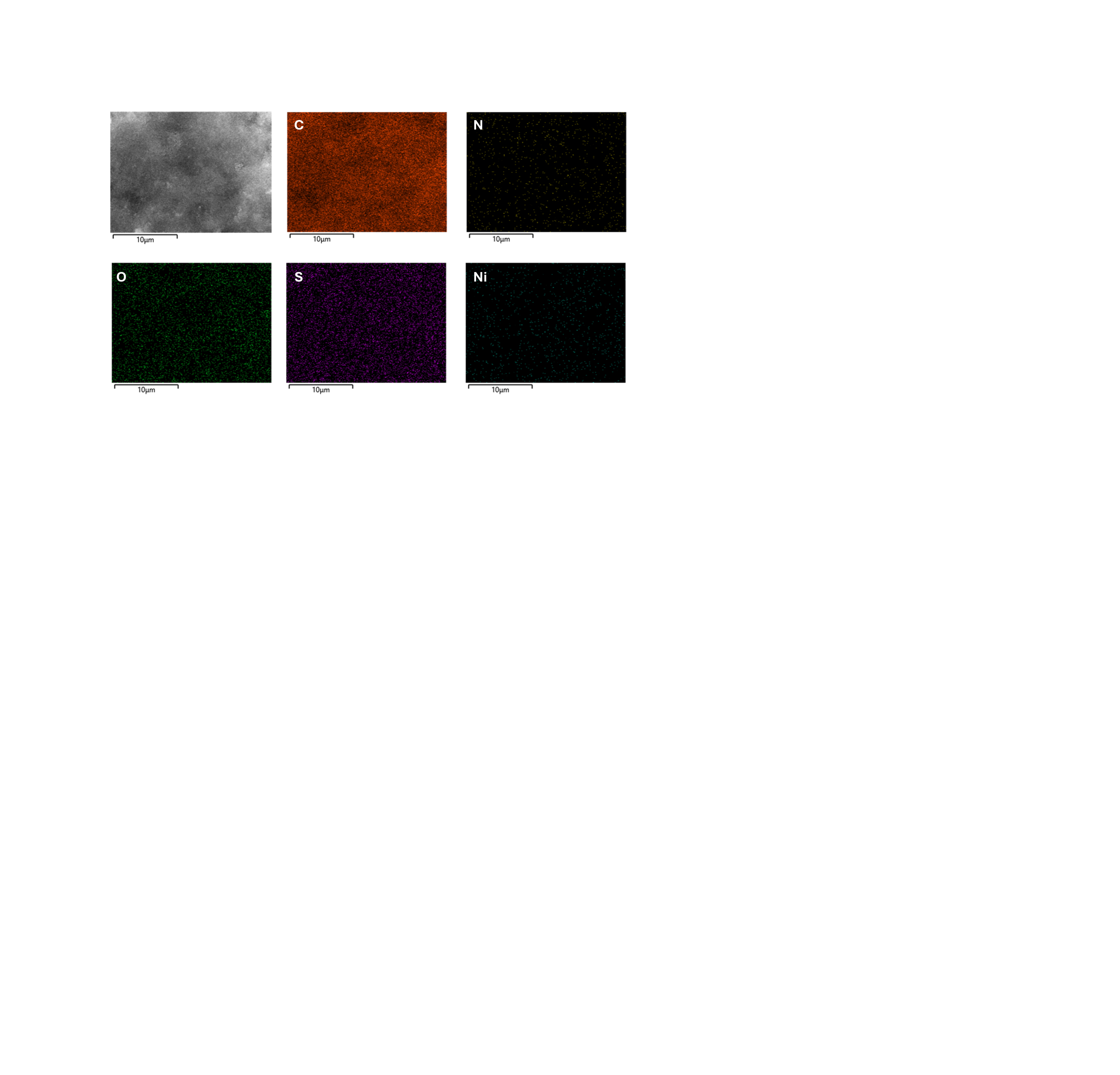


**Figure S8.** SEM image and EDS mapping (including C, N, O, S and Ni elements) of NiPcTs/CB electrode before electrocatalysis.


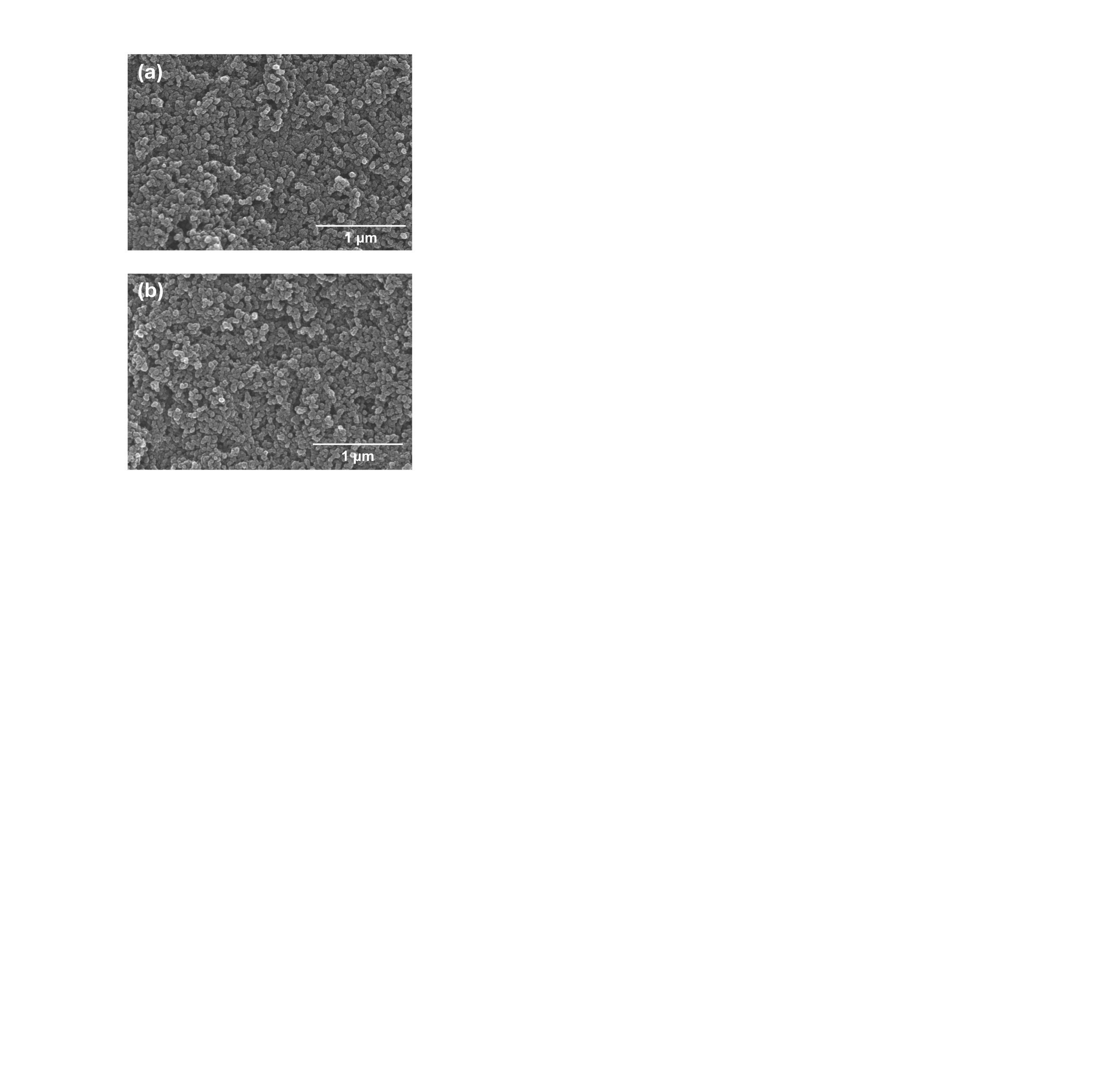


**Figure S9.**  SEM image of (a) CoPcTs/CB electrode and (b) NiPcTs/CB electrode (soaking time: 720 min) before electrocatalysis.


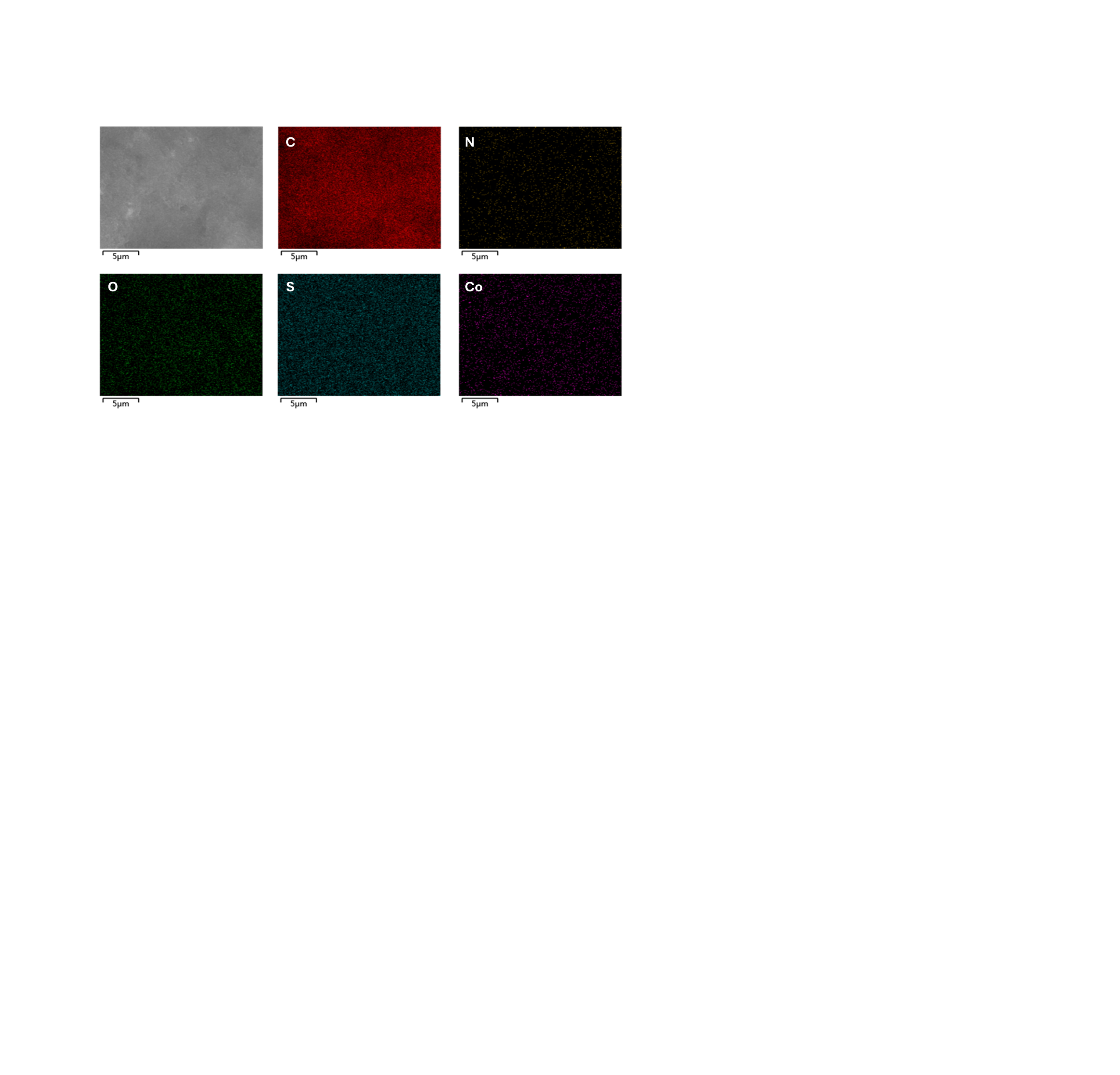


**Figure S10.** SEM image and EDS mapping (including C, N, O, S and Co elements) of CoPcTs/CB electrode (soaking time: 720 min) before electrocatalysis.


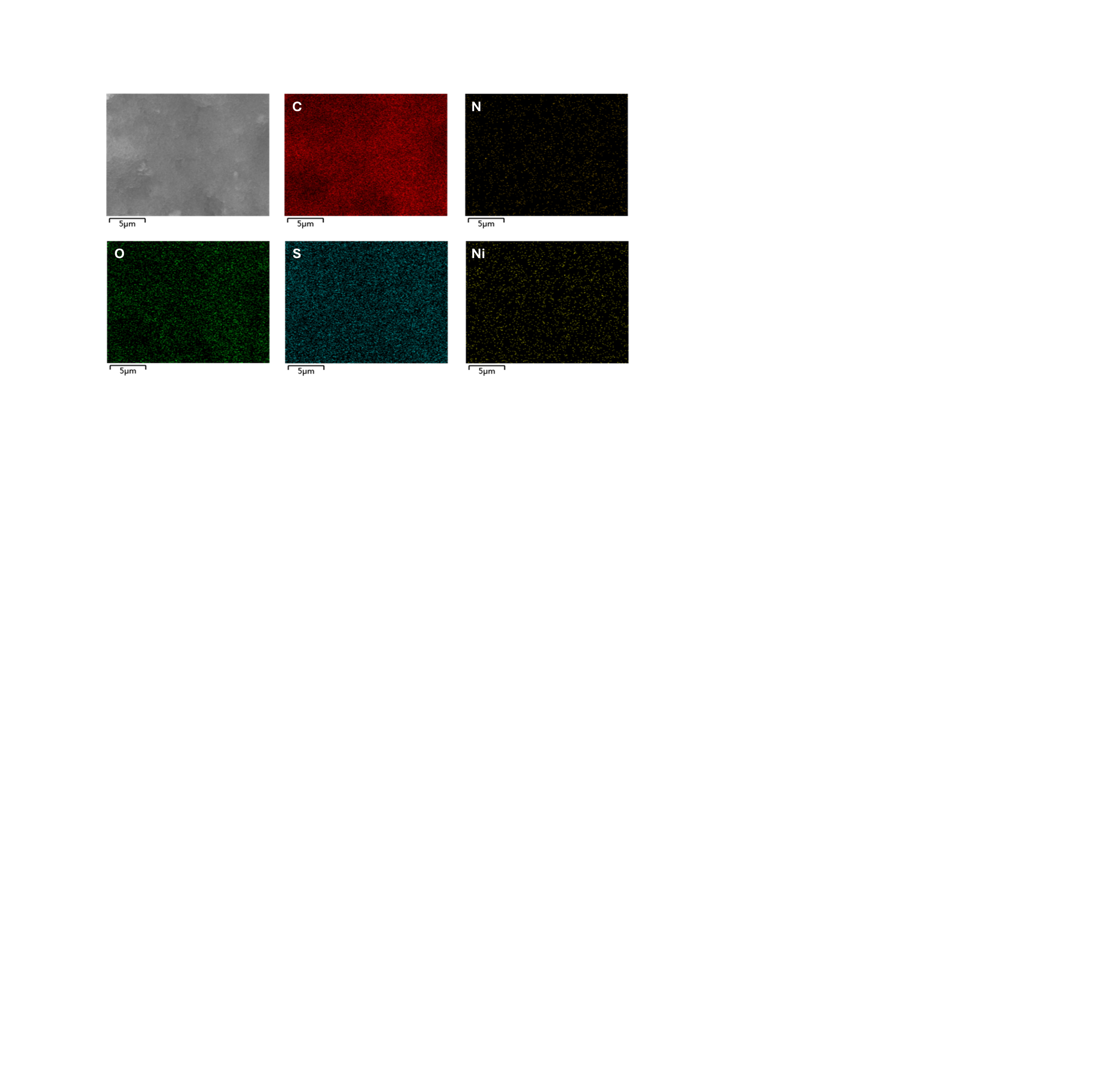


**Figure S11.** SEM image and EDS mapping (including C, N, O, S and Ni elements) of NiPcTs/CB electrode (soaking time: 720 min) before electrocatalysis.


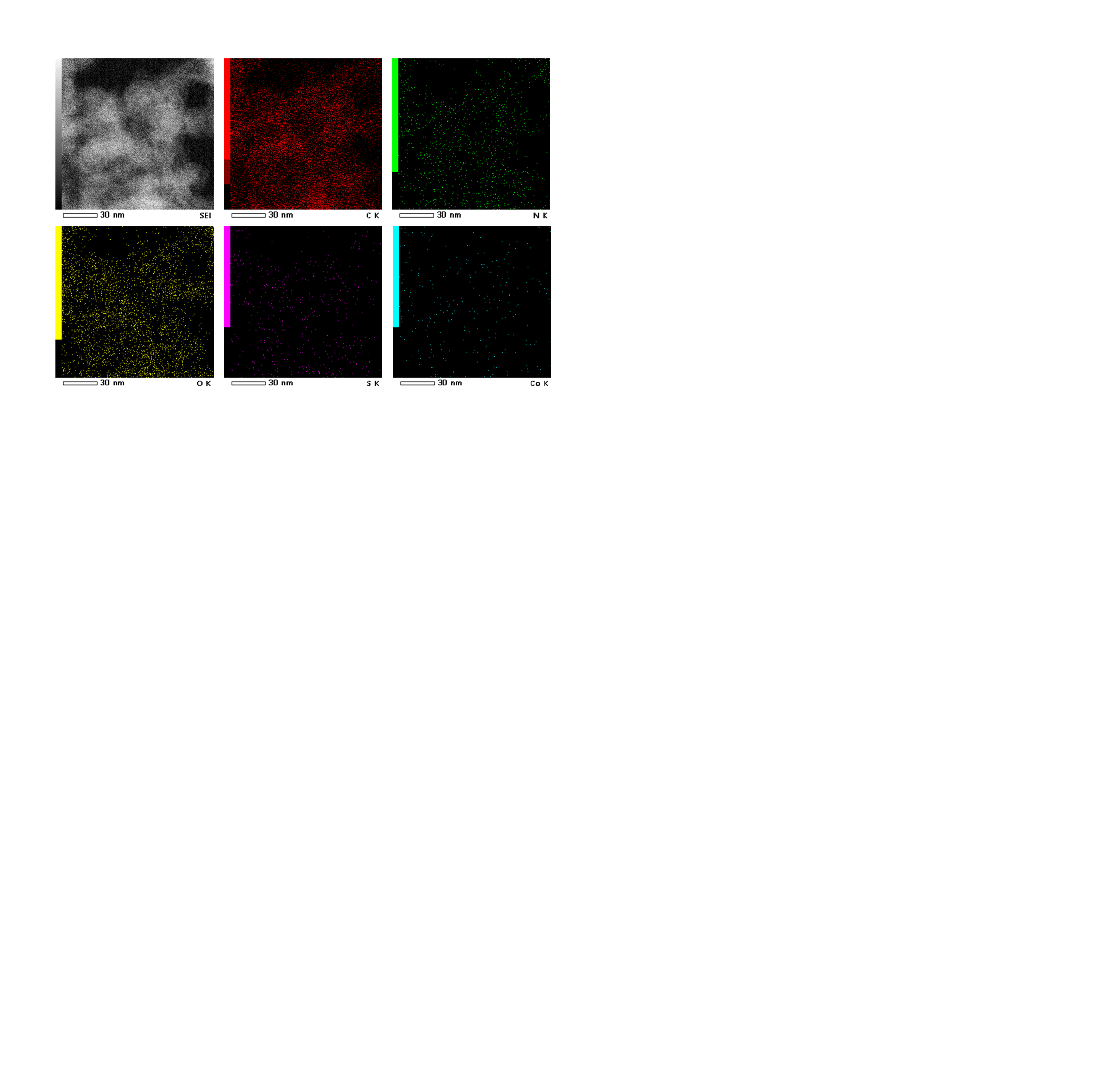


**Figure S12.** STEM-HAADF image and EDS-mapping (including C, N, O, S and Co elements) of CoPcTs/CB.


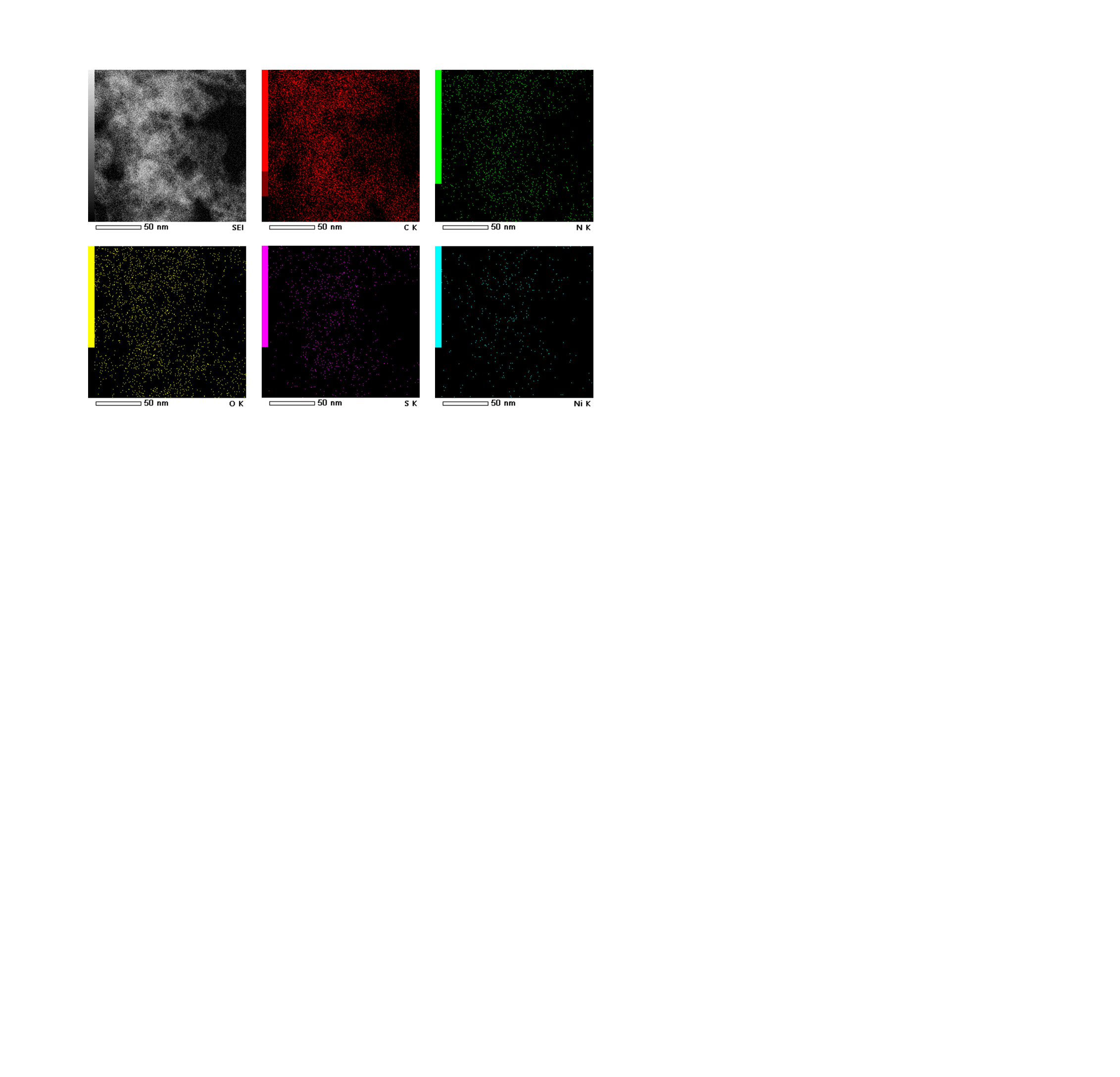


**Figure S13.** STEM-HAADF image and EDS-mapping (including C, N, O, S and Co elements) of NiPcTs/CB.


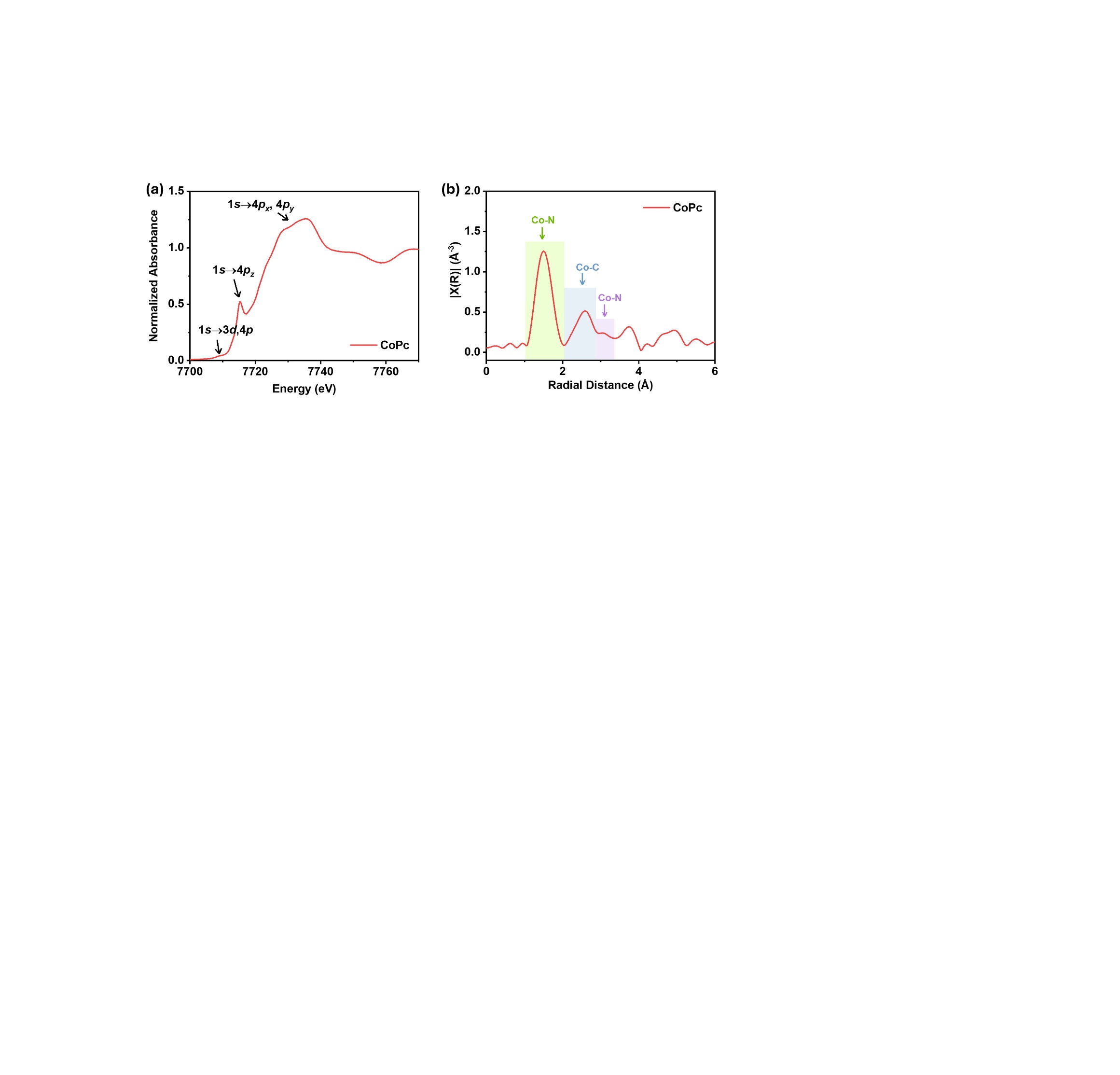


**Figure S14.** (a) Co K-edge XANES spectra and (b) EXAFS spectra in R-space of CoPc sample.


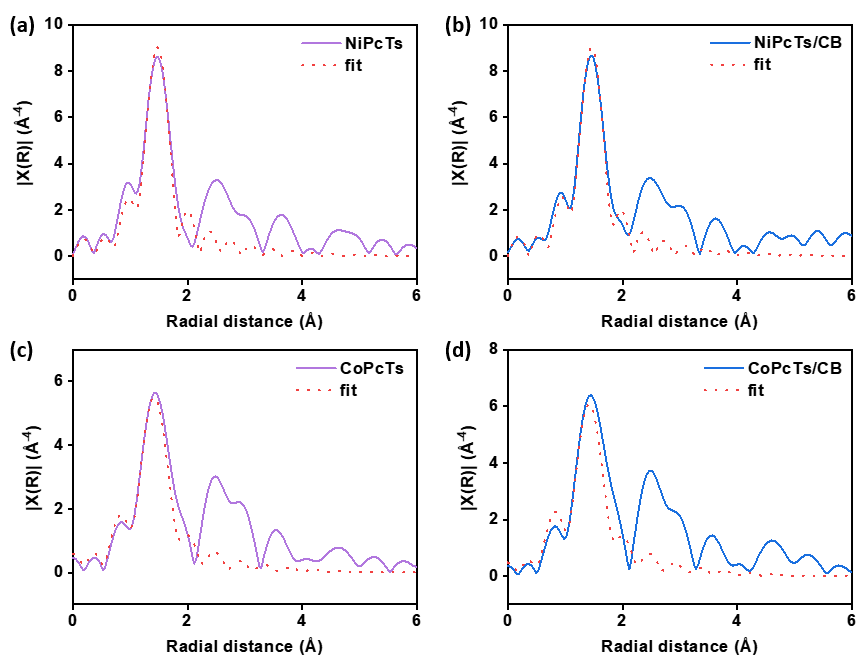


**Figure S15.** The fitted r-space data of (a) NiPcTs, (b) NiPcTs/CB, (c) CoPcTs and (d) CoPcTs/CB.


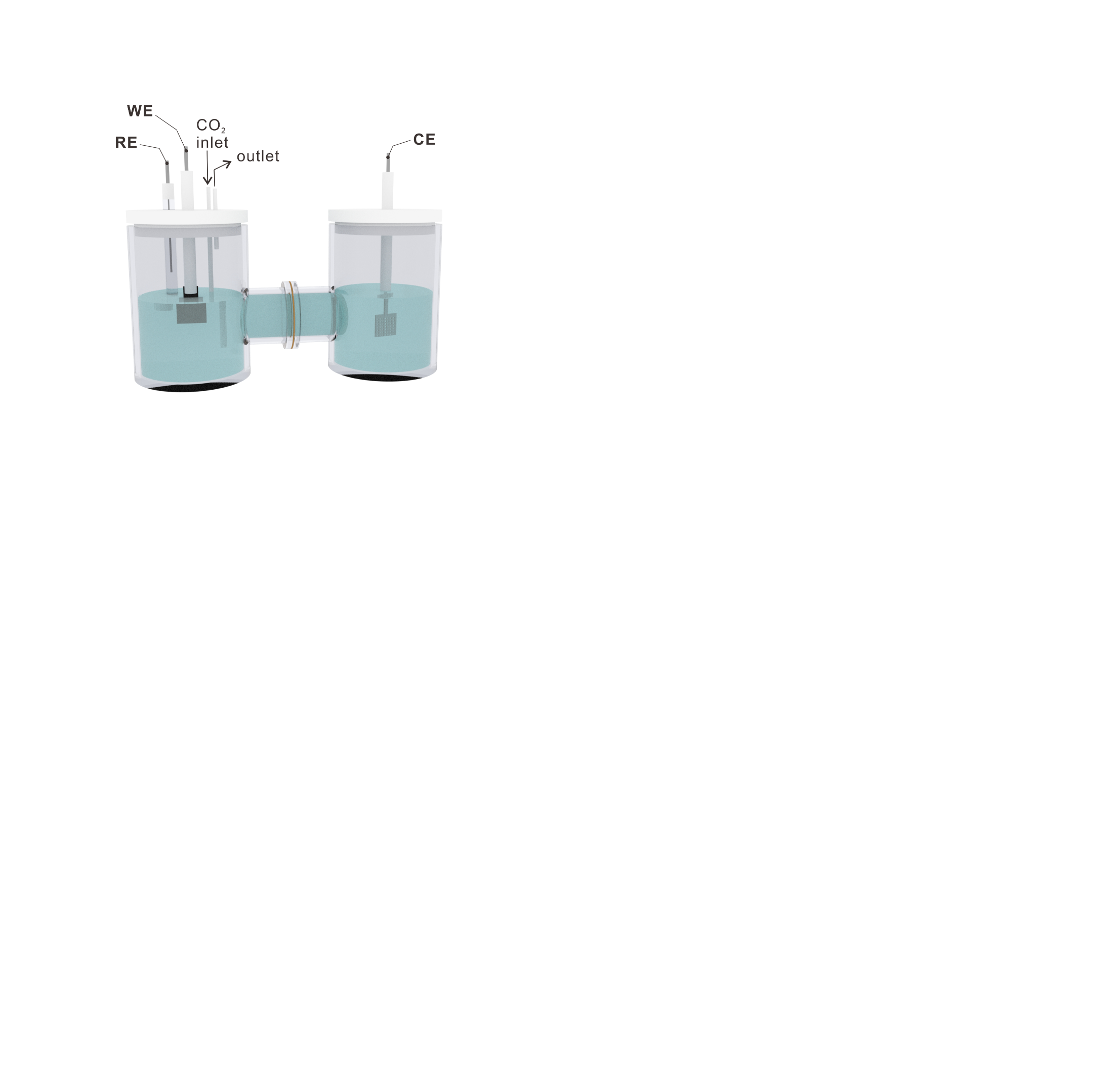


**Figure S16.** The schematic diagram of H-cell. WE = working electrode, RE = reference electrode, CE = counter electrode.


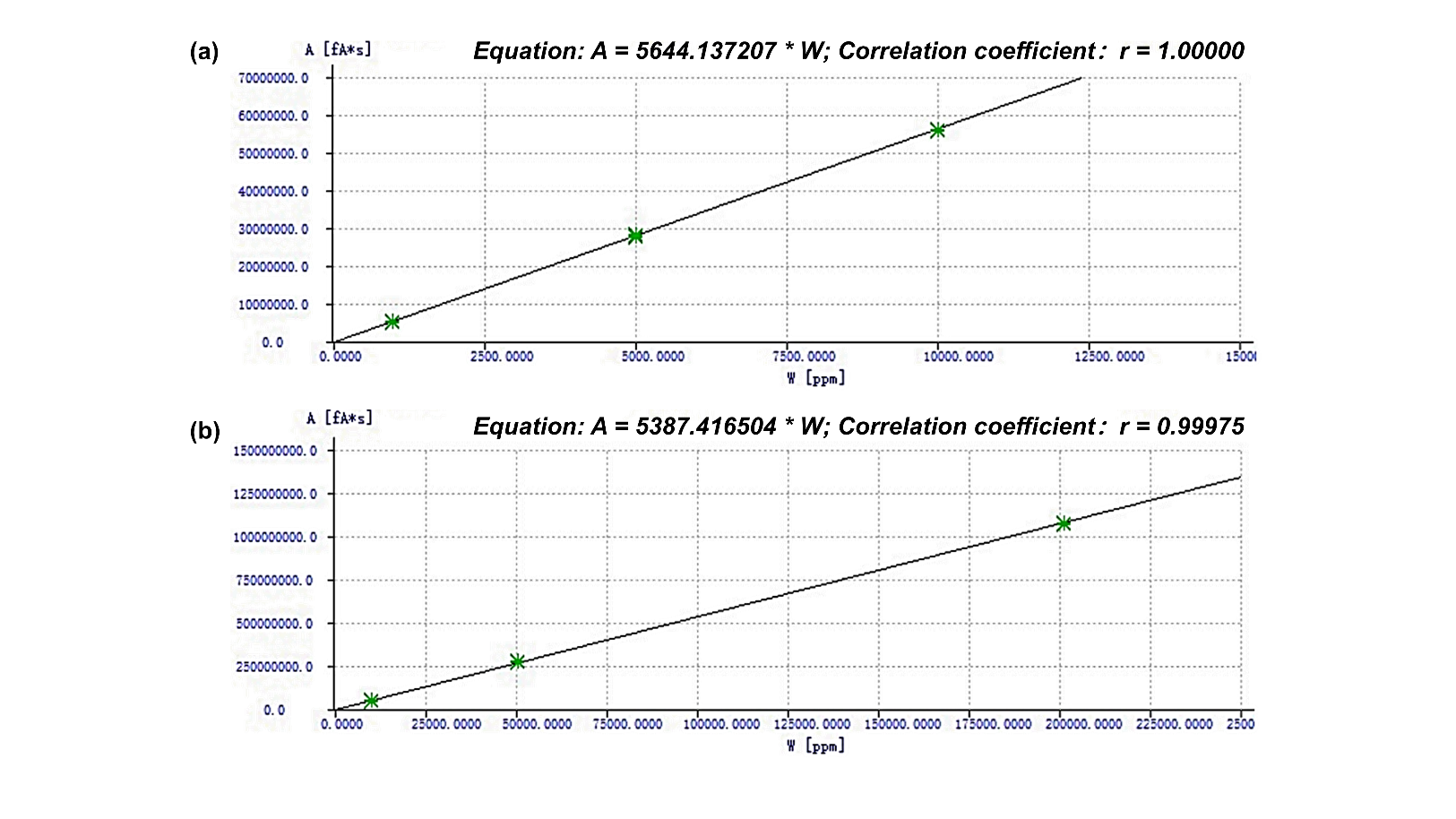


**Figure S17.** (a) Standard calibration curve for CO in the low concentration range (0~10000 ppm), described by the equation *A* = 5644.137207 * *W* (*r* = 1.00000); (b) Standard calibration curve for CO in the high concentration range (10000~200000 ppm), described by *A* = 5387.416504 * *W* (*r* = 0.99975). *A* refers to the integrated area, *W* to the CO concentration, and *r* to the correlation coefficient.


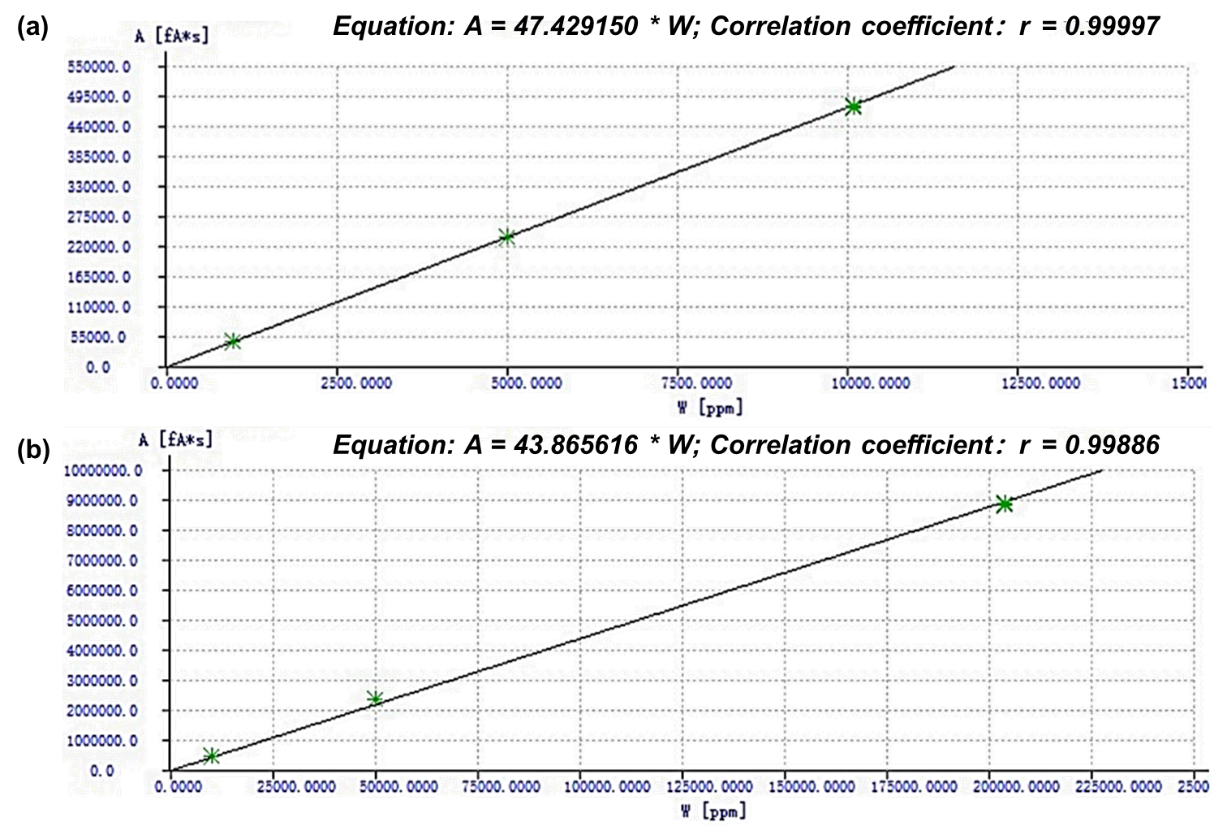


**Figure S18.** (a) Standard calibration curve for H_2_ in the low concentration range (0~10000 ppm), described by the equation *A* = 47.429150 * *W* (*r* = 0.99997); (b) Standard calibration curve for CO in the high concentration range (10000~200000 ppm), described by *A* = 43.865616 * *W* (*r* = 0.99886). *A* refers to the integrated area, *W* to the H_2_ concentration, and *r* to the correlation coefficient.


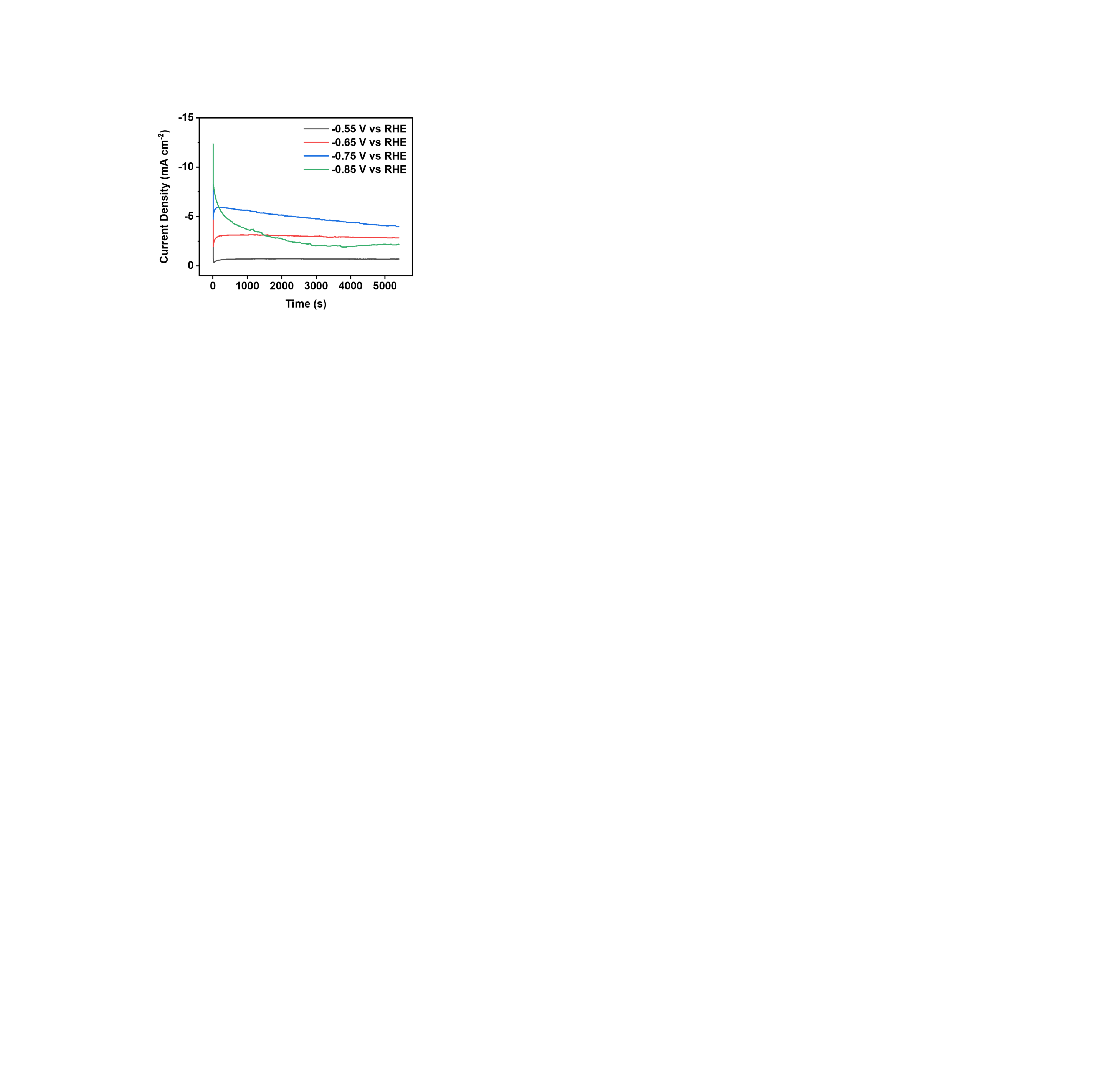


**Figure S19.** The current density–time relationship of NiPcTs/CB at various applied potentials (−0.55 ~ −0.85 V vs RHE) in CO_2_-saturated 0.5 M KHCO_3_ aqueous solution in H-cell.


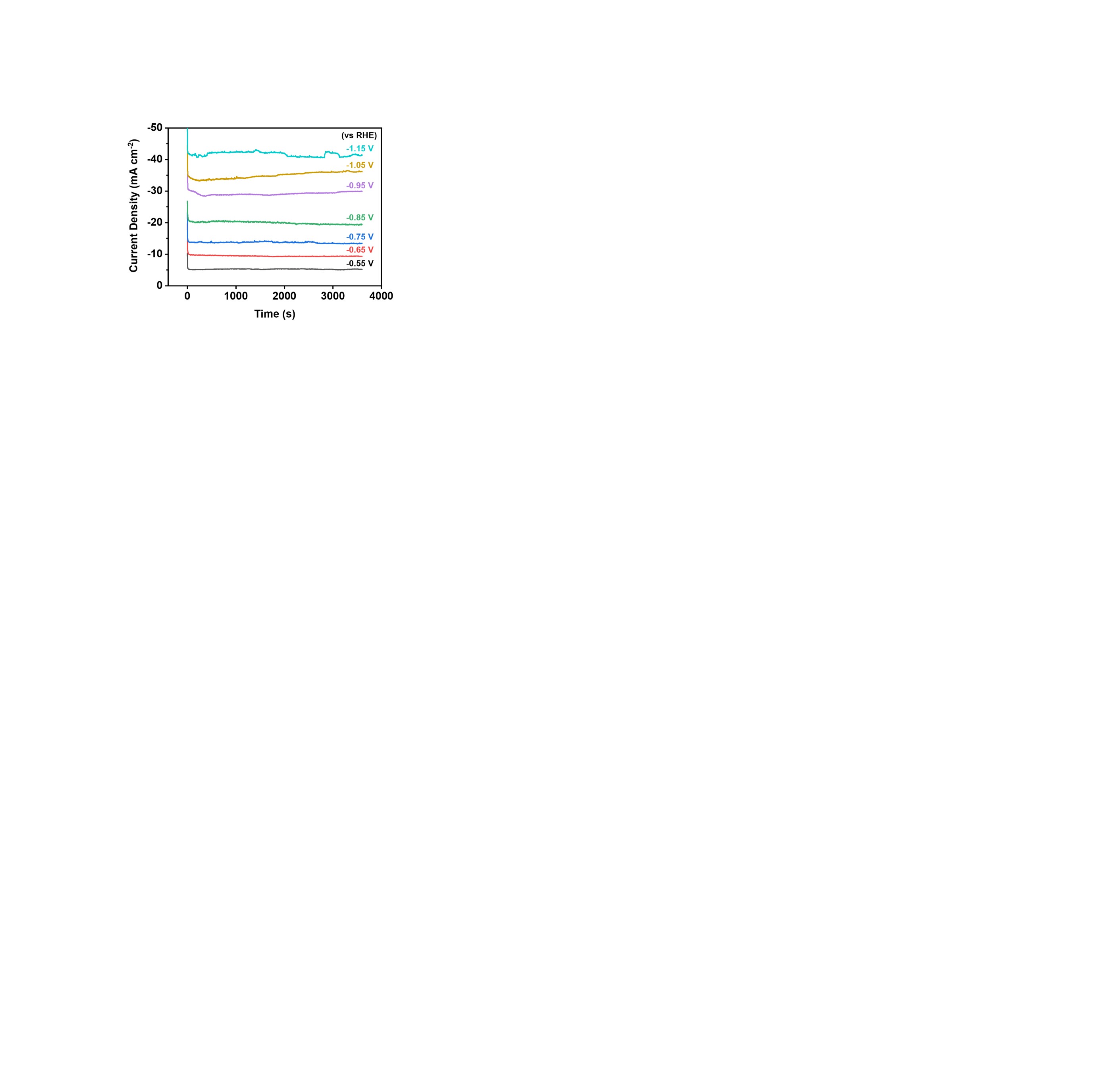


**Figure S20.** The current density–time relationship of CoPcTs/CB at various applied potentials (−0.55 ~ −1.15 V vs RHE) in CO_2_-saturated 0.5 M KHCO_3_ aqueous solution in H-cell.


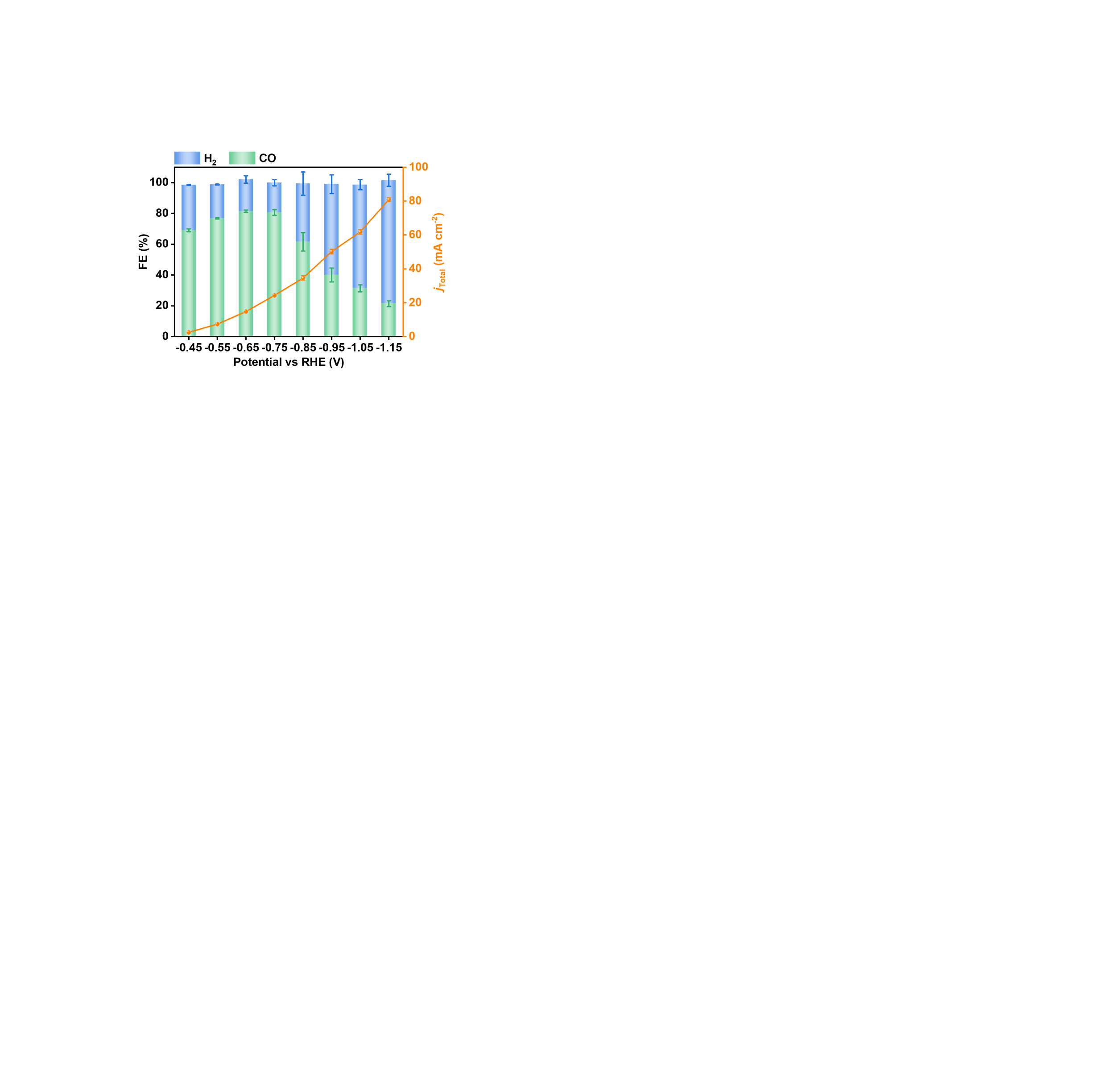


**Figure S21.** FE_CO_, FE_H2_ and current density as a function of the applied potentials for CoPcTs/CB catalysts operated in CO_2_-saturated 0.5 M KHCO_3_ aqueous solution in H-cell. The error bars indicate the among values from three repeated measurements.


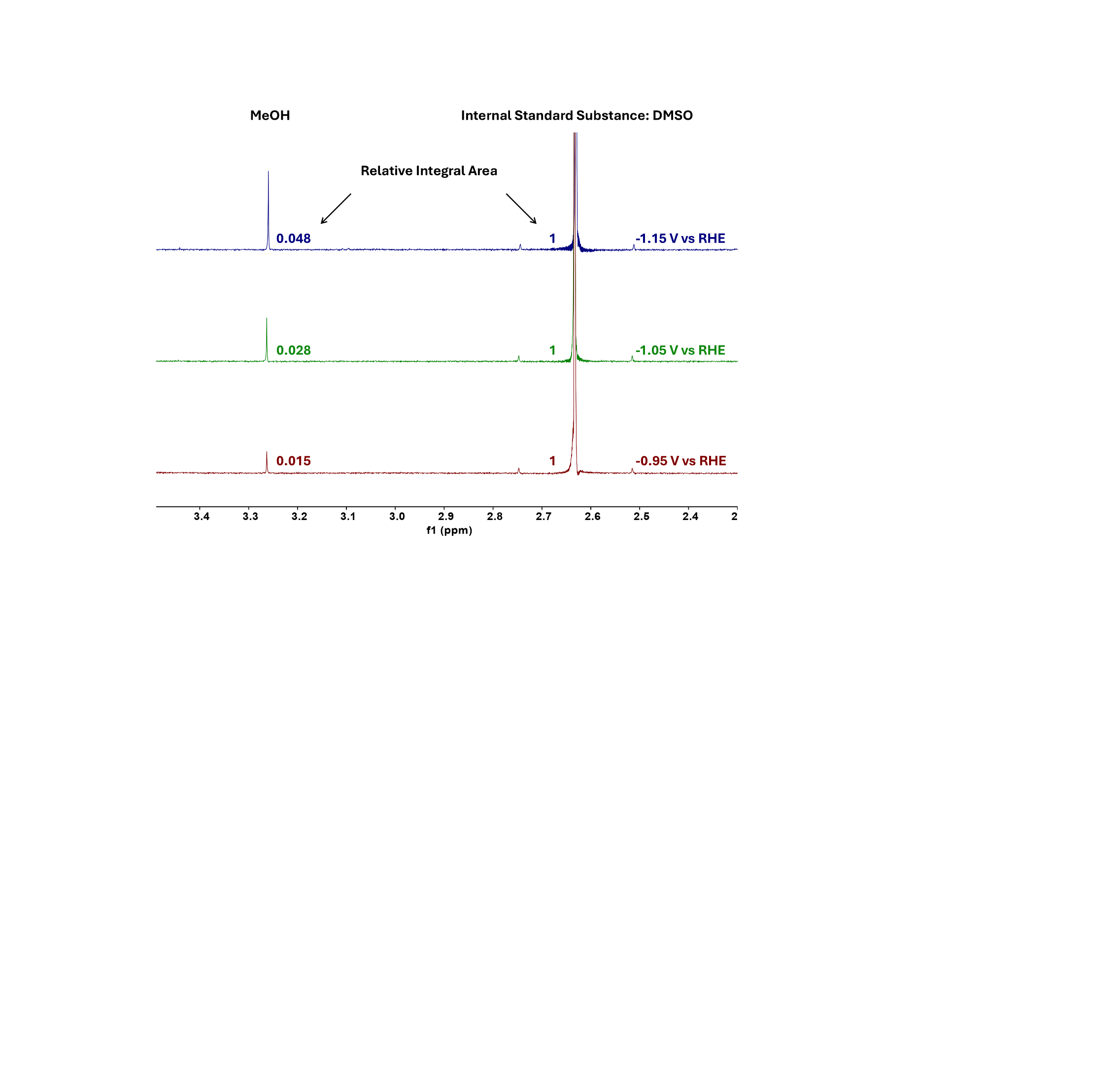


**Figure S22.** The ^1^H NMR spectrum of the electrolyte (CO_2_ saturated 0.5 M KHCO_3_ aqueous solution) in D_2_O after 1-hour electro-catalysis measurements by CoPcTs/CB from potentials at −0.95 to −1.15 V vs RHE.


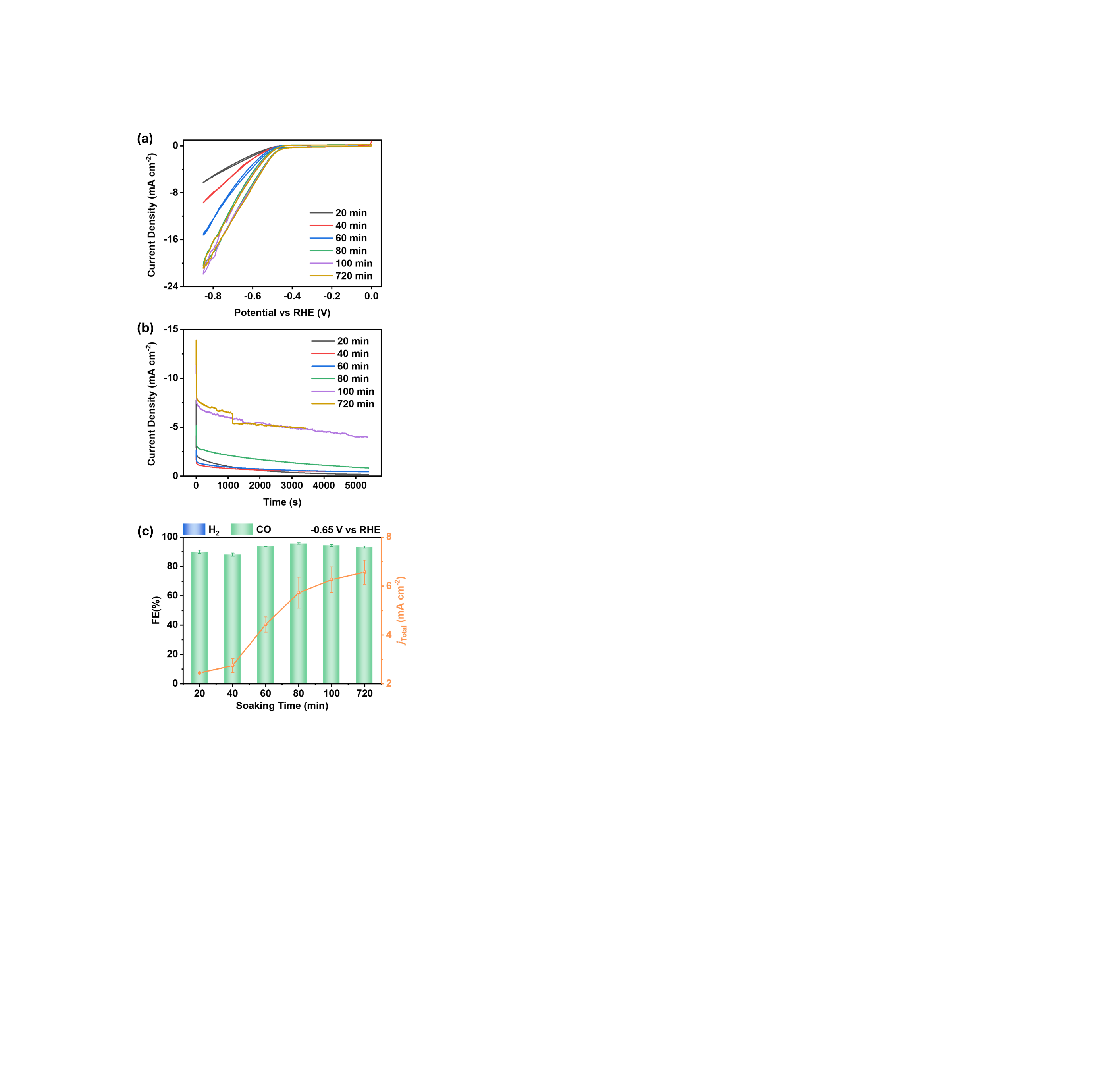


**Figure S23.** (a) Cyclic voltammograms, (b) current density–time relationship and (c) FE_CO_ and FE_H2_ of NiPcTs/CB catalyst in CO_2_RR experiments at –0.65 V vs RHE with different soaking time in H-cell. The error bars indicate the among values from three repeated measurements.


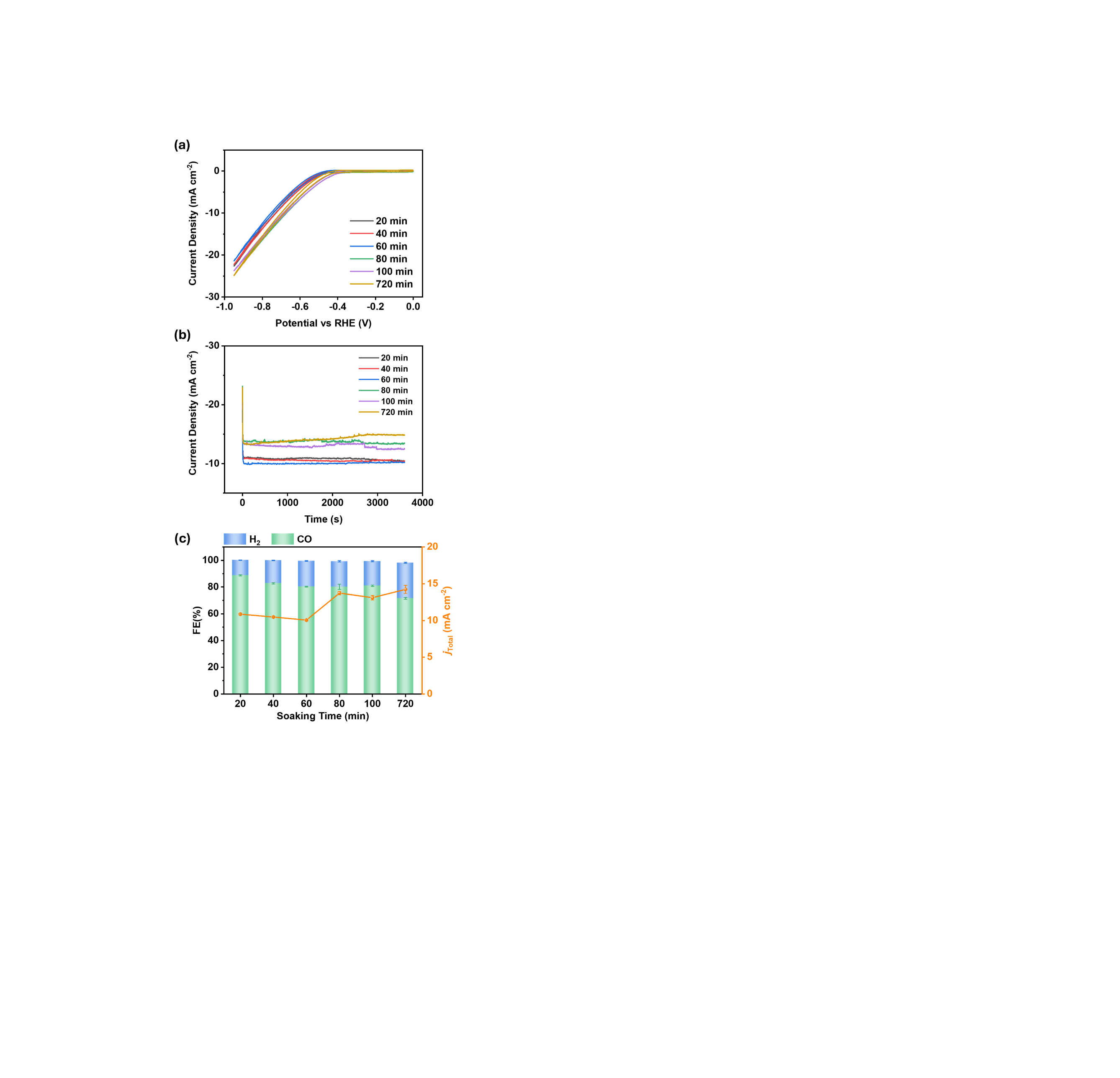


**Figure S24.** (a) Cyclic voltammograms, (b) current density–time relationship and (c) FE_CO_ and FE_H2_ of CoPcTs/CB catalyst in CO_2_RR experiments at –0.75 V vs RHE with different soaking time in H-cell. The error bars indicate the among values from three repeated measurements.


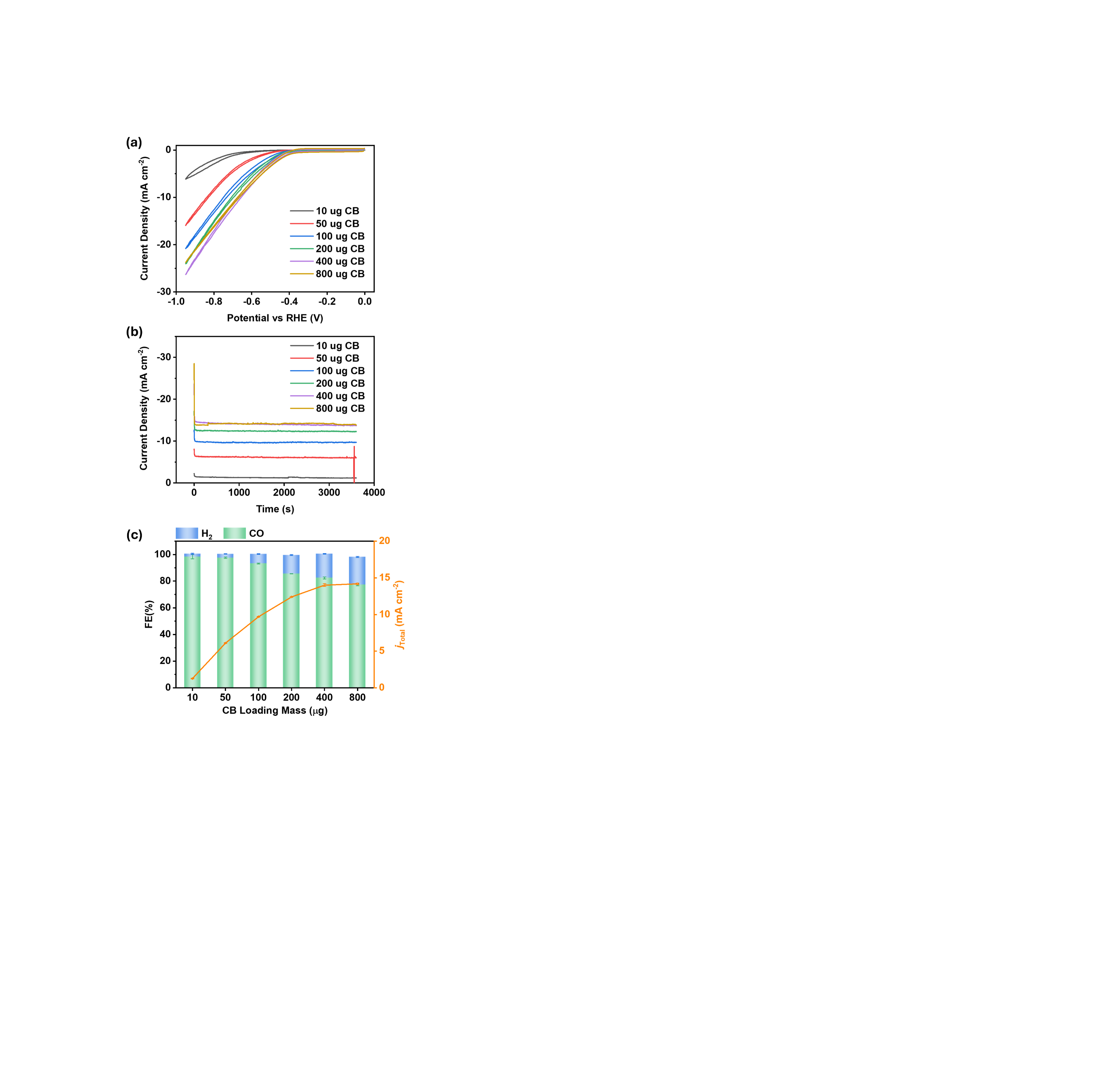


**Figure S25.** (a) CV curves and (b) Current density−time relationship of CoPcTs/CB electrode operated at −0.75 V vs RHE in CO_2_-saturated 0.5 M KHCO_3_ aqueous solution in an H-cell.


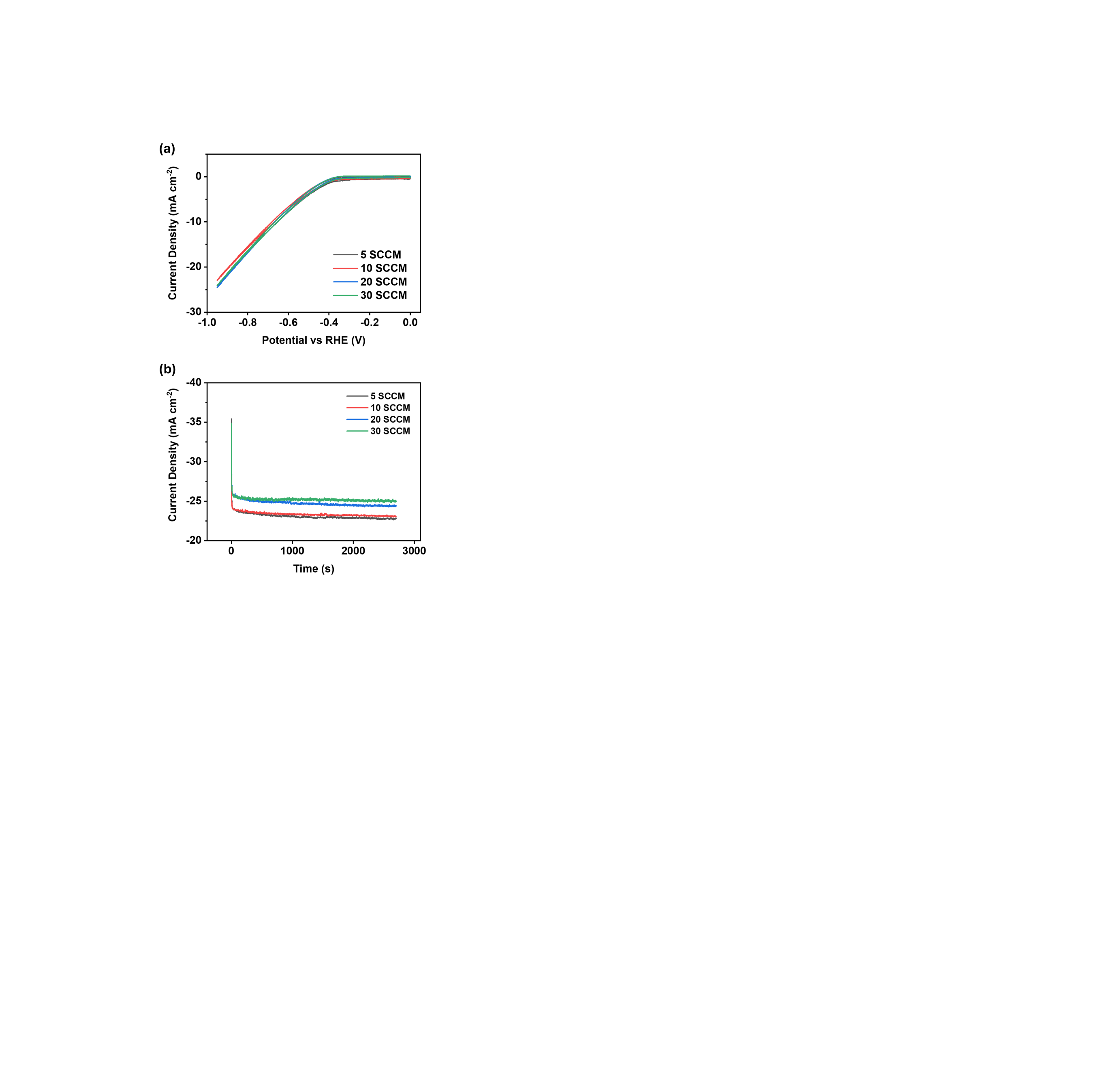


**Figure S26.** (a) CV curves and (b) Current density−time relationship of CoPcTs/CB electrode operated at −0.75 V vs RHE in CO_2_-saturated 0.5 M KHCO_3_ aqueous solution in an H-cell.


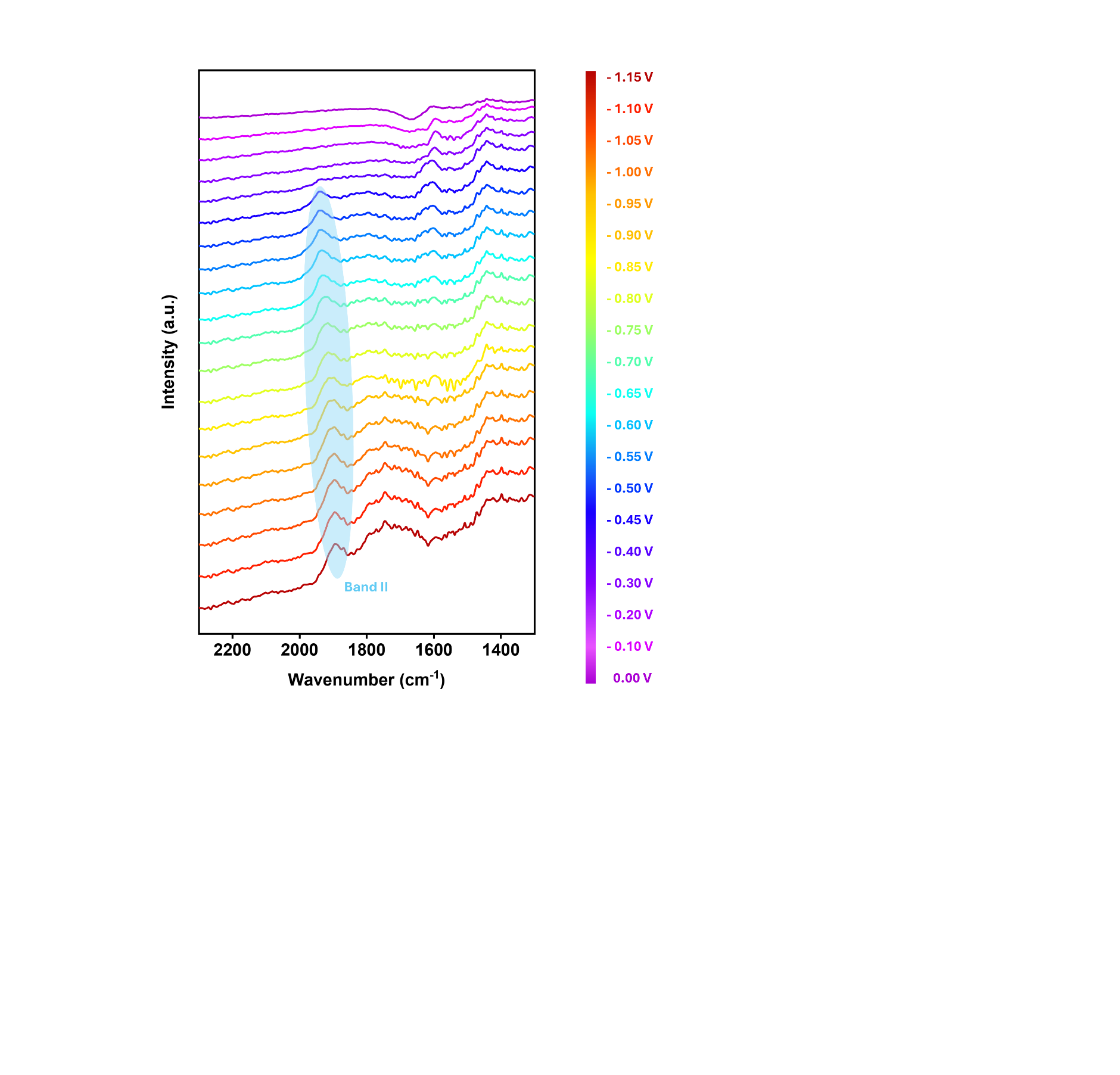


**Figure S27**. ATR-SEIRA spectra of NiPcTs/CB measured at 0 V ~ −1.15 V vs RHE in CO_2_-saturated 0.5 M KHCO_3_ electrolyte. The band in the marked region (1940–1894 cm^−1^) corresponding to linearly adsorbed CO on quasi-flat Ni–N_4_ centers.


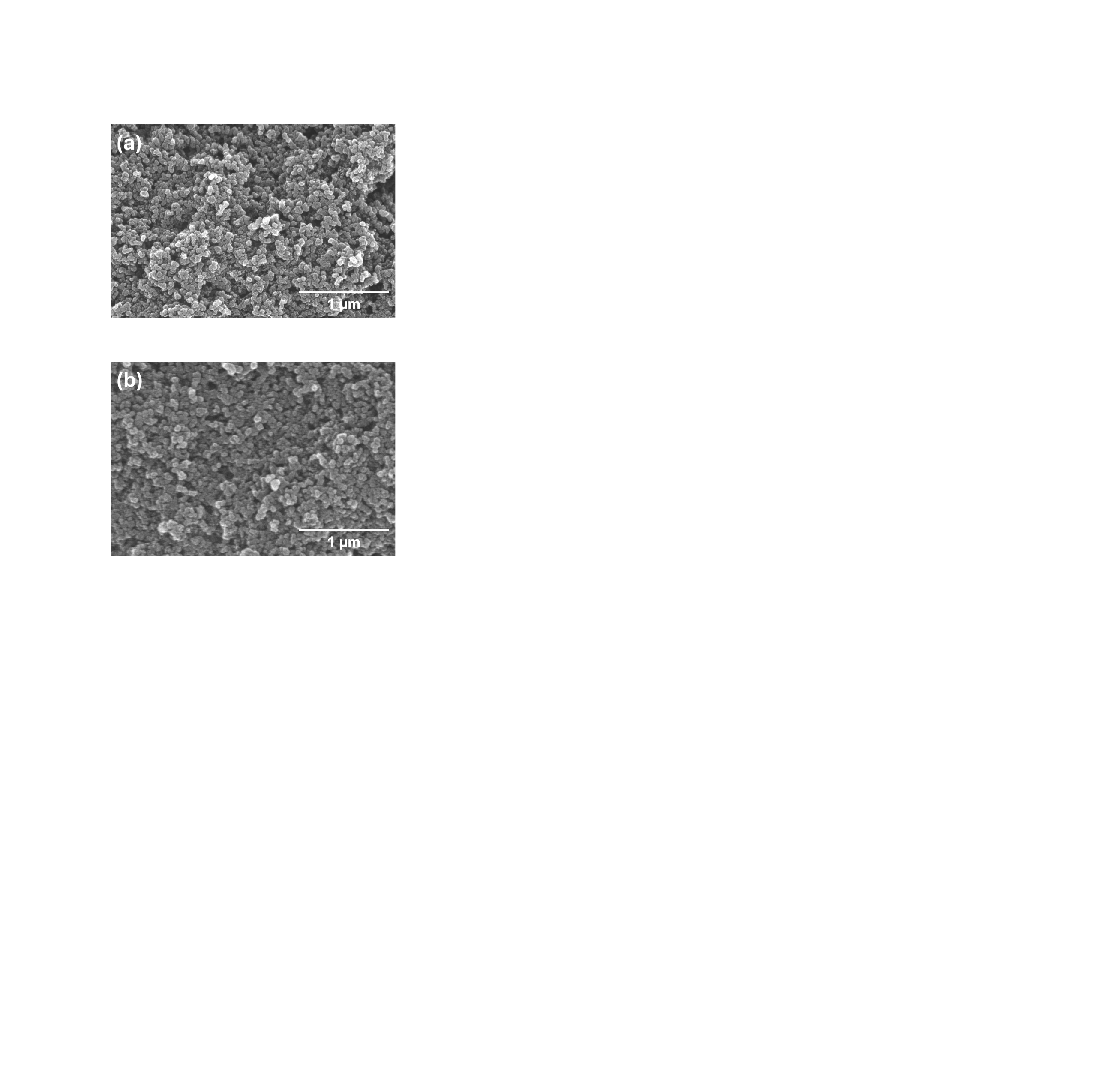


**Figure S28.** SEM images of (a) CoPcTs/CB electrode and (b) NiPcTs/CB electrode (soaking time: 80 min) after 1-hour electrocatalysis at -0.75 V vs RHE in H-cell.


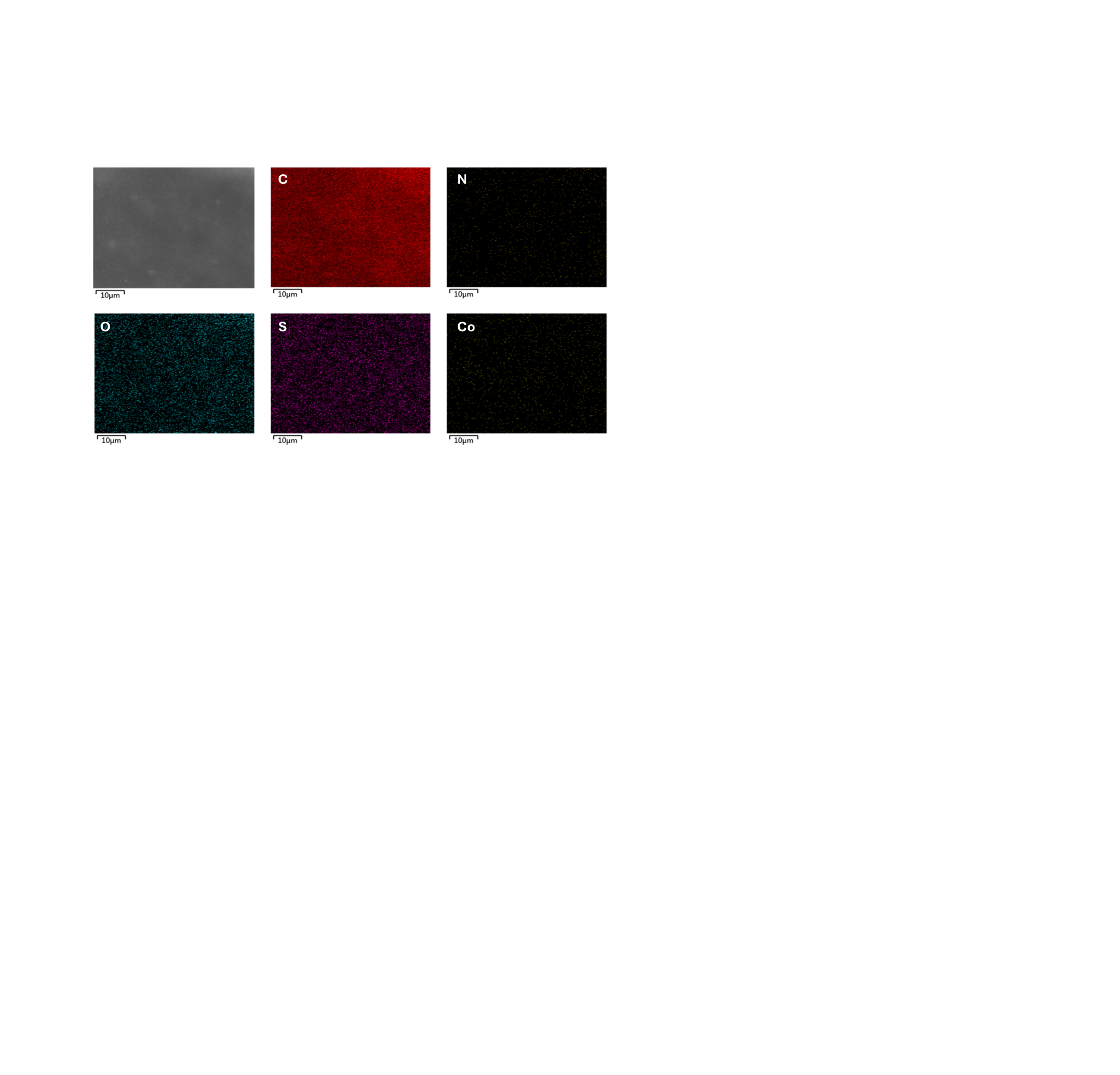


**Figure S29.** SEM image and EDS mapping (including C, N, O, S and Co elements) of CoPcTs/CB electrode (soaking time: 80 min) after 1-hour electrocatalysis at -0.75 V vs RHE in H-cell.


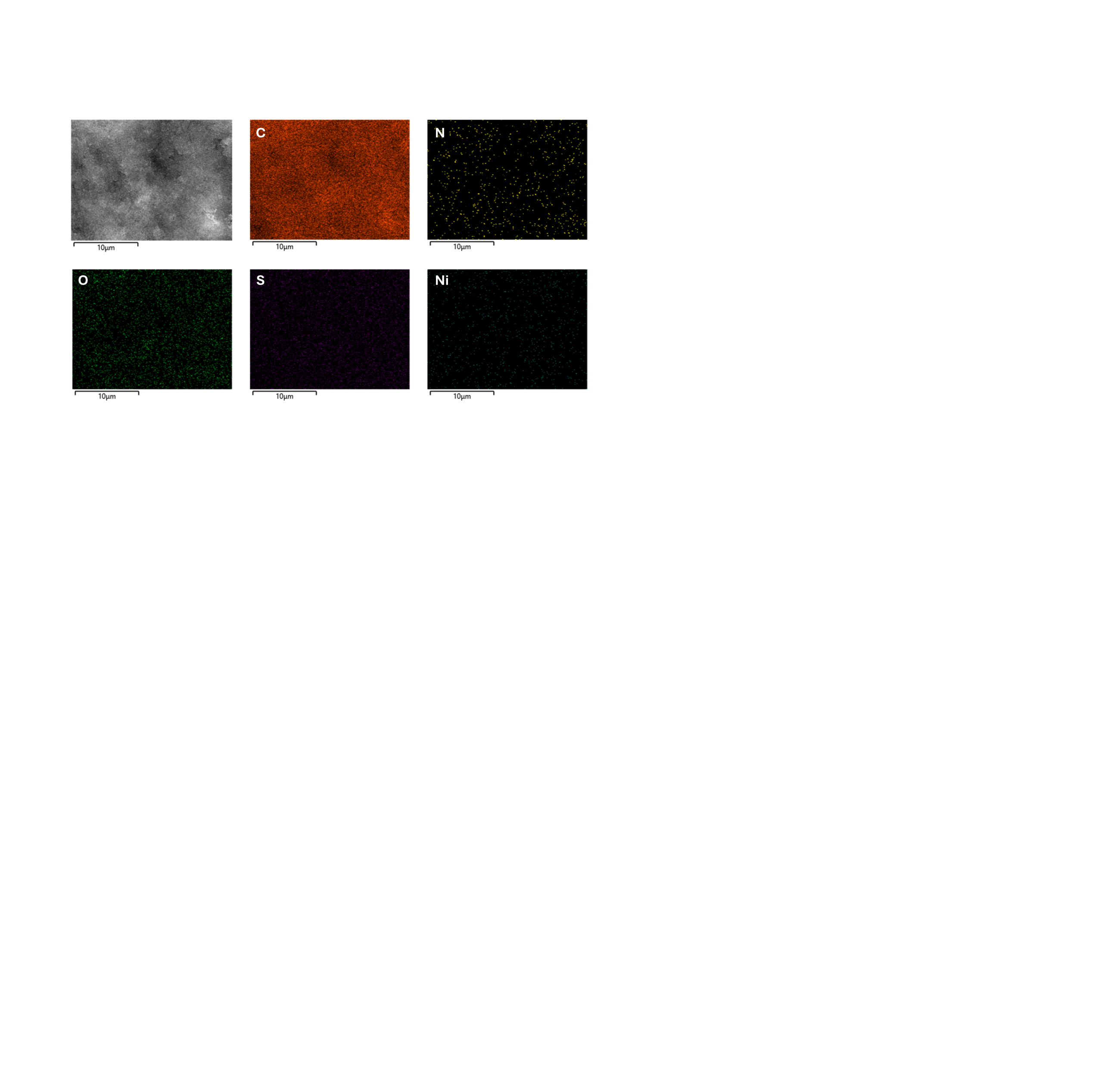


**Figure S30.** SEM image and EDS mapping (including C, N, O, S and Ni elements) of NiPcTs/CB electrode (soaking time: 80 min) after 1-hour electrocatalysis at -0.75 V vs RHE in H-cell.


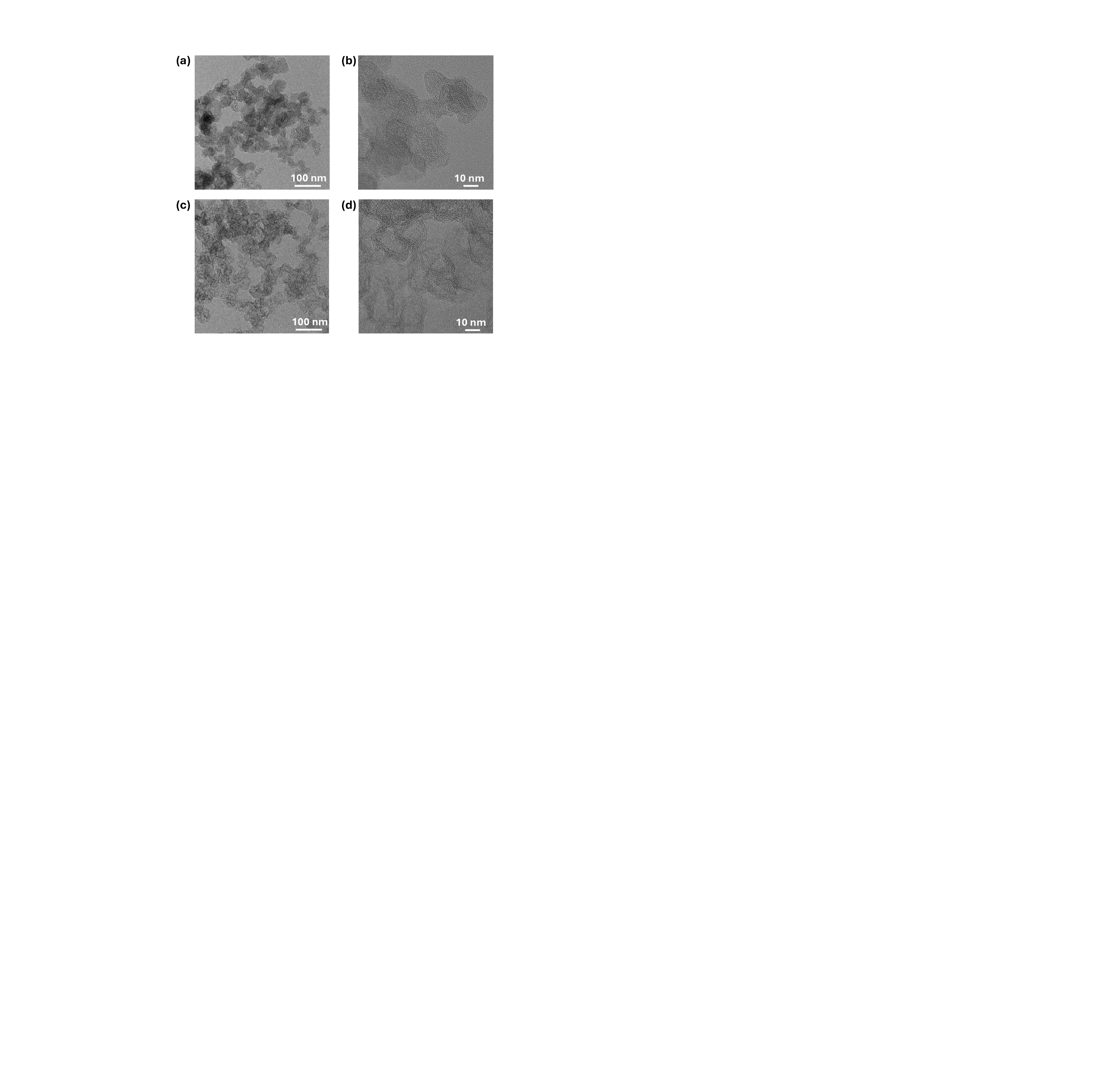


**Figure S31.** (a) Low-magnification and (b) high-magnification HR-TEM images of CoPcTs/CB (soaking time: 80 min) after 1-hour electrocatalysis at -0.75 V vs RHE in H-cell. (c) Low-magnification and (d) high-magnification HR-TEM images of NiPcTs/CB (soaking time: 80 min) after 1-hour electrocatalysis at -0.75 V vs RHE in H-cell.


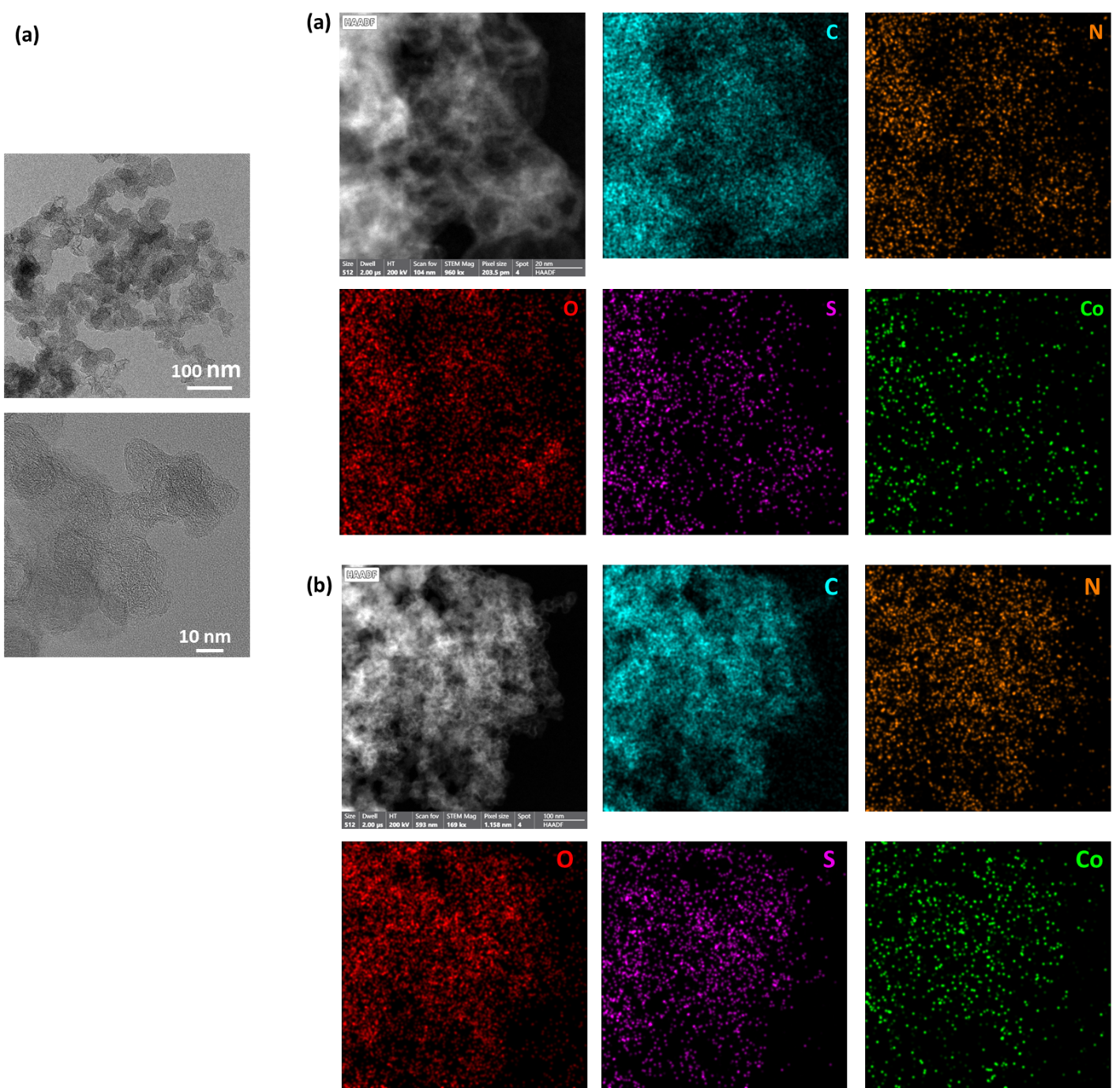


**Figure S32.** (a) Low-magnification and (b) high-magnification HRTEM-HAADF images and the corresponding EDS mapping (including C, N, O, S and Co elements) of CoPcTs/CB electrode (soaking time: 80 min) after 1-hour electrocatalysis at -0.75 V vs RHE in H-cell.


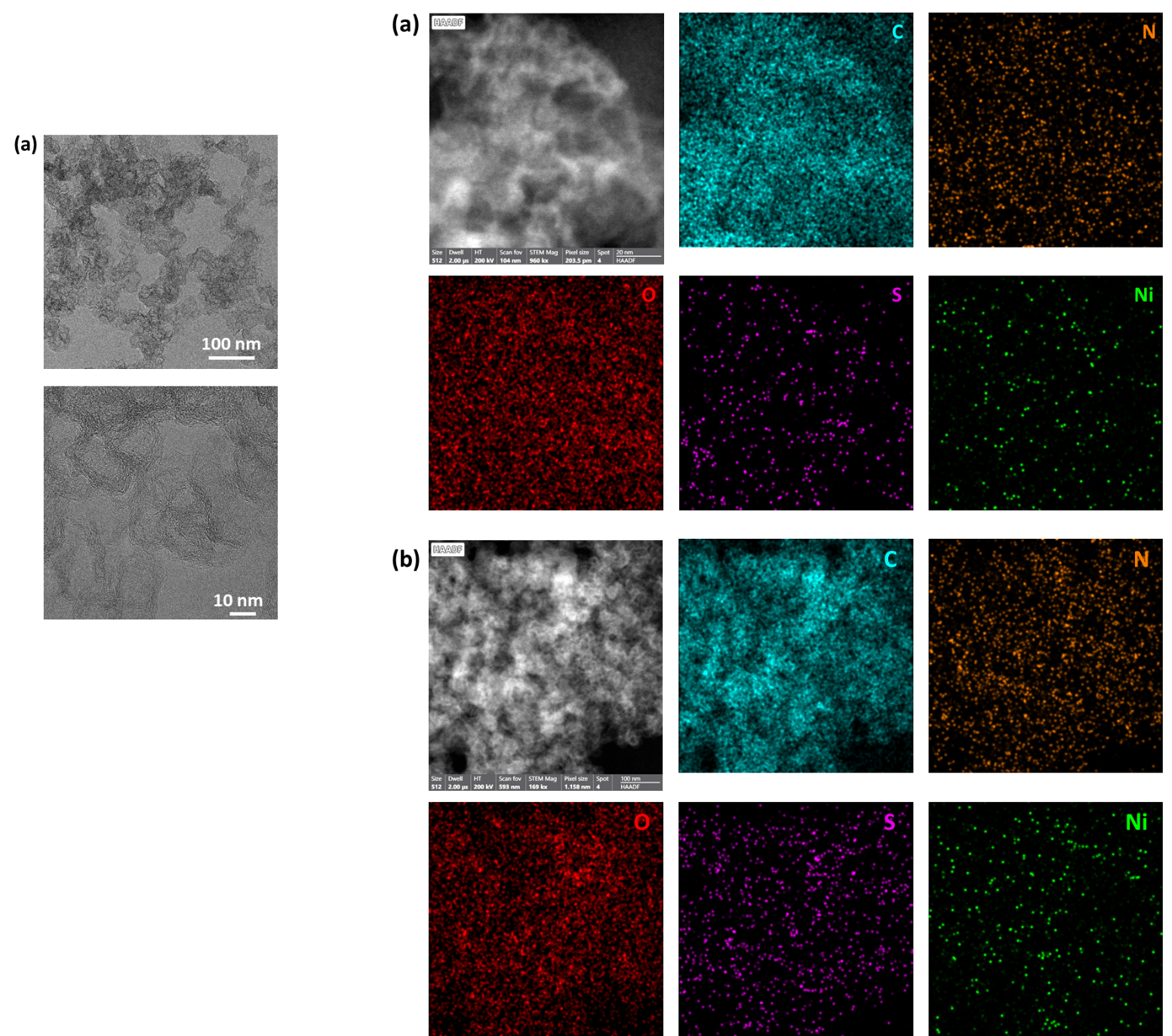


**Figure S33.** (a) Low-magnification and (b) high-magnification HRTEM-HAADF images and the corresponding EDS mapping (including C, N, O, S and Ni elements) of NiPcTs/CB electrode (soaking time: 80 min) after 1-hour electrocatalysis at -0.75 V vs RHE in H-cell.


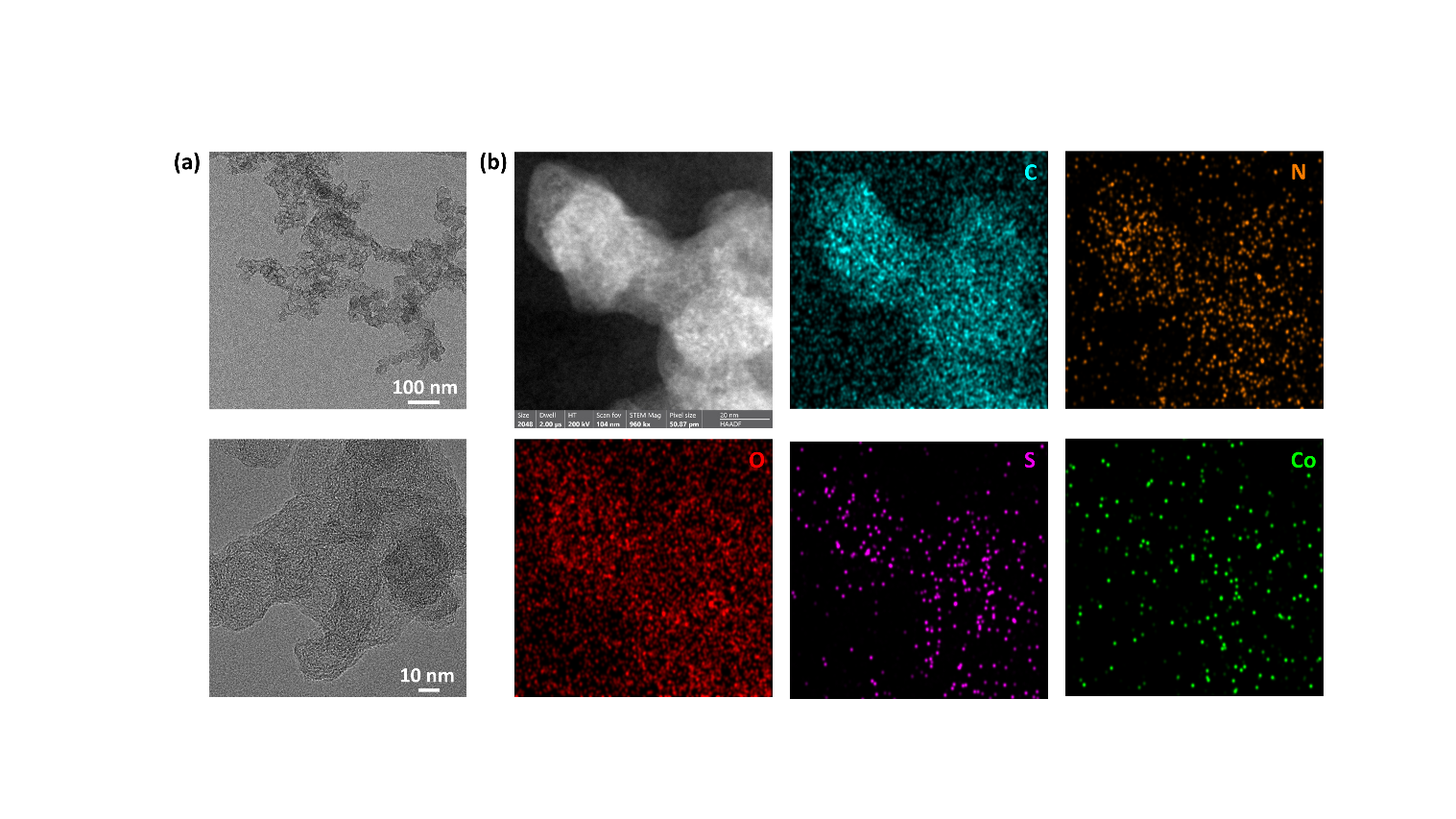


**Figure S34.** (a) Low-magnification and high-magnification HR-TEM images of CoPcTs/CB (soaking time: 80 min) after 1-hour electrocatalysis at 200 mA cm^-2^ in flow cell. (b) HRTEM-HAADF images and the corresponding EDS mapping (including C, N, O, S and Co elements) of CoPcTs/CB electrode (soaking time: 80 min) after 1-hour electrocatalysis at 200 mA cm^-2^ in flow cell.


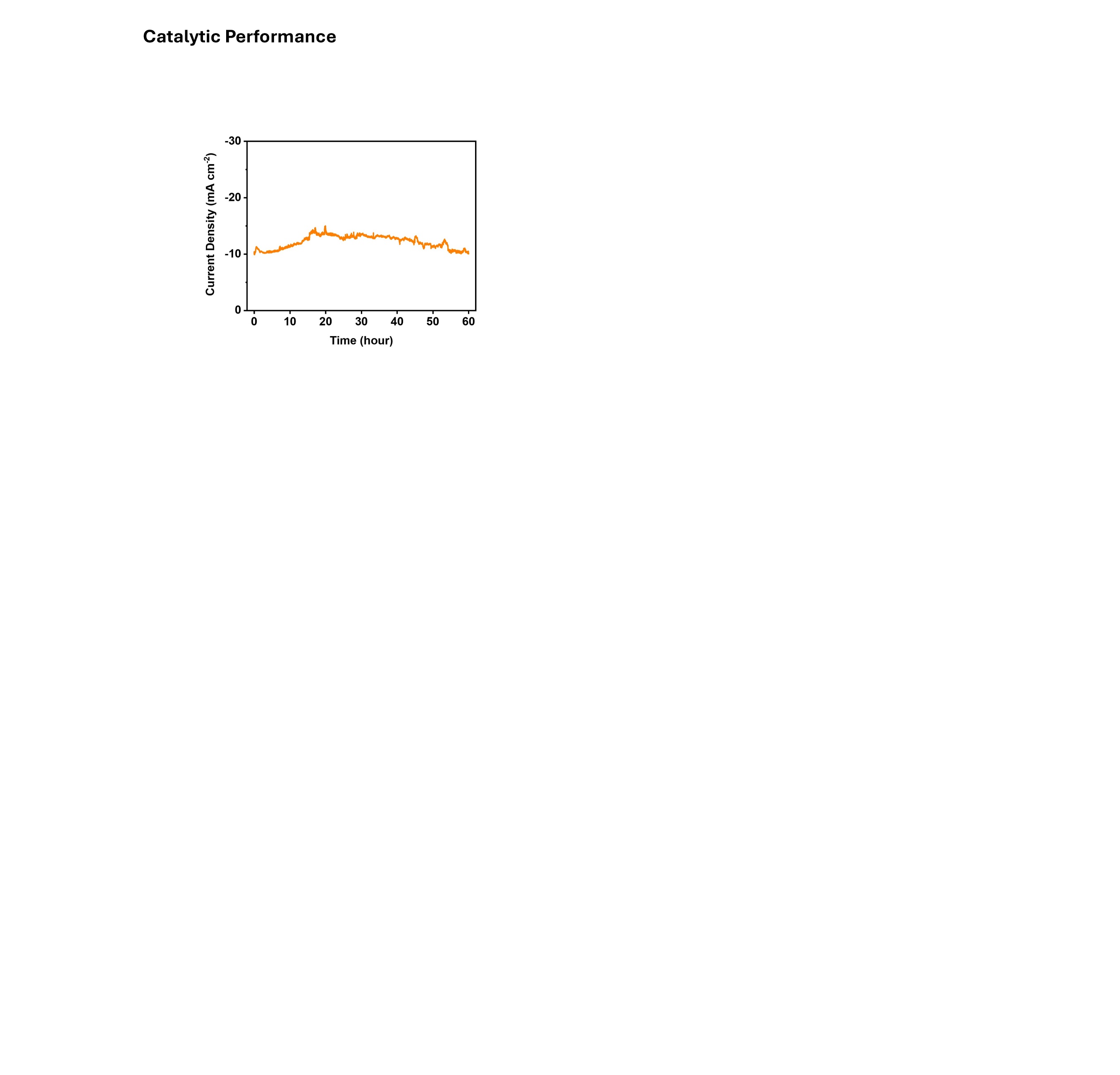


**Figure S35.** Current density of long-term stability measurement at −0.75 V vs RHE in CO_2_-saturated 0.5 M KHCO_3_ aqueous solution in H-cell.


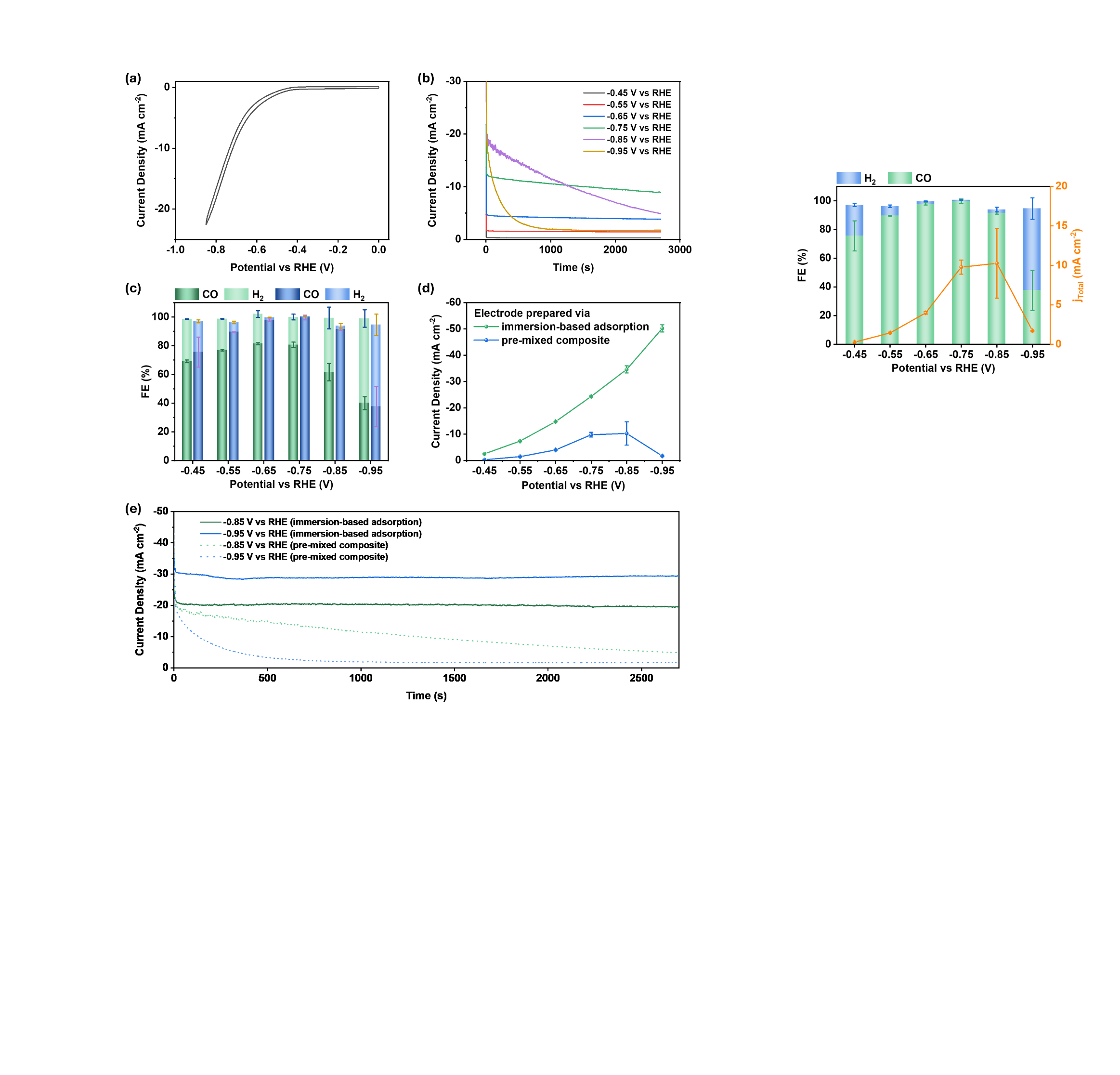


**Figure S36**. Performance of the pre-mixed composite. (a) Cyclic voltammograms and (b) current density–time relationship curves for the CoPcTs/CB catalyst (pre-mixed composite, mass ratio of CoPcTs/CB = 1:200, ensuring the same cobalt amount) prepared at different potentials vs RHE in an H-cell. The error bars indicate the variability among values from three repeated measurements. (c) Faradaic efficiencies for CO (FE_CO_) and H_2_ (FE_H2_) of the CoPcTs/CB catalyst prepared via immersion-based adsorption (green column) and pre-mixed composite method (blue column) at different potentials vs RHE in an H-cell. (d, e) Comparison of the current density and operational stability for electrodes prepared by *immersion-based adsorption* and *pre-mixed composite method*.


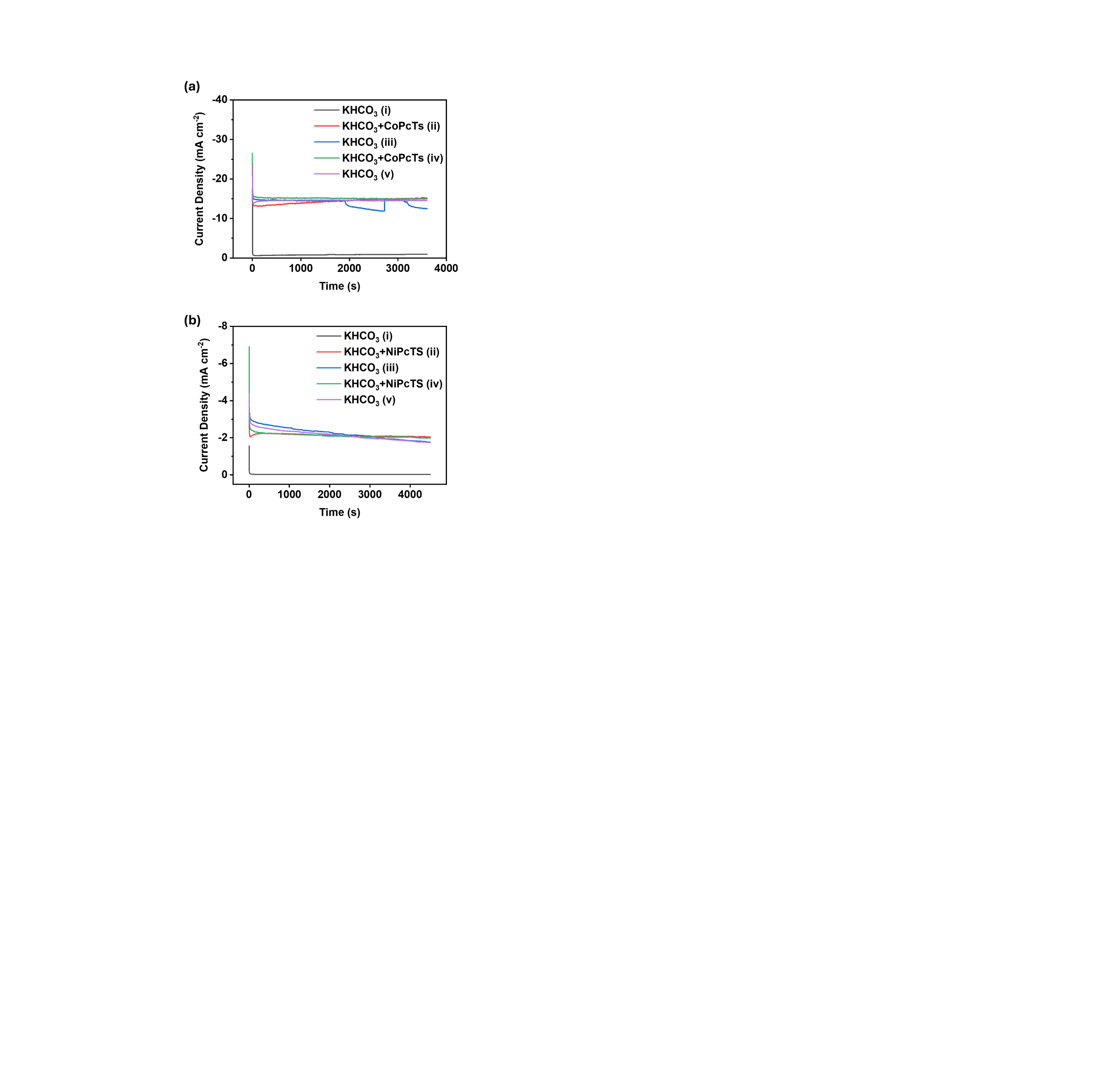


**Figure S37.** Current density−time relationship of a CB electrode in electrochemical CO_2_RR switching experiments with CO_2_-saturated 0.5 M KHCO_3_ solution (as serial number i, iii and v) and (a) a mixed solution of 0.5 M KHCO_3_ and 50 μM CoPcTs or (b) a mixed solution of 0.5 M KHCO_3_ and 50 μM NiPcTs (as serial number ii and iv) as electrolyte at a potential of –0.95 V vs RHE for CoPcTs and –0.85 V vs RHE for NiPcTs.


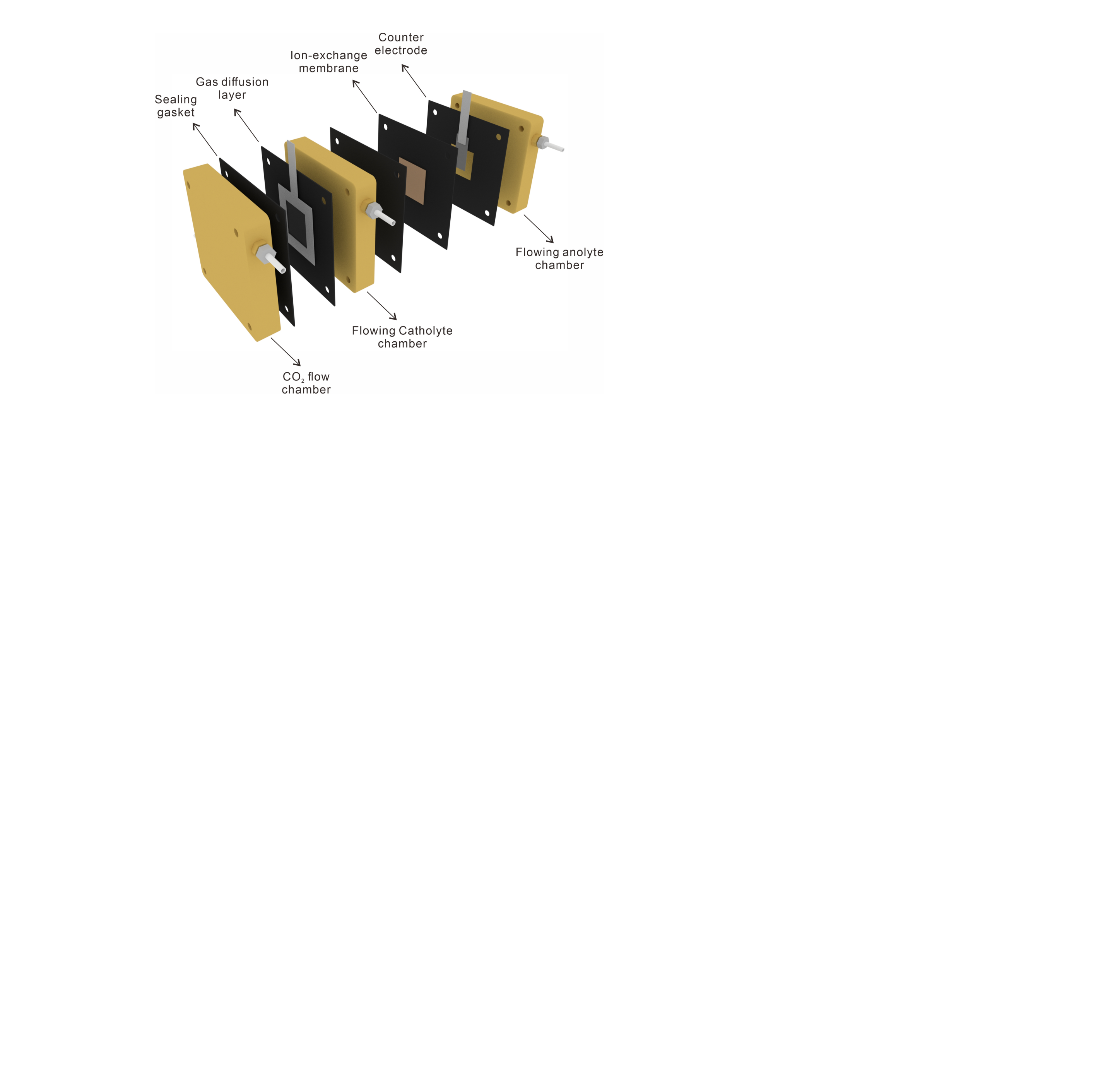


**Figure S38.** The schematic diagram of flow cell.


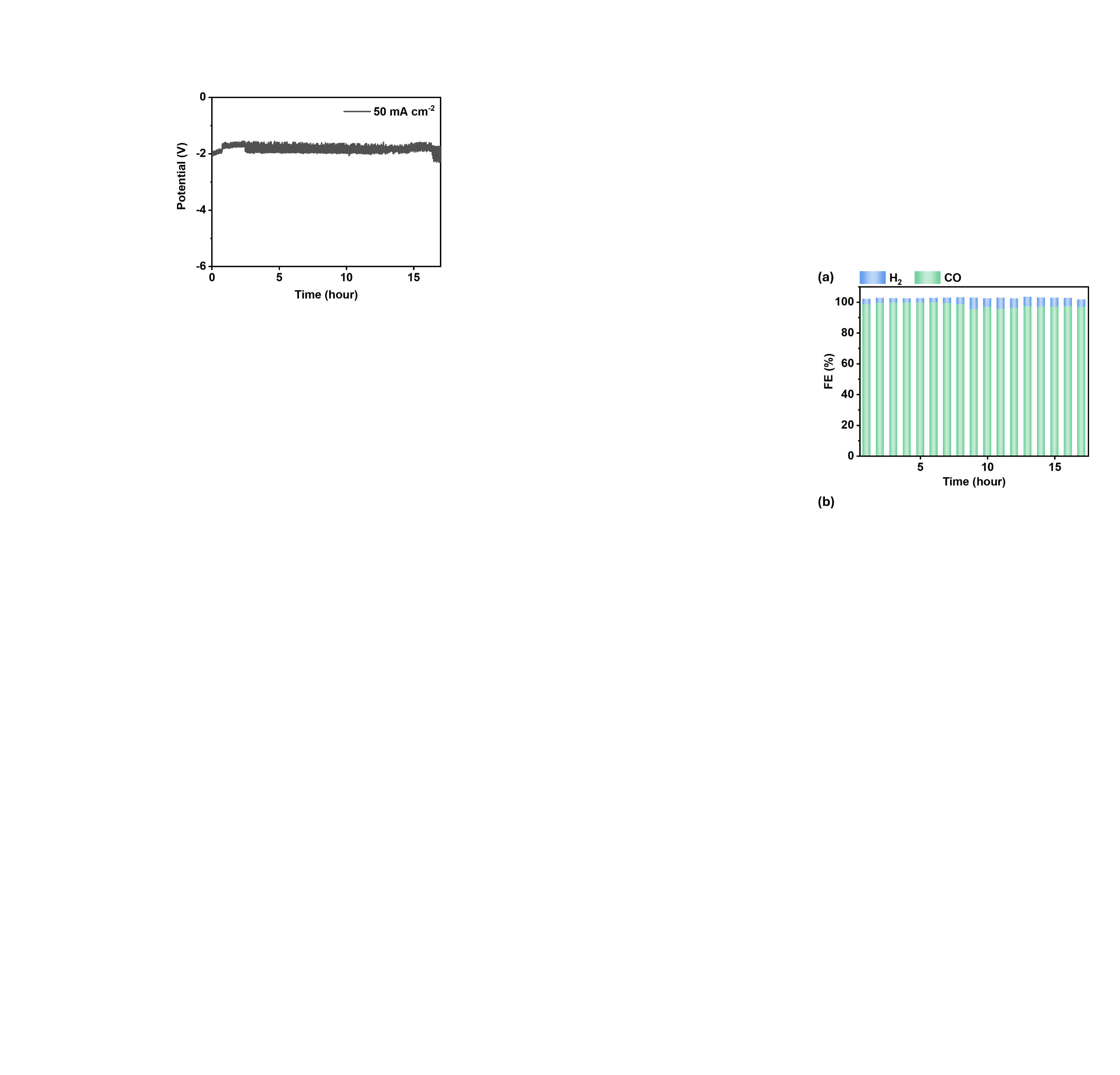


**Figure S39.** The potential-time curve of CoPcTs/CB in long-term stability measurement at 50 mA cm^−2^ in flow cell.


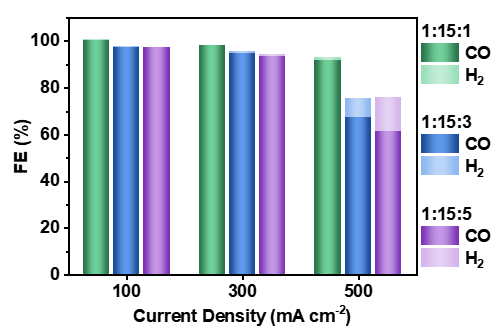


**Figure S40.** FE_CO_ and FE_H2_ of CoPcTs/CB/PTFE catalyst in 1-hour consecutive CO_2_RR process (mass ratio of catalyst, CB and PTFE = 1:15:1, 1:15:3 and 1:15:5) at 100 and 300 mA cm^−2^ in 1 M KHCO_3_ electrolytes in flow cell (the electrodes were prepared through pre-composite procedure).


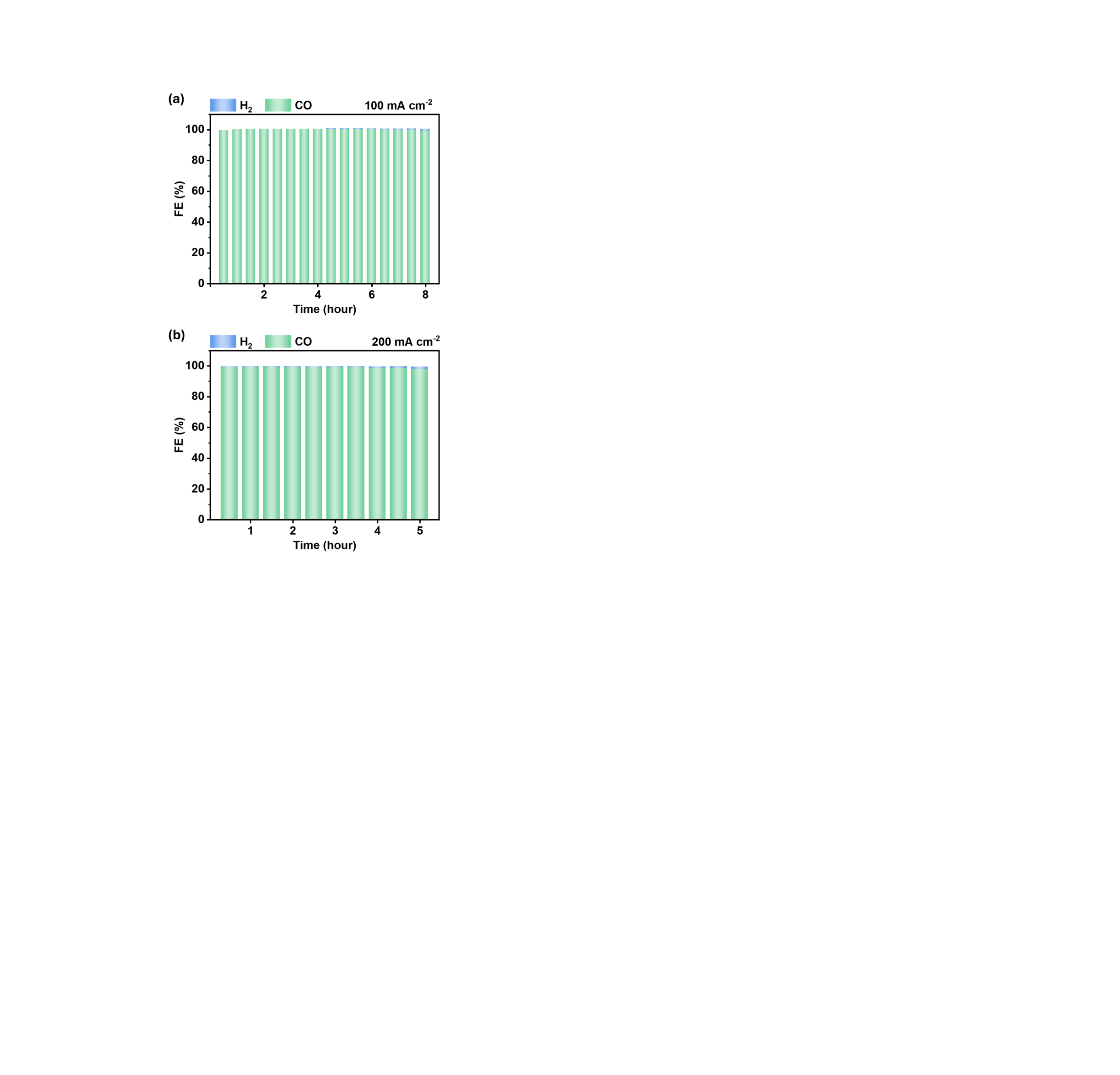


**Figure S41.** Long-term stability and corresponding FE_CO_ and FE_H2_ of CoPcTs/CB catalyst at (a) 100 mA cm^−2^ and (b) 200 mA cm^−2^ in flow cell.


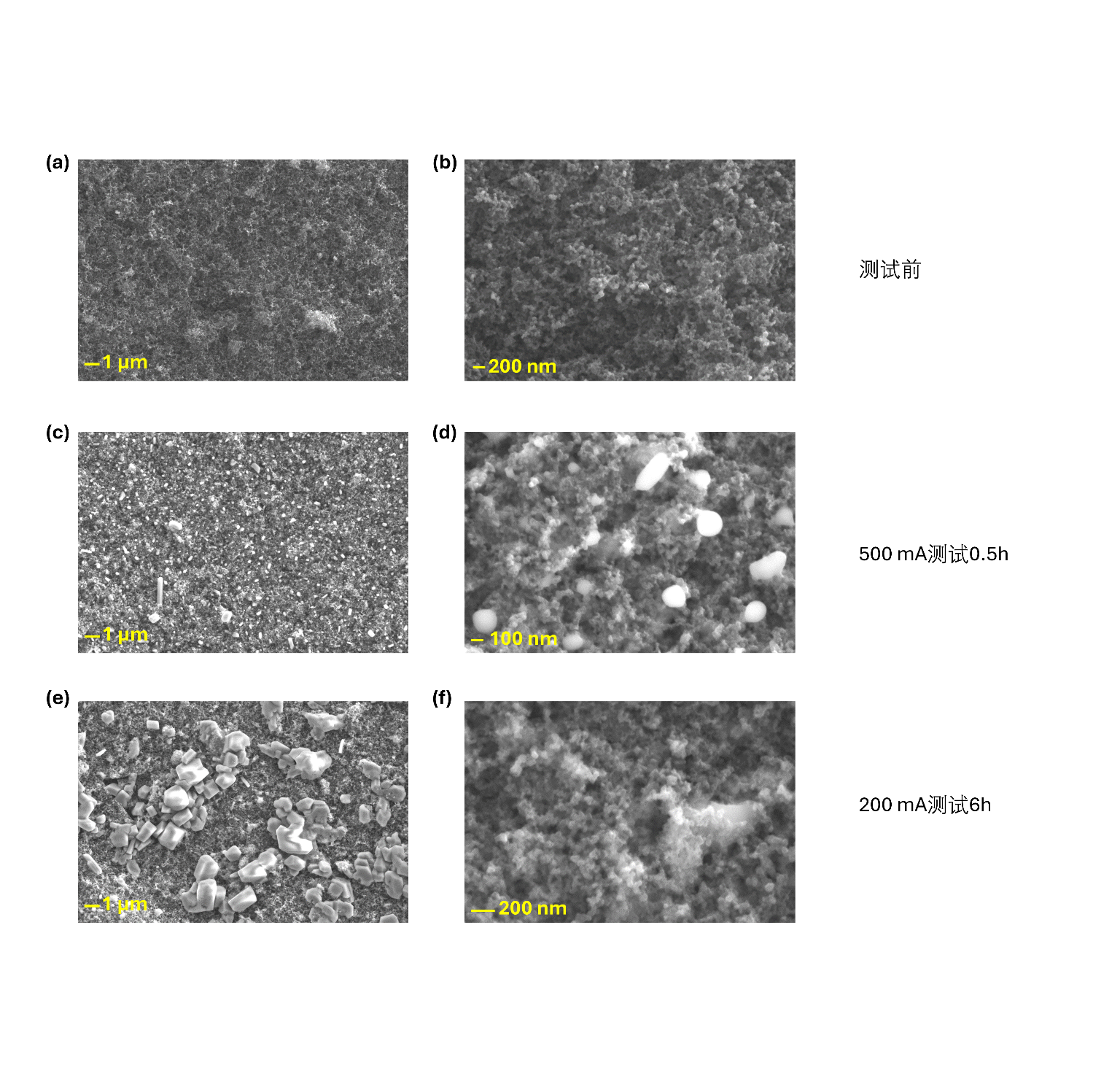


**Figure S42.** SEM images of CoPcTs/CB/PTFE=1:15:1 electrode.


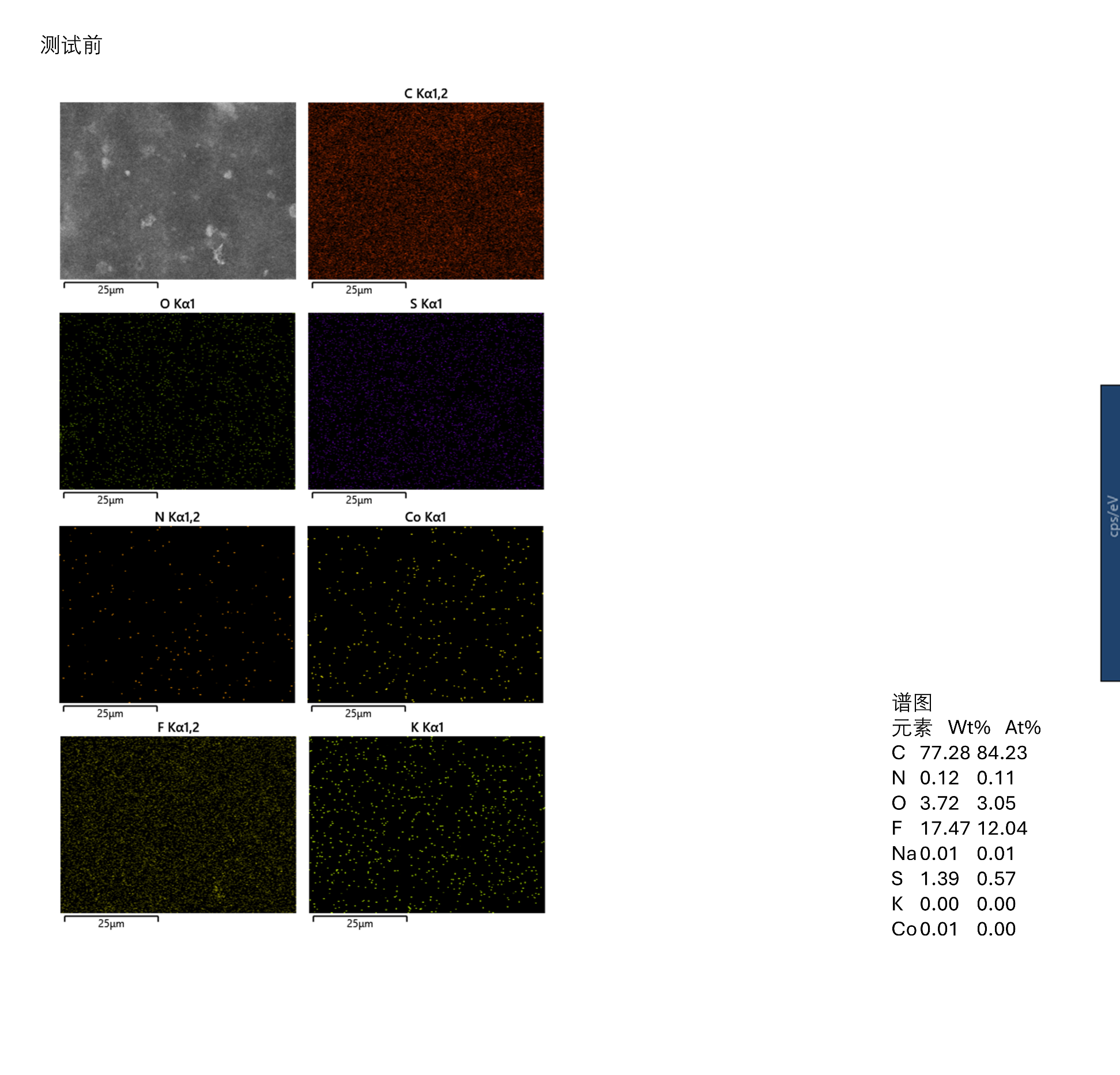


**Figure S43.** SEM image and EDS mapping (including C, O, S, N, Co, F and K elements) of CoPcTs/CB/PTFE=1:15:1 electrode.

**Table S1.** Ni K-edge EXAFS curves fitting parameters of NiPcTs and NiPcTs/CB; Co K-edge EXAFS curves fitting parameters of CoPcTs and CoPcTs/CB.

| Sample | Path | CN | R / Å | D-W factor (σ^2^) / Å^2^ | △E0 / eV | R-factor |
| --- | --- | --- | --- | --- | --- | --- |
| CoPcTs | Co-N1 | 3.23(0.38) | 1.90(0.01) | 0.002 | -7.23(1.59) | 0.01432 |
| CoPcTs/CB | Co-N1 | 3.49(0.65) | 1.89(0.01) | 0.002 | -6.95(2.41) | 0.03327 |
| NiPcTs | Ni-N1 | 3.96(1.09) | 1.89(0.02) | 0.002 | 2.54(3.47) | 0.01749 |
| NiPcTs/CB | Ni-N1 | 3.67(0.82) | 1.88(0.01) | 0.001 | 0.09(2.93) | 0.00676 |

**Table S2.** Performance of the best reported heterogeneous catalysts for CO_2_-to-CO conversion.

| Catalyst | J  (mA cm^-2^) | FE_CO_  (%) | TOF  (s^-1^) | Electrolyte | Ref. |
| --- | --- | --- | --- | --- | --- |
| CoPc/CNT | −15.0^§^ | 98.0 | 4.1 | 0.1 M KHCO_3_ | [9] |
| CoPc1 | −13.1^#^ | 92.0 | 4.08 | 0.5 M NaHCO_3_ | [10] |
| CoPc2 | −18.1^#^ | 93.0 | 6.81 | 0.5 M NaHCO_3_ | [10] |
| CoPc2^*^ | −165^#^ | 94.0 | 3.9 | 0.5 M NaHCO_3_ | [10] |
| CoPc-4OCH3@CNTs | −48.7^#^ | 91.0 | ~9.8 | 0.5 M KHCO_3_ | [11] |
| CoPc-4NO2@CNTs | −34.3^#^ | 90.0 | ~8.0 | 0.5 M KHCO_3_ | [11] |
| CoPc@CNTs | −17.6^#^ | 89.0 | ~8.1 | 0.5 M KHCO_3_ | [11] |
| CoPPC/CNT | −18.7^#^ | 90.0 | ~1.36 | 0.5 M NaHCO_3_ | [12] |
| TC-CoPc/MWCNTs | −22.6^#^ | 95.6 | 29.4 | 0.5 M KHCO_3_ | [13] |
| CoPc/NH2  -CNT | −16.3^#^ | 92.2 | 31.4 | 0.5 M KHCO_3_ | [14] |
| CoPc/COOH-CNT | −9.3^#^ | 88.2 | 18.1 | 0.5 M KHCO_3_ | [14] |
| CoPc/OH-CNT | −11.3^#^ | 88.5 | 21.7 | 0.5 M KHCO_3_ | [14] |
| CoPc/CNT | −6.1^#^ | 73.3 | 11.7 | 0.5 M KHCO_3_ | [14] |
| CoPc@DNHCS-8 | −16.5^#^ | 95.7 | 13.8 | 0.5 M NaHCO_3_ | [15] |
| CCG/CoPc-A hybrid | ~−1.0^§^ | 0.77 | 5.0 | 0.1 M KHCO_3_ | [16] |
| CoPc-py-  CNT | ~−5.7^§^ | 98.0 | 34.5 | 0.2 M NaHCO_3_ | [17] |
| CoT-NH_2_ | ~−1.0^#^ | 95.0 | 4.37 | 0.1 M KHCO_3_ | [18] |
| CoPc-NVG/CC | −46.1^#^ | 99.0 | 9.9 | 0.5 M KHCO_3_ | [19] |
| p(CoPc-1) | −2.2^#^ | 94.0 | 0.29 | 0.5 M KHCO_3_ | [20] |
| CoPc@N-CA-500 | −21.72^#^ | 92.5 | 1.23 | 0.5 M KHCO_3_ | [21] |
| CoPc-PDQ-COF | −22.2^§^ | 96.0 | 3.17 | 0.5 M KHCO_3_ | [22] |
| COF@CoPor | −12.5^#^ | 73.8 | 1.27 | 0.5 M KHCO_3_ | [23] |
| CoPc-PI-COF-1 | −9.4^#^ | 93.0 | 2.2 | 0.5 M KHCO_3_ | [24] |
| CoPc-PI-COF-2 | −6.2^#^ | 93.0 | 1.9 | 0.5 M KHCO_3_ | [24] |
| CoPc/GDY/G | −2.2^§^ | 96.0 | 9.1 | 0.1 M KHCO_3_ | [25] |
| CoPc/GDY/G | −9.0^§^ | 96.0 | 37 | 0.1 M KHCO_3_ | [25] |
| CoPc/GDY/G^*^ | −100^§^ | 97.0 | 28 | 0.1 M KHCO_3_ | [26] |
| CoN-7 | −21.4^#^ | 97.3 | 0.94 | 0.5 M KHCO_3_ | [26] |
| N-CoMe2Pc/NRGO (6:10)^*^ | −56.4^#^ | 94.1 | 6.2 | 1.0 M KOH | [27] |
| CNT@CMP^*^ | −250^§^ | 96.0 | 27.1 | 1.0 M KHCO_3_ | [28] |
| STPyP-Co | −6.6^§^ | 90.0 | 4.21 | 0.5 M KHCO_3_ | [29] |
| **CoPcTs/CB^*^** | **−160.0**^#^ | **80.0** | **42.30** | **0.5 M KHCO_3_** | **This work** |

*^*^ Folw Cell;*

*^#^ Partial Current Density;*

*^§^ Total Current Density;*

Reference

[1] Gaussian 16, Revision A.03, M. J. Frisch, G. W. Trucks, H. B. Schlegel, G. E. Scuseria, M. A. Robb, J. R. Cheeseman, G. Scalmani, V. Barone, G. A. Petersson, H. Nakatsuji, X. Li, M. Caricato, A. V. Marenich, J. Bloino, B. G. Janesko, R. Gomperts, B. Mennucci, H. P. Hratchian, J. V. Ortiz, A. F. Izmaylov, J. L. Sonnenberg, D. Williams-Young, F. Ding, F. Lipparini, F. Egidi, J. Goings, B. Peng, A. Petrone, T. Henderson, D. Ranasinghe, V. G. Zakrzewski, J. Gao, N. Rega, G. Zheng, W. Liang, M. Hada, M. Ehara, K. Toyota, R. Fukuda, J. Hasegawa, M. Ishida, T. Nakajima, Y. Honda, O. Kitao, H. Nakai, T. Vreven, K. Throssell, J. A. Montgomery, Jr., J. E. Peralta, F. Ogliaro, M. J. Bearpark, J. J. Heyd, E. N. Brothers, K. N. Kudin, V. N. Staroverov, T. A. Keith, R. Kobayashi, J. Normand, K. Raghavachari, A. P. Rendell, J. C. Burant, S. S. Iyengar, J. Tomasi, M. Cossi, J. M. Millam, M. Klene, C. Adamo, R. Cammi, J. W. Ochterski, R. L. Martin, K. Morokuma, O. Farkas, J. B. Foresman, and D. J. Fox, Gaussian, Inc., Wallingford CT, **2016**.

[2] Adamo C., Barone V., *J. Chem. Phys.* **1999**, *110*, 6158–6170.

[3] Grimme S., Ehrlich S., Goerigk L., *J. Comput. Chem.* **2011**, *32*, 1456–1465.

[4] Steinmetz M., Grimme S., *ChemistryOpen* **2013**, *2*, 115–124.

[5] Schafer A., Huber C., Ahlrichs R., *J. Chern. Phys.* **1992***,* *97*, 2571–2577.

[6] Schafer A., Huber C., Ahlrichs R., *J. Chern. Phys.* **1994**, *100*, 5829–5835.

[7] Lu T., Chen Q., *Comput. Theor. Chem.* **2021**, *1200*, 113249.

[8] Chan, K., Nørskov, J. K., *J. Phys. Chem. Lett.* **2015**, *6*, 2663–2668.

[9] Zhang, X.; Wu, Z.; Zhang, X.; Li, L.; Li, Y.; Xu, H.; Li, X.; Yu, X.; Zhang, Z.; Liang, Y.; Wang, H., *Nat. Commun.* **2017,** *8*, 14675.

[10] Wang, M.; Torbensen, K.; Salvatore, D.; Ren, S.; Joulie, D.; Dumoulin, F.; Mendoza, D.; Lassalle-Kaiser, B.; Isci, U.; Berlinguette, C. P.; Robert, M., *Nat. Commun.* **2019,** *10* (1), 3602.

[11] Huang, M.; Chen, B.; Zhang, H.; Jin, Y.; Zhi, Q.; Yang, T.; Wang, K.; Jiang, J., *Small Methods* **2025,** 2301652.

[12] Han, N.; Wang, Y.; Ma, L.; Wen, J.; Li, J.; Zheng, H.; Nie, K.; Wang, X.; Zhao, F.; Li, Y.; Fan, J.; Zhong, J.; Wu, T.; Miller, D. J.; Lu, J.; Lee, S.-T.; Li, Y., *Chem* **2017**, *3* (4), 652−664.

[13] Zhou, S.; Zhang, L. J.; Zhu, L.; Tung, C. H.; Wu, L. Z., *Adv. Mater.* **2023,** *35* (41), e2300923.

[14] Li, H.; Pan, Y.; Wang, Z.; Yu, Y.; Xiong, J.; Du, H.; Lai, J.; Wang, L.; Feng, S., *Nano Res.* **2021,** *15* (4), 3056−3064.

[15] Gong, S.; Wang, W.; Zhang, C.; Zhu, M.; Lu, R.; Ye, J.; Yang, H.; Wu, C.; Liu, J.; Rao, D.; Shao, S.; Lv, X., *Adv. Func. Mater.* **2022,** *32* (17), 2110649.

[16] Choi, J.; Wagner, P.; Gambhir, S.; Jalili, R.; MacFarlane, D. R.; Wallace, G. G.; Officer, D. L., *ACS Energy Lett.* **2019,** *4* (3), 666−672.

[17] Zhu, M.; Chen, J.; Guo, R.; Xu, J.; Fang, X.; Han, Y.-F., *Appl. Catal., B* **2019,** *251*, 112−118.

[18] Zhou, Y.; Duan, X.; Xu, X.; Ei Phyu Win, P.; Ren, S.-B.; Wang, J., *Chem. Mater.* **2024,** *37* (1), 360−367.

[19] Kong, X.; Liu, G.; Tian, S.; Bu, S.; Gao, Q.; Liu, B.; Lee, C. S.; Wang, P.; Zhang, W., *Small* **2022,** *18* (51), 2204615.

[20] Luangchaiyaporn, J.; Wielend, D.; Solonenko, D.; Seelajaroen, H.; Gasiorowski, J.; Monecke, M.; Salvan, G.; Zahn, D. R. T.; Sariciftci, N. S.; Thamyongkit, P., *Electrochim. Acta* **2021,** *367*, 137506.

[21] Zhang, B.; Gong, S.; Wang, G.; Wu, C.; Zhao, G.; Lv, X., *Appl. Surf. Sci.* **2023,** *630*, 157437.

[22] Huang, N.; Lee, K. H.; Yue, Y.; Xu, X.; Irle, S.; Jiang, Q.; Jiang, D., *Angew. Chem. Int. Ed.* **2020,** *59* (38), 16587−16593.

[23] Zhai, L.; Yang, S.; Lu, C.; Cui, C. X.; Xu, Q.; Liu, J.; Yang, X.; Meng, X.; Lu, S.; Zhuang, X.; Zeng, G.; Jiang, Z., *Small* **2022,** *18* (32), e2200736.

[24] Han, B.; Ding, X.; Yu, B.; Wu, H.; Zhou, W.; Liu, W.; Wei, C.; Chen, B.; Qi, D.; Wang, H.; Wang, K.; Chen, Y.; Chen, B.; Jiang, J., *J. Am. Chem. Soc.* **2021,** *143* (18), 7104−7113.

[25] Gu, H.; Zhong, L.; Shi, G.; Li, J.; Yu, K.; Li, J.; Zhang, S.; Zhu, C.; Chen, S.; Yang, C.; Kong, Y.; Chen, C.; Li, S.; Zhang, J.; Zhang, L., *J. Am. Chem. Soc.* **2021,** *143* (23), 8679−8688.

[26] Liu, C.; Bian, Z.; Wang, W.; Li, H.; Dang, D.; Bai, Y.,  *Appl. Surf. Sci.* **2024,** *663*, 160192.

[27] Li, M.; Yan, C.; Ramachandran, R.; Lan, Y.; Dai, H.; Shan, H.; Meng, X.; Cui, D.; Wang, F.; Xu, Z.-X.,  *Chem. Eng. J.* **2022,** *430*, 133050.

[28] Wang, R.; Wang, X.; Weng, W.; Yao, Y.; Kidkhunthod, P.; Wang, C.; Hou, Y.; Guo, J., *Angew. Chem. Int. Ed.* **2021,** *61* (5), e202115503.

[29] Han, J.; An, P.; Liu, S.; Zhang, X.; Wang, D.; Yuan, Y.; Guo, J.; Qiu, X.; Hou, K.; Shi, L.; Zhang, Y.; Zhao, S.; Long, C.; Tang, Z., *Angew. Chem. Int. Ed.* **2019,** *58* (36), 12711−12716.
